# Measuring health-related quality of life in a Chinese Mainland adolescent population: psychometric properties of the Mandarin Chinese self-reported KIDSCREEN-27 and KIDSCREEN-10 index

**DOI:** 10.1186/s40359-024-01876-6

**Published:** 2024-10-29

**Authors:** Juan Li, Yuhang Zhu, Gaopei Zhu, Zhenliang Qiu, Jinling Wang, Anne Kaman, Michael Erhart, Adekunle Adedeji, Yongye Liu, Di Wu, Ulrike Ravens-Sieberer, the WFMU-KS-MC research group, Juan Li, Yuhang Zhu, Di Wu, Johan Yau Yin Ng, Yinghua Ma, Hanrong Wu, Yizhen Yu, Yuantao Hao, Hongmei Wang, Huijing Shi, Taisheng Cai, Yanbo Zhu, Zaohuo Cheng, Suzhen Wang, Wenqiang Yin, Dongmei Huang, Haojia Chen, Jizhi Guo, Shanju Hu, Fuhao Huo, Pengyu Lou, Qingduo Mao, Johan Yau Yin Ng, Mengqi Tang, Ruimei Wang, Min Wang

**Affiliations:** 1 Department of Health Management, School of Health Management, Binzhou Medical University, No. 346, Guanhai Road, Laishan, Yantai, Shandong 264003 China; 2 Teaching and Research Section of Health Statistics, School of Public Health, Shandong Second Medical University, No. 7166 Baotong West Street, Weicheng, Weifang, Shandong 261053 China; 3 Department of Health Statistics, School of Public Health, Cheeloo College of Medicine, Shandong University, No. 44 Wenhua West Road, Lixia, Jinan, Shandong 250012 China; 4 No. 1 High School of Anhui Sixian County, No. 172 Sishui Boulevard, Hongcheng Sub-district, Sixian, Suzhou, Anhui 234300 China; 5 Weifang Dongming School, No. 2933 Beigong East Street, Kuiwen, Weifang, Shandong 261061 China; 6 Department of Child and Adolescent Psychiatry, Psychotherapy, and Psychosomatics, Center for Psychosocial Medicine, University Medical Center Hamburg-Eppendorf, Martinistraße 52, W 29, Hamburg, 20246 Germany; 7 Faculty of Life Sciences, Hamburg University of Applied Sciences, Ulmenliet 20, Hamburg, 21033 Germany; 8 Urban Governance and Design Thrust, Society Hub, The Hong Kong University of Science and Technology (Guangzhou), No. 1 Duxue Road, Nansha, Guangzhou, Guangdong 511458 China; 9 The Second Teaching and Research Section of English Major, School of Foreign Languages, Shandong Second Medical University, No. 7166 Baotong West Street, Weicheng, Weifang, Shandong 261053 China

**Keywords:** Adolescents, Health-related quality of life, Mandarin Chinese, Psychometric properties, Self-reported, KIDSCREEN-27, KIDSCREEN-10 index

## Abstract

**Background:**

The self-reported KIDSCREEN questionnaires are ideal for capturing children’s and adolescents’ health-related quality of life (HRQoL) and have gained worldwide popularity. Responding to demands for the concise nature of KIDSCREEN among Chinese Mainland researchers and practitioners, this study aimed to evaluate the psychometric properties of the Mandarin Chinese self-reported KIDSCREEN-27 (KS-27) and KIDSCREEN-10 index (KS-10), which are short versions of the self-reported KIDSCREEN-52 (KS-52).

**Methods:**

This study reanalyzed the validation dataset of the Mandarin Chinese self-reported KS-52. The dataset originated from a cross-sectional survey conducted in Weifang City, the Chinese Mainland, from October to November 2016. Adolescents aged 11–17 years (*N* = 4385) were surveyed, and sub-samples (*N* = 841) were retested. Statistical analyses were conducted on the feasibility, item and dimension properties, reliability, and validity of the Mandarin Chinese self-reported KS-27 and KS-10.

**Results:**

Indirect evidence from the Small Group Pilot Survey indicated that the Mandarin Chinese self-reported KS-27 and KS-10 can be completed on average in less than 12.33 mins. Their response rate exceeded 90% regardless of the unit- and item (dimension)-level. The psychometric properties of items and dimensions were likewise found to be satisfactory. Internal consistency was robust with inter-item and item-total correlations (0.173–0.873, 0.422–0.786), Cronbach’s alpha (0.786–0.881), Guttman’s lambda-2 and - 6 (0.807–0.889, 0.829–0.896), and McDonald’s omega (0.725–0.886). Test–retest reliability at both item- and dimension-level was excellent, with intraclass correlation coefficients (ICCs) of (0.590–0.696, 0.785–0.842); standard error of measurements (*SEM*s) of (0.352–0.635, 0.949–1.949). Confirmatory factor analysis (CFA) confirmed their five- and one-dimensional structures, albeit with slight modifications. Moreover, the multi-group confirmatory factor analysis (MG-CFA) substantiated their configural and metric invariance across gender and grade groups. Convergent validity was robust, with stronger correlations observed with comparable dimensions of the Mandarin Chinese self-reported PedsQL™ 4.0, while discriminant validity was evident with low correlations observed with less comparable dimensions. The known-group validity was mainly supported by medium to large effect sizes concerning differences in socioeconomic status (η^2^ = 0.07–0.17, Cohen’s *d* = 0.55–1.03) and mental health status (η^2^ = 0.09–0.40, Cohen’s *d* = 0.73–1.83). The Mandarin Chinese self-reported KS-52 served as the criterion; the ICCs between the Mandarin Chinese self-reported KS-27 and KS-10 and their comparable dimensions were stronger, indicating robust criterion validity.

**Conclusions:**

The Mandarin Chinese self-reported KS-27 and KS-10 demonstrated excellent psychometric properties, indicating their good potential for measuring HRQoL for children and adolescents in the Chinese Mainland.

**Supplementary Information:**

The online version contains supplementary material available at 10.1186/s40359-024-01876-6.

## Background

“Ensure healthy lives and promote well-being for all at all ages” is one of the 17 Sustainable Development Goals (SDGs) by 2023 [1]. Health-related quality of life (HRQoL) in children and adolescents is crucial for achieving this target [2, 3]. In academia, as a subjective, multidimensional, and dynamic health screening indicator [4, 5], HRQoL plays an increasingly important role in children’s and adolescents’ clinical trials, epidemiology, and health policy [6–8].

It is well known that measurement is the first step in any scientific research [9, 10]. With the advancement of HRQoL research in children and adolescents, many generic HRQoL instruments specifically tailored for this population have emerged [4, 11]. These generic HRQoL instruments have a comprehensive application; they can be used in clinical practice, academic research, health policy evaluation, and public health surveillance to measure overall well-being and functioning among children and adolescents [4, 12–16]. Most of them are in the form of scales or questionnaires, and the well-known ones are the Youth Quality of Life-Research version (YQOL-R) [17, 18], the Child Health Questionnaire™ (CHQ) [19], and the KIDSCREEN questionnaires [20].

The KIDSCREEN questionnaires are typical representatives of the above generic HRQoL instruments. They have been developed and tested simultaneously in a large representative sample across several different European countries, thereby helping to provide a broad perspective on the understanding and interpretation of HRQoL [20]. This broad perspective not only covers physical, psychological, and social, but also creatively adds other unique new aspects, such as self-perception, autonomy, social acceptance (bullying), and financial resources [21]. The KIDSCREEN questionnaires are available in two informant forms: self-reported and proxy-reported [20]. This study only concentrates on the former. Several empirical studies have shown that children can adequately self-report their health from age eight if their emotional development, cognitive capacity, and reading skills are considered [22, 23]. Besides, the children’s and adolescents’ self-reports are consistent with the definition of HRQoL, which emphasizes the subjective perspective. Overall, the self-reported form is an ideal way to capture the children’s and adolescents’ HRQoL and should be recommended [20, 24].

Initially, to suit different application scenarios, researchers developed three versions of KIDSCREEN, namely KIDSCREEN-52 (KS-52), KIDSCREEN-27 (KS-27), and KIDSCREEN-10 index (KS-10) [20]. The original long KIDSCREEN version, KS-52 (52 items, ten dimensions), is recommended for in-depth thematic studies on HRQoL or when comprehensive and detailed profile information is required [3, 20]. It meets the eight critical attributes of a reasonable conceptual and measurement model, high reliability, good validity, excellent responsiveness, meaningful interpretability, low burden, alternative means of administration, and achieved appropriate cultural and language adaptations proposed by the Scientific Advisory Committee of the Medical Outcome Trust [25]. However, its slightly larger number of 50 items may pose risks, such as low efficiency and overloading of respondents [26, 27]. Empirical research indicates that short instruments can lessen the data collection burden, decrease non-response risks, and broaden their applicability in research and practice [28–32]. Hence, to enhance efficiency, lessen response burden, and save administration costs [27, 33], many researchers advocate developing and using short HRQoL instruments, particularly in epidemiological and clinical studies [34]. However, developing short versions comes with several challenges. These challenges include maintaining content and domain from the original long version without compromising psychometric performance, and simultaneously maximizing shared variance between short and long versions [35, 36]. Fortunately, both short KIDSCREEN versions meet the above requirements, each with different application scenarios. The KS-27 (27 items, five dimensions) minimizes information loss and exhibits excellent psychometric properties, suitable for broader epidemiological studies and screening among children and adolescents with chronic illnesses [20]. Furthermore, the KS-10 (10 items, one dimension), derives a “Global HRQoL Score (GHS),” recommended for practical use in screening studies or large-scale surveys [30].

So far, the KIDSCREEN questionnaires have been successfully translated into dozens of different languages in Europe, North America and Latin America, Africa, Asia, and Oceania, and its excellent psychometric properties have also been confirmed [37, 38]. The self-reported form of these questionnaires was the most popular. A systematic review of the KIDSCREEN questionnaires published in 2023 found that out of 253 reports worldwide, 89.3% (226/253) used the self-reported form [3]. It is worth noting that both short KIDSCREEN versions have demonstrated significant academic value and application potential. For example, the KS-10 is endorsed by the International Consortium for Health Outcomes Measurement (ICHOM) as part of the standard set of outcome measures for anxiety, depression, obsessive-compulsive disorder, and post-traumatic stress disorder in children and youths aged 6–24 years [39].

Encouragingly, the “Weifang Medical University-KIDSCREEN-Mandarin Chinese (WFMU-KS-MC)” research group, comprising most authors of this article, completed the cross-cultural translation of the self-reported KS-52 in 2016. Moreover, its Mandarin Chinese version’s psychometric properties have also been validated among adolescents in urban Weifang City, Shandong Province, the Chinese Mainland [40]. These initial findings on the original long KIDSCREEN version offer valuable insights into using the self-reported KIDSCREEN questionnaires in the Chinese Mainland. However, it is imperative to broaden the scope of research. Although the Mandarin Chinese self-reported KS-27 and KS-10, have some applications in the Chinese Mainland [41, 42], a lack of studies is still dedicated to comprehensively evaluating their psychometric properties [3]. This highlights a significant research gap, which negatively affects the promotion and utilization of both short KIDSCREEN versions in the Chinese Mainland. To comprehensively address this research gap, we published this journal article, providing detailed evidence and thereby promoting our outcomes. This initiative allows us to systematically and scientifically respond to demands for the concise nature of KIDSCREEN among Chinese Mainland researchers and practitioners.

To our best knowledge, no nationwide dataset in the Chinese Mainland specifically evaluates the psychometric properties of the Mandarin Chinese self-reported KS-27 and KS-10. Hence, this study reanalyzed the validation dataset of the Mandarin Chinese self-reported KS-52 conducted in Weifang City, the Chinese Mainland [40, 43]. This study aimed to simultaneously evaluate the psychometric properties of the Mandarin Chinese self-reported KS-27 and KS-10, and initially determine their suitability for measuring adolescents’ HRQoL in the Chinese Mainland, particularly in large-scale population surveys and clinical settings.

## Methods

### Participants

This study reanalyzed the validation dataset of the Mandarin Chinese self-reported KS-52. The dataset originated from a cross-sectional survey conducted in three urban areas (Kuiwen District, Weicheng District, and Hi-tech Industrial Development Zone) of Weifang City, the eastern part of the Chinese Mainland, from October to November 2016 [40].

This cross-sectional survey comprised two cohorts: baseline and retest (two weeks later). The baseline survey used a multistage design, stratified by region, school, and grade, and then randomly sampled schools and classrooms at the cluster level. Consequently, 4693 potential participants were included from 100 classes in 19 schools (primary, junior secondary, and senior secondary). Then, data from 4385 eligible participants meeting the baseline survey’s inclusion criteria were finally screened. The retest survey employed a convenient and clustered sampling strategy, resulting in 867 potential participants from 19 classes in 11 schools. Finally, data from 841 eligible participants meeting the unique inclusion criteria of retest surveys were identified. Complete and detailed information on the design and history of the study can be found elsewhere [40].

### Ethical considerations and informed consent

This study was conducted after the approval by the Medical Ethics Committee of Weifang Medical University. Initially, the principals and school doctors from all participating schools were contacted for the approval of participation and gave their permission. Next, each class adviser sent out and collected the parents’ informed consent through the parent conference or the WeChat group. Finally, the informed consent was also arranged on the first page of the questionnaire and in the teacher’s guide manual. Participation was voluntary, and each adolescent decided whether to fill in the questionnaire after reading the informed consent. It is worth noting that if adolescents want to quit after the study begins, they can raise their hands and stop answering without explaining why.

### Measures

Drawing on the original KIDSCREEN study [20, 37], the cross-sectional study was a comprehensive survey of adolescents that included a series of variables and questionnaires. This study mainly consisted of four parts: individual characteristics, e.g., age, gender, and ethnicity; family characteristics, including siblings, parents’ education level, and socioeconomic status (SES); HRQoL status; and mental health status (MHS).

#### KIDSCREEN questionnaires

The KIDSCREEN questionnaires measure the HRQoL of children and adolescents aged 8–18 years with three versions (KS-52, KS-27, and KS-10) for various research objectives and practical applications [20, 37]. They are available in self-reported (child/adolescent) and proxy-reported (parent/caregiver) informant forms, with this study using only the former. The time frame refers to the previous week. The vast majority of responses are categorized into a 5-point Likert scale that evaluates the frequency (1 = *never*, 2 = *seldom*, 3 = *quite often*, 4 = *very often*, 5 = *always*) or the intensity (1 = *not at all*, 2 = *slightly*, 3 = *moderately*, 4 = *very*, 5 = *extremely*) of certain behaviors, feelings, and attitudes. Only one item, measuring subjective health status, had significantly different responses (1 = *poor*, 2 = *fair*, 3 = *good*, 4 = *very good*, 5 = *excellent*). Negatively worded items are reverted, and item scores for each dimension (KS-52 and KS-27) or GHS (KS-10) are summed up. The raw scores for each dimension or GHS are transformed into *T*-scores with a mean (*M*) of 50 and a standard deviation (*SD*) of 10, based on European norms [20, 37]. Higher scores indicate better HRQoL. The complete and detailed information on the KIDSCREEN questionnaires can be found elsewhere [37].

The WFMU-KS-MC research group obtained permission from the European KIDSCREEN Group and signed an agreement to allow cross-cultural translation of the self-reported KS-52 and verify its psychometric properties in a Chinese Mainland adolescent population. The cross-cultural translation process consists of four modules: Mandarin Chinese Translation (Module I), Large-scale Expert Consultation (Module II), Small Group Pilot Survey (Module III), and Back Translation of the Final Version (Module IV):

- Mandarin Chinese Translation (Module I): In Module I, we used a “forward-backward-forward” methodology, following integrated methods and guidelines [44–49], consisting of six steps: Forward Translation (Step 1), Synthesis of the Forward Translation (Step 2), Backward Translation (Step 3), Synthesis of the Backward Translation (Step 4), Local Expert Committee Review (Step 5), and Repeat Steps 1–5 above (Step 6) until no disputes remained.
- Large-scale Expert Consultation (Module II): In Module II, we designed an expert consultation questionnaire based on the content validity index (CVI) [50, 51] and invited 13 experts from the Chinese Mainland to fill in. Based on the experts’ responses, we quantitatively evaluated the self-reported KS-52’s content validity, analyzing the relevance and representativeness of its items. In addition, we sought these experts’ comments on the accuracy, comprehensibility (readability), or any aspect of this version. Their insights and suggestions were carefully recorded and considered as part of our efforts to enhance this version’s appropriateness in the Chinese Mainland.
- Small Group Pilot Survey (Module III): In Module III, we conducted a pilot survey with a small target group of 48 school students (11–14 years old) using convenience sampling. The pilot survey included two stages: trial filling in the standardized questionnaire and filling in the comprehensibility (readability) questionnaire. Through both stages, we obtained information about this version from the target group’s feedback. This feedback includes—but is not limited to—completion time of fill in, comprehensibility (readability), and applicability (instruction, cover letter, items and responses, etc.). Then, the phrasing of some items was further minor adjusted, with no items substantially modified, added, or removed. Finally, the final version of the Mandarin Chinese self-reported KS-52 was formulated.
- Back Translation of the Final Version (Module IV): In Module IV, we organized the final back-translation following the KIDSCREEN Group Europe guidelines and completed a semantic consistency review between the final back-translated and the original English version [20]. Based on this review’s results, alongside findings from the first three modules, we decided to fully adopt the final version of the Mandarin Chinese self-reported KS-52. Subsequently, we reviewed and finalized the final version’s spelling, grammar, punctuation, and formatting.

Cross-cultural translation information for the Mandarin Chinese self-reported KS-52 is also available elsewhere [40].

#### KIDSCREEN-52 (KS-52)

The KS-52 (52 items, ten dimensions) is the original long version of the KIDSCREEN questionnaires, providing the most comprehensive and systematic insight into the children’s and adolescents’ HRQoL [20, 37]. Its excellent psychometric properties in cross-cultural settings have been demonstrated [21]. The Mandarin Chinese self-reported KS-52 was previously validated [40]. This study used it as the golden standard to evaluate the criterion validity of both short KIDSCREEN versions. Its internal consistency across the ten dimensions was appropriate in this study, with Cronbach’s alpha (α) coefficients of 0.819–0.959, and McDonald’s omega (ω) coefficients of 0.827–0.960.

#### KIDSCREEN-27 (KS-27)

The KS-27 is the shorter version of the KIDSCREEN questionnaires, derived from the original long KIDSCREEN version (KS-52), with 27 items across five dimensions. Various language versions validated its cross-cultural comparability and psychometric properties [37]. This study aimed to verify the psychometric properties of the Mandarin Chinese self-reported KS-27.

#### KIDSCREEN-10 index (KS-10)

The KS-10 is the shortest version of the KIDSCREEN questionnaires, comprising 10 items extracted from the KS-27 with the best psychometric properties, forming a one-dimensional measure. It provides a GHS representing longer versions’ dimensions. Various language versions validated its cross-cultural comparability and psychometric properties [30]. This study aimed to verify the psychometric properties of the Mandarin Chinese self-reported KS-10.

#### Pediatric Quality of Life Inventory*™* Version 4.0 Generic Core Scales (PedsQL*™* 4.0)

The PedsQL™ 4.0 is a generic HRQoL instrument for healthy and chronically ill children and adolescents aged 8–18 years [52]. It comprises 23 items across four dimensions: Physical Functioning (8 items), Emotional Functioning (5 items), Social Functioning (5 items), and School Functioning (5 items). Responses are categorized into a 5-point Likert scale that evaluates the frequency (0 = *never*, 1 = *seldom*, 2 = *sometimes*, 3 = *often*, 4 = *always*), with a one-month recall period. The dimension and total scores are converted into a 0–100-point scale, with 100 indicating the best HRQoL and 0 indicating the worst [53]. The Mandarin Chinese self-reported PedsQL™ 4.0 used in this study has been validated previously [54, 55]. The WFMU-KS-MC research group obtained permission from the cross-cultural developer to use this instrument in academic research [54]. Its internal consistency across the four dimensions was appropriate in this study, with Cronbach’s alpha (α) coefficients of 0.810–0.907, and McDonald’s omega (ω) coefficients of 0.818–0.908.

#### Family Affluence Scale II (FAS II)

The FAS II measures family SES, particularly in children’s and adolescents’ health research. It measures family SES indirectly through a family wealth indicator, including family car ownership, having own unshared room, the number of computers at home, and how many times the family went on holidays in the past 12 months [56]. The raw total scores can be classified into eight categories of 0–7, and recoded into three categories (Low 0–3, Medium 4–5, and High 6–7) [57, 58]. Its global cross-cultural validity is established, and a validated Mandarin Chinese version exists [57, 58]. Its internal consistency met acceptable standards in this study, with Cronbach’s alpha (α) coefficient of 0.591 and McDonald’s omega (ω) coefficient of 0.671.

#### Strengths and Difficulties Questionnaire (SDQ)

The SDQ is a brief screening instrument to measure behavioral difficulties, capturing the MHS of children and adolescents aged 4–16 years [59]. It comprises 25 items across five dimensions: Emotional Symptoms, Conduct Problems, Hyperactivity/Inattention, Peer Problems, and Prosocial Behavior, with five items in each dimension. Responses are categorized into a 3-point scale (0 = *not true*, 1 = *somewhat true*, 2 = *certainly true*), with a six-month recall period. A few items are reverse-scored. After recoding reverse-scored items, the Total Difficulties Score (TDS) is obtained by summing up scores from all 20 items except those in the Prosocial Behavior. The possible range of TDS is 0–40, with cutoff values from the Shanghai norms classifying it as “Normal” (0–14), “Borderline” (15–17), and “Abnormal” (18–40) [60]. The Mandarin Chinese self-reported SDQ used in this study has been validated previously [60, 61], and it was excerpted from the “Handbook of Commonly Used Psychological Assessment Scales” published in the Chinese Mainland [62]. Its internal consistency of the TDS was appropriate, with Cronbach’s alpha (α) coefficient of 0.936 and McDonald’s omega (ω) coefficient of 0.943.

### Statistical analysis

#### Data analysis and reporting framework

To ensure methodological rigor in statistical analysis and result presentation, this study incorporated the COnsensus-based Standards for the selection of health status Measurement INstruments (COSMIN) [63]. This consensus checklist of measurement properties was developed through an international Delphi study based on various standards, especially the patient-reported outcome measures (PROMs) [64]. Due to the clarity and operability of its definition, terminology, and framework, COSMIN is widely applied across various fields, beyond just health-related patient reporting outcomes (HR-PRO) [63–65]. Detailed descriptions of COSMIN can be found elsewhere [63–66]. In summary, combined with COSMIN, the KIDSCREEN handbook, and specific research requirements, this study has formed a data analysis and reporting framework, including feasibility, item and dimension properties, reliability, and validity.

#### Feasibility

Consistent with previous studies [67–69], this study evaluated the feasibility of the Mandarin Chinese self-reported KS-27 and KS-10 regarding completion time and response rate. For completion time, data from 48 adolescents (aged 11–14 years) during the trial filling in the standardized questionnaire stage were analyzed. Although data were collected for the original long KIDSCREEN version (KS-52), both short KIDSCREEN versions are expected to require less time. This study used this indicator to indirectly evaluate the approximate completion time of both short KIDSCREEN versions.

This study clearly defined the response rate in the field survey and data cleaning stages. The response occurs at both unit- and item-level [70]. At the unit-level, the focus is on the survey’s overall response rate or completion rate. It represents the proportion of individuals or households participating in the survey out of the total number of eligible units (such as persons or households) in the target population. The unit-level response rate indicates the overall participation or engagement in the survey. At the item-level, the focus is on the response rate for individual survey items or questions. It represents the proportion of respondents who provide a valid response to a specific item or question out of the total number of respondents who were asked that particular item. The item-level response rate provides insights into the completeness of data for each specific item or question in the survey. The unit-level response rate is useful for evaluating the overall representativeness and generalizability of the survey findings. The item-level response rate is important for assessing the quality and completeness of data for individual survey items or questions, and it is also reflected as missing values. This study calculated the unit response rate using the method outlined in the “Standard Definitions: Final Dispositions of Case Codes and Outcome Rates for Surveys” of the American Association for Public Opinion Research (AAPOR) [71], supported by detailed field records of both surveys. Moreover, the item response rate, which is the proportion of missing values that can be indirectly measured, was evaluated at the item and dimension levels. This study considered < 3% overall missing data acceptable [63], although consensus on the issue varies, with recommended criterion values ranging from < 5% to < 20% [72, 73].

#### Item and dimension properties

The psychometric properties of item and dimension were measured using the following indicators. First, the count and frequency/percentage of all items’ responses described the distribution of each category. Second, the raw scores of items and dimensions (including GHS) were reported using *M* and *SD* [63]. Besides, following the KIDSCREEN Group Europe’s scoring algorithm [20, 37], the raw scores of dimensions (including GHS) were linked to the Rasch scores weighted by European norms and ultimately reported as *T*-scores (*M* = 50, *SD* = 10). This study used these *T*-scores for known-group validity analysis. Third, “Cronbach’s alpha (α) and McDonald’s omega (ω) if item deleted” were also calculated; if both indicators change little after an item is deleted, the item can be retained [63, 74, 75]. Fourth, the floor and ceiling effects of all dimensions (including GHS) were calculated [63, 74]. If more than 15% of respondents achieved the lowest or highest possible scores on a given dimension (including GHS), they are defined as having floor or ceiling effects [63, 76].

#### Reliability

Referring to COSMIN, reliability was evaluated through internal consistency and test–retest reliability. Internal consistency measures the degree of the inter-relatedness among items [64], evaluated using inter-item and item-total correlations, Cronbach’s alpha (α), Guttman’s lambda-2 and -6 (λ_2_ and λ_6_), and McDonald’s omega (ω). Inter-item and item-total correlations indicate whether an item belongs to the instrument. This study adopted the standard for inter-item correlation between items in the same dimension (including GHS) at 0.20–0.50 [63], and the standard for item-total correlation of the same dimension (including GHS) at ≥ 0.30 [77]. Cronbach’s α is widely used for internal consistency, with a reasonable range of 0.80–0.90 [74, 78]. Despite its popularity and widespread application, it has faced criticism [79, 80]. Therefore, this study also reported three alternative coefficients: Guttman’s lambda-coefficients (λ_2_ and λ_6_) and McDonald’s omega (ω). λ_2_ and λ_6_ are more credible indicators [74, 81], with recommended values > 0.70 [82]. Besides, ω provides an optimum estimation of homogeneity reliability [83], with a value > 0.70 indicating a reliable total score [84].

Test–retest reliability (score consistency over time) was evaluated using the intraclass correlation coefficient (ICC) at a two-week interval, specifically adopting the ICC (two-way mixed-effects model, single measure, and absolute agreement type) [85–87]. An ICC value > 0.70 indicates excellence [25, 74, 76, 88]. Besides, absolute reliability was evaluated using the standard error of measurement (*SEM*), calculated via repeated analysis of variance (ANOVA) [89, 90], to quantify measurement errors and estimate measurement precision [89].

#### Validity

Validity evaluation is essential when introducing a health measurement instrument. Following the COSMIN and specific research requirements, this study evaluated construct validity (structural validity and hypothesis testing) and criterion validity [63].

Structural validity was evaluated using confirmatory factor analysis (CFA) and multi-group confirmatory factor analysis (MG-CFA). Drawing from prior empirical work on the KS-27 and KS-10 [20, 30, 37], this study tentatively assumed the five-dimensional structure (KS-27) and the one-dimensional structure (KS-10). CFA was employed to evaluate both datasets (baseline and retest surveys) to check the above factor structures’ robustness better. Maximum likelihood estimator (MLE) was used to fit both first-order CFA models, with latent variables defined using the fixed loading method (fixing the first path of each latent variable to 1). Besides, for the KS-27, the interrelationship between the five latent variables was assumed to be correlated. The overall model fit was evaluated using chi-square (χ^2^) test and normed chi-square (χ^2^/*df*). Traditionally, a nonsignificant χ^2^ value has been used to prove a good model-data fit. However, this value is sensitive to sample size and non-normality (both tend to cause the χ^2^ to expand) [91]. Furthermore, in this case, the χ^2^/*df* index also loses its value, although the reference value indicates that χ^2^/*df* < 2 is a good model, and when < 3 is acceptable [92]. Therefore, this study also considered various fitting indicators to evaluate model-data fitting [93, 94]. The comparative fit index (CFI) and Tucker-Lewis index (TLI), with values > 0.90 or > 0.95, indicate acceptable or good model fit, respectively [95–97]. Similarly, the standardized root-mean-square residual (SRMR) and root-mean-square error of approximation (RMSEA), with values < 0.05 or 0.08, indicate good or acceptable fit, respectively [97–99]. Additionally, power analysis for both models was conducted based on RMSEA and noncentral chi-square distributions for tests of three different null hypotheses [92, 100, 101].

The MG-CFA method was used to evaluate measurement invariance across groups (gender and grade) in the five-dimensional structure (KS-27) and the one-dimensional structure (KS-10), respectively. This study employed a four-step approach to establish measurement invariance models: configural invariance, metric invariance, scalar invariance, and strict invariance [102, 103]. These four measurement invariance models were nested, allowing the next model only when the former was established. Goodness-of-fit between the nested measurement invariance models was compared using differences in CFI, TLI, SRMR, and RMSEA, with thresholds of ΔCFI ≤ 0.01, ΔTLI ≤ 0.01, ΔSRMR ≤ 0.03, and ΔRMSEA ≤ 0.015 [104–106]. The chi-square difference test was not used due to its dependence on sample size with a rejection of models with trivial practical misfits in large samples (*N* > 300) [105, 107, 108].

The basic principle of construct validation is that hypotheses are formulated about the relationships of scores on the instrument under study with scores on other instruments measuring similar or dissimilar constructs, or differences in the instrument scores between subgroups of participants [63]. Based on this conceptual model or theories about the construct, hypotheses can be formulated concerning expected relationships with instruments measuring related constructs. This study used two methods for hypothesis testing. The first method focused on convergent and discriminant validation, selecting the Mandarin Chinese self-reported PedsQL™ 4.0 for comparison, based on the KIDSCREEN handbook and previous studies in the Chinese Mainland [20, 54, 55]. Specifically, convergent and discriminant validity were evaluated by determining the degree of correlation between two instruments measuring similar or less comparable dimensions of HRQoL. Convergent validity indicated high correlations between dimensions reflecting the same underlying concept (e.g., KS-27’s Psychological Well-being and PedsQL™ 4.0’s Emotional Functioning), while discriminant validity indicated lower correlations between dimensions measuring different characteristics (e.g., KS-27’s School Environment and PedsQL™ 4.0’s Physical Functioning). Pearson correlation measured the strength of this association, with values traditionally categorized as trivial (< 0.10), low (0.10–0.30), moderate (0.31–0.50), and high (≥ 0.50) [76]. However, in empirical studies, many researchers recommend a cutoff value of ≥ 0.45 [109].

The second method focused on known-group validity, verifying discriminative validity by comparing the instrument scores’ differences between subgroups. The known-group validity of both short KIDSCREEN versions was examined by comparing the results between groups, which were priori expected to show differences in HRQoL. This study conducted a series of one-way ANOVAs to test the significant differences between SES (low/medium/high) and MHS (abnormal/borderline/normal) concerning all dimensions (KS-27) and GHS (KS-10). This study further adopted the effect analysis (η^2^) corresponding to the ANOVA. In addition, based on the original KIDSCREEN study [20, 37], this study hypothesized that higher SES would correlate with higher scores in all dimensions (KS-27) and GHS (KS-10), particularly in Autonomy and Parent Relation. Similarly, higher MHS scores were expected to be associated with lower scores in all dimensions (KS-27) and GHS (KS-10), especially in Psychological Well-being. After that, this study then compared extreme groups (e.g., low SES vs. high SES) to test these hypotheses, using independent sample *t*-tests to determine statistically significant differences and Cohen’s *d* to measure effect sizes. Following the cutoff values outlined [110], this study interpreted effect size magnitudes of η^2^ (0.01 as small, 0.06 as moderate, and 0.14 as large) and Cohen’s *d* (0.20 as small, 0.50 as moderate, and 0.80 as large), respectively.

The COSMIN panel defines criterion validity as “the degree to which the scores of a measurement instrument are an adequate reflection of a golden standard” [64]. This study used the Mandarin Chinese self-reported KS-52 as the golden standard because it has existed and proven to have excellent psychometric properties [40]. Criterion validity was evaluated akin to convergent validity, with both dimensions (e.g., Psychological Well-being of both KS-52 and KS-27), expected to show a high correlation. The intraclass correlation coefficient (ICC) was used, specifically the ICC (two-way mixed-effects model, single measure, and consistency type) [63, 85, 86], with an ICC value > 0.70 generally considered acceptable [25, 76, 88, 109].

#### Missing values

Before conducting the above analysis, this study analyzed the missing values of both surveys (baseline vs. retest). At the item level, although each item had missing values, the percentage of missing values was extremely low. In the baseline survey, the highest number of missing values came from item 19, reaching 26 (0.6%), while in the retest survey, the number became 4 (0.5%) from item 24. However, the percentage of missing values was slightly higher at the dimension level but acceptable. In both surveys, the highest number of missing in all dimensions (KS-27) and GHS (KS-10) came from the latter, reaching 94 (2.1%) and 21 (2.5%), respectively. Overall, in the baseline survey, all KS-27 items were completed in 4114 participants (93.8%) without any missing values; in the KS-10, the number was higher, reaching 4291 (97.9%). The retest survey was similar to the baseline; all items of KS-27 were completed in 789 participants (93.8%); in the KS-10, the number was 820 (97.5%). The detailed results for the missing values above are shown in Table 1. Combined with the above situation, this study adopted the deletion method to deal with these negligible missing values.

**Table 1:** Description, item and dimension properties, and reliability coefficients of the KIDSCREEN-27 and KIDSCREEN-10 index

**Item and dimension labels**	***N***^**a**^	**MV**^**a**^ **(%)**	**Distribution of responders (%) over the response options**					***M* (*SD*)**	**If the corresponding item deleted**		**Item-rest correlation**	***N***^**b**^	**MV**^**b**^ **(%)**	***N***_**pairs**_	**ICC [95% CI]**	***SEM***
			**1**	**2**	**3**	**4**	**5**		**α**	**ω**						
**Physical Well-being (KS-27)**	4301	1.9	—	—	—	—	—	**18.21 (2.39)**	—	—	—	837	0.5	812	0.800 [0.763, 0.828]	0.949
1. In general, how would you say health is?	4363	0.5	0.1	3.3	5.9	69.1	21.7	4.09 (0.64)	0.824	0.826	0.667	840	0.1	831	0.658 [0.618, 0.695]	0.364
2. *Have you felt fit and well?*	4367	0.4	0.0	1.1	19.4	66.3	13.2	3.92 (0.60)	0.819 (0.842)	0.820 (0.848)	0.690 (0.430)	840	0.1	837	0.663 [0.623, 0.699]	0.352
3. Have you been physically active?	4367	0.4	0.0	2.0	26.5	55.2	16.2	3.85 (0.70)	0.825	0.825	0.669	840	0.1	834	0.663 [0.623, 0.699]	0.383
4. Have you been able to run well?	4370	0.3	0.0	1.1	11.6	57.0	30.3	4.17 (0.66)	0.823	0.823	0.671	840	0.1	835	0.645 [0.604, 0.683]	0.367
5. *Have you felt full of energy?*	4370	0.3	0.0	1.3	13.3	62.8	22.6	4.07 (0.64)	0.830 (0.843)	0.831 (0.849)	0.645 (0.422)	840	0.1	838	0.661 [0.621, 0.697]	0.370
**Psychological Well-being (KS-27)**	4333	1.2	—	—	—	—	—	**27.04 (4.65)**	—	—	—	829	1.4	818	0.825 [0.802, 0.846]	1.709
6. Has your life been enjoyable?	4377	0.2	0.3	3.4	30.4	51.2	14.6	3.77 (0.75)	0.866	0.876	0.658	840	0.1	838	0.677 [0.639, 0.712]	0.394
7. Have you been in a good mood?	4376	0.2	0.2	3.1	25.8	50.1	20.7	3.88 (0.77)	0.866	0.878	0.647	840	0.1	836	0.696 [0.659, 0.729]	0.439
8. Have you had fun?	4376	0.2	0.2	2.9	21.8	49.5	25.6	3.98 (0.78)	0.869	0.880	0.624	840	0.1	840	0.666 [0.627, 0.702]	0.433
9. *Have you felt sad?*	4377	0.2	0.4	5.8	32.4	31.8	29.6	3.84 (0.93)	0.852 (0.826)	0.842 (0.833)	0.753 (0.617)	839	0.2	837	0.659 [0.618, 0.696]	0.531
10. Felt so bad that didn’t want to do anything?	4379	0.1	0.6	7.9	28.7	24.8	38.0	3.92 (1.01)	0.856	0.849	0.729	839	0.2	839	0.650 [0.607, 0.689]	0.573
11. *Have you felt lonely?*	4377	0.2	0.9	7.0	24.9	32.0	35.2	3.94 (0.98)	0.854 (0.824)	0.845 (0.831)	0.735 (0.630)	838	0.4	835	0.608 [0.563, 0.649]	0.555
12. Have you been happy with way you are?	4379	0.1	0.6	9.5	23.7	53.3	12.9	3.68 (0.84)	0.879	0.885	0.539	838	0.4	838	0.637 [0.595, 0.675]	0.547
**Autonomy and Parent Relation (KS-27)**	4304	1.8	—	—	—	—	—	**25.78 (4.37)**	—	—	—	822	2.3	806	0.810 [0.780, 0.836]	1.770
13. *Have you had enough time for yourself?*	4377	0.2	1.0	11.9	36.6	25.9	24.5	3.61 (1.01)	0.752 (0.828)	0.720 (0.837)	0.542 (0.596)	838	0.4	836	0.590 [0.512, 0.655]	0.635
14. *Able to do things—want to do in free time?*	4379	0.1	1.1	10.4	30.6	28.9	29.0	3.75 (1.02)	0.750 (0.830)	0.718 (0.839)	0.550 (0.584)	838	0.4	836	0.656 [0.591, 0.709]	0.607
15. Parent(s) had enough time for you?	4378	0.2	0.1	6.3	31.3	41.8	20.4	3.76 (0.85)	0.758	0.715	0.515	838	0.4	837	0.687 [0.649, 0.721]	0.457
16. *Have your parent(s) treated you fairly?*	4378	0.2	0.4	2.4	14.2	51.2	31.8	4.12 (0.76)	0.755 (0.840)	0.709 (0.846)	0.548 (0.457)	839	0.2	838	0.635 [0.593, 0.674]	0.446
17. Able to talk to parent(s) when wanted to?	4378	0.2	0.4	5.8	28.7	43.7	21.3	3.80 (0.86)	0.748	0.709	0.570	838	0.4	835	0.661 [0.621, 0.697]	0.494
18. Enough money to do things as friends?	4363	0.5	2.5	25.5	40.4	14.4	17.2	3.18 (1.08)	0.777	0.755	0.432	838	0.4	835	0.669 [0.629, 0.704]	0.606
19. Had enough money for your expenses?	4359	0.6	0.8	14.1	37.6	26.4	21.1	3.53 (1.00)	0.767	0.742	0.467	839	0.2	835	0.680 [0.642, 0.715]	0.515
**Social Support and Peers (KS-27)**	4353	0.7	—	—	—	—	—	**15.80 (2.45)**	—	—	—	830	1.3	826	0.785 [0.757, 0.810]	1.056
20. Have you spent time with your friends?	4378	0.2	0.3	6.9	43.4	39.6	9.8	3.52 (0.78)	0.870	0.870	0.675	838	0.4	838	0.674 [0.635, 0.709]	0.441
21. *Have you had fun with your friends?*	4376	0.2	0.0	1.7	15.7	58.0	24.5	4.05 (0.69)	0.824 (0.836)	0.824 (0.843)	0.786 (0.515)	839	0.2	838	0.617 [0.573, 0.658]	0.432
22. You and your friends helped each other?	4376	0.2	0.0	1.5	13.3	58.2	27.1	4.11 (0.67)	0.825	0.825	0.785	838	0.4	836	0.671 [0.632, 0.707]	0.383
23. Have you been able to rely on your friends?	4377	0.2	0.0	3.2	11.7	55.1	29.9	4.12 (0.73)	0.853	0.854	0.710	838	0.4	837	0.630 [0.588, 0.669]	0.429
**School Environment (KS-27)**	4357	0.6	—	—	—	—	—	**14.33 (2.61)**	—	—	—	829	1.4	825	0.813 [0.788, 0.835]	1.135
24. Have you been happy at school?	4380	0.1	0.7	3.8	35.7	51.4	8.4	3.63 (0.72)	0.856	0.857	0.700	837	0.5	836	0.691 [0.654, 0.725]	0.434
25. *Have you got on well at school?*	4376	0.2	1.7	11.0	53.7	25.2	8.4	3.27 (0.83)	0.848 (0.827)	0.849 (0.836)	0.728 (0.604)	838	0.4	837	0.693 [0.656, 0.727]	0.462
26. *Have you been able to pay attention?*	4377	0.2	0.2	6.4	32.2	52.0	9.2	3.64 (0.75)	0.828 (0.827)	0.830 (0.837)	0.773 (0.618)	838	0.4	837	0.681 [0.643, 0.716]	0.481
27. Got along well with your teachers?	4378	0.2	0.3	4.5	26.2	54.5	14.5	3.78 (0.75)	0.838	0.842	0.747	838	0.4	837	0.647 [0.605, 0.685]	0.474
**Global HRQoL Score (KS-10)**	4291	2.1	—	—	—	—	—	**38.24 (5.40)**	—	—	—	820	2.5	803	0.842 [0.816, 0.864]	1.949

*Note*. *KS-27* KIDSCREEN-27, *KS-10* KIDSCREEN-10 index, *MV* missing value, *α* Cronbach’s alpha, *ω* McDonald’s omega, *N*_pairs_ pairs of responders matched by the test–retest survey, *ICC* intra-class correlation coefficient, *CI* confidence interval, *SEM* standard error of measurement

KS-27 included all 27 items; KS-10 included items 2, 5, 9, 11, 13, 14, 16, 21, 25, and 26 (indicated in *italics*). In the column “*M* (*SD*),” KS-27’s dimension scores and KS-10’s Global HRQoL Score are indicated in **bold**. Two indicators of “α” and “ω” of the KS-27’s dimensions and KS-10’s Global HRQoL Score are indicated under the “If the corresponding item deleted” column. It is considering the three indicators of “α,” “ω,” and “Item-rest correlation,” the items that belong to both the KS-27 and KS-10 correspond to two values. The listwise (casewise) deletion method is used to deal with missing values

^a^the baseline survey, ^b^ the retest survey

All correlation coefficients are statistically significant at *p* < 0.001 level

#### Data distribution

The distribution of raw data is a prerequisite because it directly involves selecting subsequent analysis methods. This study checked the distribution characteristics of raw data from two surveys using various techniques. If the following standards are met, it is proven that the raw data is normally distributed or approximately normally distributed: (1) the skewness and kurtosis value (item- and dimension-level) of the raw data should be between ± 1.0 (excellent) or ± 2.0 (acceptable) [111]; (2) the *z*-value of the skewness and kurtosis (*z*-value_skewness_ = skewness value/standard error of skewness; *z*-value_kurtosis_ = kurtosis value/standard error of kurtosis) should be somewhere in the span of − 1.96 to + 1.96 [112]; (3) the *p*-value of two tests (Lilliefors corrected Kolmogorov-Smirnov and Shapiro-Wilk) should be greater than 0.05 [113]; (4) the histograms, box plots, probability-probability (P-P) plots, and quantile-quantile (Q-Q) plots should visually indicate that data are approximately normally distributed [114, 115].

#### Statistical software

In the process of data input, Epiadta3.1 (EpiData Association, Odense, Denmark) was used to establish the database. IBM SPSS Statistics (Version 28.0; SPSS Inc., Armonk, NY, USA) was used for data management and most of the statistical analysis. Specifically, it included all descriptive analysis (such as social demographic, the count and frequency/percentage of all items’ responses, and raw item and dimension score, etc.); nonparametric test calculations (Mann-Whitney *U* test and Kruskal-Wallis *H* test) for completion time; test–retest reliability analysis; the majority of validity analysis (hypothesis testing and criterion validity); and the effect size calculations (${{\upeta }}_{H}^{2}$, η^2^, Cohen’s *d*). The majority of internal consistency analysis (item-total correlation, Cronbach’s α, Guttman’s λ_2_ and λ_6_, McDonald’s ω) were performed using the JASP (Version 0.18.3; JASP Team, University of Amsterdam, Amsterdam, Netherlands). Regarding structural validity, including CFA and MG-CFA, SPSS Amos 28.0 software was used. Besides, for the power estimates of both CFA models, the code was generated using a web utility (http://www.quantpsy.org/rmsea/rmsea.htm) [116] and implemented with R (Version 4.2.0, R Development Core Team, April, 2022). The statistical significance of all analyses was *p* < 0.05.

## Results

### Socio-demographic of the sample

A sample of 4385 adolescents participated in baseline and 841 in retest surveys. In both surveys, the average age of adolescents was 13.71 and 13.81 years, ranging from 11 to 17 years. The gender ratio of males and females was roughly the same (46.9%, 53.1% vs. 48.5%, 51.5%). The overwhelming majority of adolescents (99.1%, 99.5%) were of the Han ethnicity, and slightly less than half (48.7%, 47.8%) came from the one-child family. Most parents’ education level was secondary school, reaching (65.3%, 68.4%) and (68.2%, 69.8%), respectively. Besides, 41.5% and 34.8% of adolescents were from families with high SES.

### Data distribution characteristics

The distribution characteristics of the raw data from both surveys were checked at the item- and dimension-level. Skewness and kurtosis values mostly fell within ± 1.0, indicating approximate normality. Visual inspections of histograms, box plots, P-P plots, and Q-Q plots further supported the approximately normal distribution. However, most *z*-values for the skewness and kurtosis did not fall between − 1.96 and + 1.96. In addition, both statistical tests of data distribution confirmed significant departures from normality, with all the *p*-values less than 0.05 (detailed results are given in Table A of the Supplementary Materials). Both pieces of evidence mentioned later show that these raw data were skewed distribution. Although this may appear contradictory, there is no cause for concern. Many studies have shown that the latter two pieces of evidence are easy to lose effectiveness in the case of large samples [117–119]. Therefore, this study referenced the skewness, kurtosis values, and graphical evidence to determine that these data were approximately normal. In fact, in empirical studies, for most statistical tests, it is sufficient that the data are approximately normally distributed [117–119].

### Feasibility

#### Completion time

In the Small Group Pilot Survey, 48 adolescents (26 males vs. 22 females, aged 11–14 years, grades 6th–9th) took 12.33 mins on average to complete the original long KIDSCREEN version (KS-52) (*Mdn* = 12.00, *IQR* = 12–13, Min = 11, and Max = 14). Therefore, it is inevitable that the completion time for both short KIDSCREEN versions will be shorter. The Mann-Whitney *U* test revealed no significant difference in completion time between gender groups (*Mdn*_(male)_ = 12.50, *Mdn*_(female)_ = 12.00; *U* = 261.000, *z* = − 0.543, *p* = 0.587). However, Kruskal-Wallis *H* tests indicated the significant differences in completion times across age groups (*Mdn*_(11)_ = 13.00, *Mdn*_(12)_ = 13.00, *Mdn*_(13)_ = 12.00, *Mdn*_(14)_ = 12.00; *H*_(*df* = 3)_ = 17.622, *p* < 0.001, ${ {\upeta }}_{H}^{2}$ = 0.332) and grade groups (*Mdn*_(6th)_ = 13.00, *Mdn*_(7th)_ = 13.00, *Mdn*_(8th)_ = 12.00, *Mdn*_(9th)_ = 11.50, *H*_(*df* = 3)_ = 17.900, *p* < 0.001, ${ {\upeta }}_{H}^{2}$ = 0.339). Older and higher-grade adolescents had shorter completion times than younger and lower-grade adolescents.

#### Response rate

Response rates were high in both surveys (baseline and retest), with unit response rates of 93.4% (4385/4693) and 97.0% (841/867), respectively. Item response rates, indirectly obtained through missing value proportions, exceeded 99.0% for all items of both short KIDSCREEN versions. At the dimension level, response rates for all dimensions (including GHS) also exceeded 97.0%. Details are shown in Table 1.

### Item and dimension properties

The 27 items were rated on a Likert scale (1–5), ranging from “*poor*” or “*not at all*” or “*never*” (category 1) to “*excellent*” or “*extremely*” or “*always*” (category 5). Table 1 shows the distribution of respondents across response categories. Fewer adolescents chose “category 1” (0.0–2.5%); thus, combining “category 1” and “category 2” might be an option when scoring. Besides, Table 1 displays the *M* and *SD* of items and dimensions (including GHS). Next, as shown in Table 1, deleting either item in the belonged dimension (including GHS) had a small negative effect on respective Cronbach’s α and McDonald’s ω. Finally, in all dimensions (including GHS), the floor effect is almost 0, while the ceiling effects ranged from 0.5% (GHS) to 8.8% (Social Support and Peers).

### Reliability

#### Internal consistency

The inter-item correlations ranged from 0.173 to 0.873 for all the dimensions (KS-27) and GHS (KS-10). Details are shown in Table B.1–B.6 of the Supplementary Materials. In addition, Table 1 lists the corrected item-domain correlations of all 27 items (KS-27) and all 10 items (KS-10), with values ranging from 0.422 to 0.786, reflecting satisfactory scale homogeneity. Finally, Cronbach’s α, Guttman’s (λ_2_ and λ_6_), and McDonald’s ω for all the dimensions (including GHS) exceeded 0.80, except for the Autonomy and Parent Relation, where α and ω were slightly lower. Details are shown in Table 2.

**Table 2:** Internal consistency for the KIDSCREEN-27 and KIDSCREEN-10 index

	**Internal consistency**			
	**α [95% CI]**	**λ**_**2**_ **[95% CI]**	**λ**_**6**_ **[95% CI]**	**ω** **[95% CI]**
**KIDSCREEN-27 **				
Physical Well-being	0.854 [0.847, 0.861]	0.855 [0.845, 0.863]	0.829 [0.818, 0.839]	0.855 [0.848, 0.862]
Psychological Well-being	0.881 [0.875, 0.886]	0.889 [0.884, 0.894]	0.896 [0.892, 0.901]	0.886 [0.881, 0.892]
Autonomy and Parent Relation	0.786 [0.775, 0.796]	0.807 [0.799, 0.815]	0.875 [0.869, 0.881]	0.725 [0.713, 0.737]
Social Support and Peers	0.877 [0.871, 0.883]	0.878 [0.871, 0.883]	0.853 [0.846, 0.860]	0.877 [0.871, 0.883]
School Environment	0.877 [0.871, 0.883]	0.878 [0.872, 0.884]	0.846 [0.838, 0.854]	0.879 [0.873, 0.885]
**KIDSCREEN-10 index **				
Global HRQoL Score	0.847 [0.840, 0.853]	0.860 [0.854, 0.865]	0.880 [0.875, 0.885]	0.853 [0.847, 0.860]

*Note*. *α* Cronbach’s alpha, *λ*_2_ Guttman’s lambda-2, *λ*_6_ Guttman’s lambda-6, *ω* McDonald’s omega, *CI* confidence interval

The listwise (casewise) deletion method is used to deal with missing values

#### Test–retest reliability

This study found good consistency over time for all the dimensions (KS-27) and GHS (KS-10), as indicated by ICCs [95% CI] at 0.785 [0.757, 0.810]–0.842 [0.816, 0.864]. For individual items, the ICCs were somewhat low at 0.590 [0.512, 0.655]–0.696 [0.659, 0.729] but reached an acceptable level. Furthermore, the relatively small *SEM* of items (0.352–0.635), as well as all the dimensions and GHS (0.949–1.949), further supported absolute reliability. Details are shown in Table 1.

### Structural validity

#### Confirmatory factor analysis

This study initially evaluated the hypothetical five- and one-dimensional structures using CFA with the complete dataset from the baseline survey. However, all the goodness-of-fit indicators for both initial models were unacceptable. Specifically, the five-dimensional structure had inadequate fit (χ^2^_(*df*)_ = 15156.493_(314)_, *p* < 0.001, χ^2^/*df* = 48.269, CFI = 0.778, TLI = 0.752, SRMR = 0.075, RMSEA [90% CI] = 0.107 [0.106, 0.109]), while the one-dimensional structure exhibited even worse fit (χ^2^_(*df*)_ = 7759.748_(35)_, *p* < 0.001, χ^2^/*df* = 221.707, CFI = 0.601, TLI = 0.487, SRMR = 0.102, RMSEA [90% CI] = 0.227 [0.223, 0.231]). We found unambiguous evidence of misspecification in both models when examining modification indices (MIs). For the five-dimensional structure, a significant MI (MI = 3100.159) pointed to the pairing of error terms between items 13 and 14 (err_13_↔err_14_). Moreover, the Parameter Change value was 0.641, indicating that if the analysis is repeated with the covariance between err_13_ and err_14_ as a free parameter, its estimate will increase by approximately 0.641. However, the modified model requires real-world evidence to avoid falling into the data-driven abyss [120]. In other words, the additional covariant terms need to be meaningful in the concept and the real world. Thus, this study re-examined both items (item 13, “Have you had enough time for yourself?”; item 14, “Have you been able to do the things that you want to do in your free time?”). Both items are closely related in the literal meaning, concerning individuals’ opportunities for social and leisure time, originally belonging to the Autonomy dimension of the original long KIDSCREEN version (KS-52) [20, 37]. Using this initial evidence, this study set the residuals of both items as free estimates (correlated residuals) and reanalyzed them to obtain a new model (M_2_ in Table 3). The unstandardized estimate for this error covariance parameter was 0.691, highly significant (critical ratio (CR) = 39.084), and even larger than the earlier parameter change statistics predicted. The standardized parameter estimate was 0.839, reflecting a powerful error correlation. The chi-square difference test (Δχ^2^) was performed, and both models were assumed to be nested. The comparison of M_2_ (χ^2^_(313)_ = 10392.121) with M_1_ (χ^2^_(314)_ = 15156.493), yielded a significant difference in χ^2^ value (Δχ^2^_(1)_ = 4764.372, *p* < 0.001). Furthermore, other goodness-of-fit indicators, such as CFI and TLI, both tended to increase to 1; while SRMR and RMSEA changed small and tended to 0. All the above indicators showed that the modified model outperformed the initial one. Similar methods were applied between item 18 (“Have you had enough money to do the same things as your friends?”) and item 19 (“Have you had enough money for your expenses?”) on the Autonomy and Parent Relation, followed by that between item 6 (“Has your life been enjoyable?”), item 7 (“Have you been in a good mood?”) and item 8 (“Have you had fun?”) on the Psychological Well-being. The final modification model’s goodness-of-fit statistics were also calculated (a visual depiction of the final revised model is available in Figure A.1 of the Supplementary Materials). The χ^2^_(309)_ = 3948.857 (*p* < 0.001) and χ^2^/*df* = 12.779, which was beyond the acceptable range. However, other indicators were ideal (CFI = 0.946, TLI = 0.938, SRMR = 0.089, and RMSEA [90% CI] = 0.054 [0.052, 0.055]). Specific goodness-of-fit indicators and modification steps for the KS-27 are presented in Table 3, and the parameter estimates are available on the left side of Table C.1 of the Supplementary Materials.

**Table 3:** Specific goodness-of-fit indicators and modification steps of CFA in the KIDSCREEN-27 (*N* = 4114)

**Model**	**Model goodness-of-fit indicators**					
	**χ**^**2**^**(*df*)**	**χ**^**2**^**/*df***	**CFI**	**TLI**	**SRMR**	**RMSEA [90% CI]**
M_1_ (initial)	15156.493^***^ (314)	48.269	0.778	0.752	0.075	0.107 [0.106, 0.109]
M_2_ (err_13_↔err_14_)	10392.121^***^ (313)	33.202	0.849	0.831	0.081	0.088 [0.087, 0.090]
M_3_ (err_18_↔err_19_)	7113.772^***^ (312)	22.801	0.898	0.886	0.081	0.073 [0.071, 0.074]
M_4_ (err_7_↔err_8_)	5819.721^***^ (311)	18.713	0.918	0.907	0.086	0.066 [0.064, 0.067]
M_5_ (err_6_↔err_7_)	5274.128^***^ (310)	17.013	0.926	0.916	0.086	0.062 [0.061, 0.064]
M_6_ (err_6_↔err_8_)	3948.857^***^ (309)	12.779	0.946	0.938	0.089	0.054 [0.052, 0.055]
Cutoff value	N/A	< 3	> 0.90	> 0.90	≤ 0.08	≤ 0.08

*Note*. *CFA* confirmatory factor analysis, *CFI* comparative fit index, *TLI* Tucker-Lewis index, *SRMR* standardized root-mean-square residual, *RMSEA* root-mean-square error of approximation, *CI* confidence interval, *err* error, *N/A* not applicable. The listwise (casewise) deletion method is used to deal with missing values

^***^p < 0.001

For the one-dimensional structure, three modifications were made following the same strategy as above (a visual depiction of the final revised model is available in Figure A.2 of the Supplementary Materials). The first modification addressed items 13 and 14; the second modification was item 9 (“Have you felt sad?”) and item11 (“Have you felt lonely?”); the third modification was item 2 (“Have you felt fit and well?”) and item 5 (“Have you felt full of energy?”). Finally, ideal results were obtained (CFI = 0.970, TLI = 0.958, SRMR = 0.040, and RMSEA [90% CI] = 0.065 [0.060, 0.069]). Specific goodness-of-fit indicators and modification steps for the KS-10 are presented in Table 4, and the parameter estimates are available on the left side of Table C.2 of the Supplementary Materials. To further prove the robustness of both final models, this study used retest data and fit following the same strategy as described above. Ideal results were obtained for both the five-dimensional structure (χ^2^_(309)_ = 483.231, *p* < 0.001, χ^2^/*df* = 1.564, CFI = 0.962, TLI = 0.956, SRMR = 0.038, and RMSEA [90% CI] = 0.027 [0.022, 0.031]) and the one-dimensional structure (χ^2^_(32)_ = 36.212, *p* = 0.278, χ^2^/*df* = 1.132, CFI = 0.996, TLI = 0.995, SRMR = 0.021, and RMSEA [90% CI] = 0.013 [0.000, 0.030]). Specific goodness-of-fit indicators and parameter estimates are shown in Table C and Table D of the Supplementary Materials, respectively.

**Table 4:** Specific goodness-of-fit indicators and modification steps of CFA in the KIDSCREEN-10 index (*N *= 4291)

**Model**	**Model goodness-of-fit indicators**					
	**χ**^**2**^**(*df*)**	**χ**^**2**^**/*df***	**CFI**	**TLI**	**SRMR**	**RMSEA [90% CI]**
M_1_ (initial)	7759.748^***^ (35)	221.707	0.601	0.487	0.102	0.227 [0.223, 0.231]
M_2_ (err_13_↔err_14_)	2949.468^***^ (34)	86.749	0.849	0.801	0.067	0.141 [0.137, 0.146]
M_3_ (err_9_↔err_11_)	1492.601^***^ (33)	45.230	0.925	0.897	0.058	0.102 [0.097, 0.106]
M_4_ (err_2_↔err_5_)	610.346^***^ (32)	19.073	0.970	0.958	0.040	0.065 [0.060, 0.069]
Cutoff value	N/A	< 3	> 0.90	> 0.90	≤ 0.08	≤ 0.08

*Note*. *CFA* confirmatory factor analysis, *CFI* comparative fit index, *TLI* Tucker-Lewis index, *SRMR* standardized root-mean-square residual, *RMSEA* root-mean-square error of approximation, *CI* confidence interval, *err* error, *N/A* not applicable. The listwise (casewise) deletion method is used to deal with missing values

^***^p < 0.001

Finally, the power analysis was conducted on both models in different datasets (baseline vs. retest), and four results obtained were infinitely close to 1 (> 0.998 or 0.999). Therefore, this indicates high confidence that both models will not lead to Type II error.

#### Multi-group confirmatory factor analysis

Results regarding the measurement invariance of both models across subgroups (gender and grade groups) are presented in Tables 5 and 6. For gender groups (male/female), the results across four steps ranging from least to most rigorous suggested invariance of the five-dimensional structure (ΔCFI = 0.000, 0.005, and 0.000 ≤ 0.01; ΔTLI = 0.002, 0.003, and 0.003 ≤ 0.01; ΔSRMR = 0.003, 0.001, and 0.001 ≤ 0.03; ΔRMSEA = 0.001, 0.001, and 0.001 ≤ 0.015) and the one-dimensional structure (ΔCFI = 0.000, 0.008, and 0.000 ≤ 0.01; ΔTLI = 0.005, 0.005, and 0.004 ≤ 0.01; ΔSRMR = 0.002, 0.000, and 0.000 ≤ 0.03; ΔRMSEA = 0.003, 0.003, and 0.002 ≤ 0.015). Thus, considering gender, both models achieved full measurement invariance. However, they failed to achieve full measurement invariance considering the grade groups roughly divided by the school (primary/junior secondary/senior secondary). While configural and metric invariance were accepted (ΔCFI = 0.004 vs. 0.006 ≤ 0.01; ΔTLI = 0.001 vs. 0.001 ≤ 0.01; ΔRMSEA = 0.000 vs. 0.000 ≤ 0.015), the subsequent scalar invariance was rejected due to significant differences in fit indices (ΔCFI = 0.034 vs. 0.053 > 0.01; ΔTLI = 0.032 vs. 0.046 > 0.01). Specific goodness-of-fit indicators (Table D) and the item covariance matrices of all samples and subgroups from both surveys (Table E–H) can be obtained from the Supplementary Materials.

**Table 5:** Measurement invariance of the KIDSCREEN-27 across gender and grade groups

**Model**	**Model goodness-of-fit indicators**												
	**χ**^**2**^**(*df*)**	**χ**^**2**^**/*df***	**CFI**	**TLI**	**SRMR**	**RMSEA [90% CI]**	**Nested models**	**∆χ**^**2**^**(∆*df*)**	**∆CFI**	**∆TLI**	**∆SRMR**	**∆RMSEA**	**Decision**
Gender group^a^													
M_1_: Configural invariance	4303.455^***^ (618)	6.964	0.945	0.937	0.089	0.038 [0.037, 0.039]	—	—	—	—	—	—	—
M_2_: Metric invariance	4344.008^***^ (640)	6.788	0.945	0.939	0.092	0.038 [0.037, 0.039]	M_2_ vs. M_1_	40.553^**^ (22)	0.000	0.002	0.003	0.001	Accept
M_3_: Scalar invariance	4685.961^***^ (667)	7.025	0.940	0.937	0.093	0.038 [0.037, 0.039]	M_3_ vs. M_2_	341.953^***^ (27)	0.005	0.003	0.001	0.001	Accept
M_4_: Strict invariance	4738.603^***^ (694)	6.828	0.940	0.939	0.092	0.038 [0.037, 0.039]	M_4_ vs. M_3_	52.642^**^ (27)	0.000	0.003	0.001	0.001	Accept
Grade group^b^													
M_1_: Configural invariance	4573.988^***^ (927)	4.934	0.944	0.936	0.066	0.031 [0.030, 0.032]	—	—	—	—	—	—	—
M_2_: Metric invariance	4870.462^***^ (971)	5.016	0.940	0.935	0.115	0.031 [0.030, 0.032]	M_2_ vs. M_1_	296.474^***^ (44)	0.004	0.001	0.049	0.000	Accept
M_3_: Scalar invariance	7155.438^***^ (1025)	6.981	0.905	0.903	0.113	0.038 [0.037, 0.039]	M_3_ vs. M_2_	2284.976^***^ (54)	0.034	0.032	0.001	0.007	Reject
Cutoff value	N/A	< 3	≥ 0.90	≥ 0.90	≤ 0.08	≤ 0.08	N/A	N/A	≤ 0.01	≤ 0.01	≤ 0.03	≤ 0.015	N/A

*Note*. *CFI* comparative fit index, *TLI* Tucker-Lewis index, *SRMR* standardized root-mean-square residual, *RMSEA* root-mean-square error of approximation, *CI* confidence interval, *N/A* not applicable. The listwise (casewise) deletion method is used to deal with missing values

^a^*N*_(Full sample)_ = 4114, *n*_1(Male sample)_ = 1921, *n*_2(Female sample)_ = 2193; ^b ^*N*_(Full sample)_ = 4114, *n*_1(Primary school sample)_ = 912, *n*_2(Junior secondary school sample)_ = 2022, *n*_3(Senior secondary school sample)_ = 1180

^**^p < 0.01. ^*** ^*p* < 0.001

**Table 6:** Measurement invariance of the KIDSCREEN-10 index across gender and grade groups

**Model**	**Model goodness-of-fit indicators**												
	**χ**^**2**^**(*df*)**	**χ**^**2**^**/*df***	**CFI**	**TLI**	**SRMR**	**RMSEA [90% CI]**	**Nested models**	**∆χ**^**2**^**(∆*df*)**	**∆CFI**	**∆TLI**	**∆SRMR**	**∆RMSEA**	**Decision**
Gender group^a^													
M_1_: Configural invariance	628.810^***^(64)	9.825	0.971	0.959	0.042	0.045 [0.042, 0.049]	—	—	—	—	—	—	—
M_2_: Metric invariance	646.147^***^ (73)	8.851	0.970	0.963	0.040	0.043 [0.040, 0.046]	M_2_ vs. M_1_	17.337^c^ (9)	0.000	0.005	0.002	0.003	Accept
M_3_: Scalar invariance	819.072^***^ (83)	9.868	0.962	0.959	0.040	0.046 [0.043, 0.048]	M_3_ vs. M_2_	172.925^***^ (10)	0.008	0.005	0.000	0.003	Accept
M_4_: Strict invariance	833.063^***^ (93)	8.958	0.962	0.963	0.040	0.043 [0.040, 0.046]	M_4_ vs. M_3_	13.991^d^ (10)	0.000	0.004	0.000	0.002	Accept
Grade group^b^													
M_1_: Configural invariance	754.914^***^ (96)	7.864	0.964	0.949	0.046	0.040 [0.037, 0.043]	—	—	—	—	—	—	—
M_2_: Metric invariance	855.879^***^ (114)	7.771	0.957	0.949	0.066	0.040 [0.037, 0.042]	M_2_ vs. M_1_	130.965^***^ (18)	0.006	0.001	0.021	0.000	Accept
M_3_: Scalar invariance	1868.800^***^ (134)	13.946	0.904	0.903	0.115	0.055 [0.053, 0.057]	M_3_ vs. M_2_	982.920^***^ (20)	0.053	0.046	0.049	0.015	Reject
Cutoff value	N/A	< 3	≥ 0.90	≥ 0.90	≤ 0.08	≤ 0.08	N/A	N/A	≤ 0.01	≤ 0.01	≤ 0.03	≤ 0.015	N/A

*Note*. *CFI* comparative fit index, *TLI* Tucker-Lewis index, *SRMR* standardized root-mean-square residual, *RMSEA* root-mean-square error of approximation, *CI* confidence interval, *N/A* not applicable. The listwise (casewise) deletion method is used to deal with missing values

^a^*N*_(Full sample)_ = 4291, *n*_1(Male sample)_ = 2011, *n*_2(Female sample)_ = 2280; ^b ^*N*_(Full sample)_ = 4291, *n*_1(Primary school sample)_ = 947, *n*_2(Junior secondary school sample)_ = 2110, *n*_3(Senior secondary school sample)_ = 1234

^***^p < 0.001. ^c ^*p* = 0.044. ^d ^*p* = 0.173

### Hypothesis testing

#### Convergent and discriminant validity

Convergent and discriminant validity were evaluated by determining the degree of correlation from KS-27 or KS-10 with those of PedsQL™ 4.0, measuring similar or less comparable HRQoL dimensions. The correlations between five dimensions (KS-27) or GHS (KS-10) and PedsQL™ 4.0’s dimensions are presented in Table 7. The KS-27’s dimension scores were positively and significantly correlated with the PedsQL™ 4.0’s dimension scores. As expected, the correlations of both dimensions dealing with similar structures displayed a high level of correlation, suggesting excellent convergent validity. For example, the PedsQL™ 4.0’s Emotional Functioning showed the highest correlation with the KS-27’s Psychological Well-being (*r* = 0.851), followed by that between School Functioning and School Environment (*r* = 0.771). Also, Emotional Functioning and School Functioning (PedsQL™ 4.0) had the highest correlation with GHS (KS-10), at 0.779 and 0.726, respectively. Conversely, the correlations between less comparable dimensions were low (e.g., 0.340, 0.388, 0.356 between Autonomy and Parent Relation, Social Support and Peers, and School Environment (KS-27) and Physical Functioning (PedsQL™ 4.0)), confirming discriminant validity.

**Table 7:** Convergent and discriminant validity for the KIDSCREEN-27 and KIDSCREEN-10 index

	**PedsQL™ 4.0**			
	**Physical Functioning**	**Emotional Functioning**	**Social Functioning**	**School Functioning**
**KIDSCREEN-27**				
Physical Well-being	0.705^**^ (4232)	0.395^**^ (4242)	0.365^**^ (4238)	0.456^**^ (4251)
Psychological Well-being	0.432^**^ (4263)	0.851^**^ (4276)	0.598^**^ (4269)	0.623^**^ (4283)
Autonomy and Parent Relation	0.340^**^ (4231)	0.564^**^ (4244)	0.492^**^ (4241)	0.530^**^ (4256)
Social Support and Peers	0.388^**^ (4281)	0.513^**^ (4293)	0.720^**^ (4288)	0.553^**^ (4303)
School Environment	0.356^**^ (4284)	0.589^**^ (4297)	0.556^**^ (4292)	0.771^**^ (4307)
**KIDSCREEN-10 index**				
Global HRQoL Score	0.494^**^ (4221)	0.779^**^ (4235)	0.640^**^ (4230)	0.726^**^ (4241)

*Note*. *PedsQL™ 4.0* Pediatric Quality of Life Inventory™ Version 4.0 Generic Core Scales. The pairwise deletion method is used to deal with missing values. All Pearson correlation coefficients; Figures in brackets indicate paired cases; Values in **bold** showed the highest correlations of each column

^**^p < 0.01

#### Known-group validity

Table 8 presents gradients for all dimensions (KS-27) and GHS (KS-10) across the family SES. One-way ANOVAs revealed significant effects of SES on all dimensions and GHS (*F* = 93.414–443.240, *p*_s_ < 0.001). Also, the last two columns of Table 8 show two standardized effect sizes (η^2^ and Cohen’s *d*). A large η^2^ = 0.17 was found in Autonomy and Parent Relation between these three groups, and η^2^ in the Psychological Well-being, Social Support and Peers, School Environment, and GHS were medium (0.07–0.09). The η^2^ of the remaining Physical Well-being was small at 0.04. Regarding Cohen’s *d* between two SES groups (low vs. high), GHS and Autonomy and Parent Relation were large (0.81 and 1.03), while medium Cohen’s *d* was in the other dimensions (0.55–0.72).

**Table 8:** Differences in the KIDSCREEN-27 and KIDSCREEN-10 index by socioeconomic status

	**SES**				
	**Low**^**a**^	**Medium**^**b**^	**High**^**c**^	**Effect size**	
	***T*-score** ***M* (*SD*)**	***T*-score** ***M* (*SD*)**	***T*-score** ***M* (*SD*)**	**η**^**2**^	**Cohen’s** ***d*** **(Low vs. High)**
**KIDSCREEN-27**					
Physical Well-being	48.96 (7.19)	50.64 (7.86)	53.21 (8.12)	0.04	**0.55**
Psychological Well-being	43.24 (7.90)	45.40 (8.40)	49.58 (9.24)	**0.08**	**0.72**
Autonomy and Parent Relation	42.08 (4.96)	44.26 (5.55)	49.36 (7.82)	**0.17**	**1.03**
Social Support and Peers	44.85 (6.61)	46.65 (7.30)	50.09 (8.24)	**0.07**	**0.67**
School Environment	44.23 (6.10)	46.14 (7.57)	50.06 (8.88)	**0.08**	**0.72**
**KIDSCREEN-10 index**					
Global HRQoL Score	43.89 (6.08)	46.07 (7.36)	50.09 (8.30)	**0.09**	**0.81**

*Note*. This study used the Family Affluence Scale (FAS) to indirectly assess SES. *SES* socioeconomic status. The listwise (casewise) deletion method is used to deal with missing values

^a^*n* = 791, ^b ^*n* = 1775, ^c ^*n* = 1819

All mean differences are statistically at *p* < 0.001 level

η^2^ 0.01 = small, 0.06 = medium, 0.14 = large; Cohen’s *d *0.20 = small, 0.50 = medium, 0.80 = large

The effect size of “medium” or greater is indicated in **bold**

Table 9 presents gradients for all dimensions (KS-27) and GHS (KS-10) across the MHS. One-way ANOVAs revealed significant effects of MHS on all dimensions and GHS (*F* = 206.897–1359.821, *p*_s_ < 0.001). The η^2^ in the Physical Well-being was medium at 0.09, while the remaining were large (0.20–0.40). Regarding Cohen’s *d* between two MHS groups (abnormal vs. normal), Cohen’s *d* was large (1.15–1.83) in all dimensions and GHS except that Physical Well-being was medium at 0.73.

**Table 9:** Differences in the KIDSCREEN-27 and KIDSCREEN-10 index by mental health status

	**MHS**				
	**Abnormal**^**a**^	**Borderline**^**b**^	**Normal**^**c**^	**Effect size**	
	***T*-score** ***M* (*SD*)**	***T*-score** ***M* (*SD*)**	***T*-score** ***M* (*SD*)**	**η**^**2**^	**Cohen’s** ***d*** **(Abnormal vs. Normal)**
**KIDSCREEN-27**					
Physical Well-being	47.40 (7.50)	48.66 (6.30)	53.03 (7.87)	**0.09**	**0.73**
Psychological Well-being	37.36 (4.53)	40.34 (4.47)	50.53 (7.85)	**0.40**	**1.83**
Autonomy and Parent Relation	40.63 (4.93)	42.74 (4.65)	48.14 (6.98)	**0.20**	**1.15**
Social Support and Peers	41.16 (6.53)	46.54 (6.14)	50.04 (7.16)	**0.22**	**1.27**
School Environment	40.41 (4.99)	43.60 (4.85)	50.14 (7.92)	**0.25**	**1.33**
**KIDSCREEN-10 index**					
Global HRQoL Score	39.20 (3.88)	42.32 (3.39)	50.55 (7.11)	**0.38**	**1.75**

*Note*. This study used the Total Difficulties Score (TDS) of the Strengths and Difficulties Questionnaire (SDQ) to indirectly assess MHS. *MHS* mental health status. The listwise (casewise) deletion method is used to deal with missing values

^a^*n* = 921, ^b ^*n* = 347, ^c ^*n* = 2872

All mean differences are statistically at *p* < 0.001 level

η^2 ^0.01 = small, 0.06 = medium, 0.14 = large; Cohen’s *d* 0.20 = small, 0.50 = medium, 0.80 = large

The effect size of “medium” or greater is indicated in **bold**

### Criterion validity

The Mandarin Chinese self-reported KS-52 served as the criterion. Significant and positive correlations were observed in the correlation matrix between its ten dimensions and the five dimensions (KS-27) and GHS (KS-10), as shown in Table 10. As expected, ICCs between dimensions with similar structures, exhibited strong correlations, highlighting the reasonably robust criterion validity. For example, the highest correlations came from the School Environment of the KS-52 and KS-27 (ICC = 0.903), followed by Social Support and Peers of the KS-52 and KS-27 (ICC = 0.897), KS-52’s Mood and Emotions and KS-27’s Psychological Well-being (ICC = 0.869). Regarding the correlations between KS-52 and KS-10, Mood and Emotions had the highest relationship with GHS (ICC = 0.783), followed by School Environment and GHS (ICC = 0.740), Autonomy and GHS (ICC = 0.727).

**Table 10:** Criterion validity for the KIDSCREEN-27 and KIDSCREEN-10 index

	**KIDSCREEN-27**					**KIDSCREEN-10 index**
	**Physical Well-being**	**Psychological Well-being**	**Autonomy and Parent Relation**	**Social Support and Peers**	**School Environment**	**Global HRQoL Score**
**KIDSCREEN-52**						
Physical Well-being	1.000^***^	0.376^***^	0.330^***^	0.382^***^	0.395^***^	0.431^***^
Psychological Well-being	0.374^***^	0.814^***^	0.562^***^	0.439^***^	0.485^***^	0.625^***^
Moods and Emotions	0.267^***^	0.869^***^	0.518^***^	0.333^***^	0.418^***^	0.783^***^
Self-perception	0.352^***^	0.631^***^	0.475^***^	0.406^***^	0.493^***^	0.552^***^
Autonomy	0.230^***^	0.480^***^	0.710^***^	0.309^***^	0.367^***^	0.727^***^
Parent Relation and Home Life	0.270^***^	0.555^***^	0.760^***^	0.414^***^	0.447^***^	0.600^***^
Financial Resources	0.270^***^	0.330^***^	0.612^***^	0.383^***^	0.401^***^	0.350^***^
Social Support and Peers	0.357^***^	0.537^***^	0.535^***^	0.897^***^	0.542^***^	0.616^***^
School Environment	0.348^***^	0.625^***^	0.587^***^	0.516^***^	0.903^***^	0.740^***^
Social Acceptance (Bullying)	0.317^***^	0.347^***^	0.316^***^	0.554^***^	0.494^***^	0.345^***^

*Note*. All Intraclass correlation coefficient (ICC); Values in **bold **showed the highest correlations of each column; Because the items for the Physical Well-being of KIDSCREEN-52 and KIDSCREEN-27 remain unchanged, the ICC value is 1.000. The listwise (casewise) deletion method is used to deal with missing values

^***^p < 0.001

## Discussion

Measuring HRQoL in children and adolescents is crucial for healthcare treatment and preventive programs [121], demanding reliable and valid instruments. The self-reported KIDSCREEN questionnaires are the generic HRQoL instruments suitable for screening, monitoring, and evaluation [20, 37], with three versions (KS-52, KS-27, and KS-10) adaptable to various settings. The previous study validated the psychometric properties of the Mandarin Chinese self-reported KS-52 [40]. In contrast, both short KIDSCREEN versions can improve the measurement of HRQoL by saving response time and effort, increasing the response rate, minimizing burden, and decreasing fatigue effects [20, 30, 37, 39, 122]. Nevertheless, to our best knowledge, no study is available to date to evaluate their psychometric properties simultaneously in the Mandarin Chinese context. This study filled this gap, demonstrating that the Mandarin Chinese self-reported KS-27 and KS-10 had excellent feasibility, item and dimension properties, reliability, and validity.

The Mandarin Chinese self-reported KS-27 and KS-10 are highly feasible and acceptable for measuring adolescents’ HRQoL, with easy completion, less time requirements, high response rates, and few missing values. In the Small Group Pilot Survey, only the approximate completion time of the original long KIDSCREEN version (KS-52) was roughly calculated. Since both short KIDSCREEN versions contain fewer items, it is reasonable to expect they will take less time. HRQoL measurement instruments typically have no time limit, unlike intelligence and academic performance tests [20]. However, considering children’s and adolescents’ limited concentration time, completion time was also a significant feasibility indicator in this study [67, 123, 124]. Compared to the original KIDSCREEN study, which recommended completion times of 10–15 and 5 mins for both short KIDSCREEN versions, respectively [20, 37], this study meets the standard and is even slightly better. Besides, this study’s school-based sampling and management surveys produced higher response rates and fewer missing values, consistent with previous international studies [30, 125, 126]. Overall, the fact mentioned above indicates that both short KIDSCREEN versions are viable in the Mandarin Chinese context.

Excellent psychometric properties were demonstrated in all items and dimensions of the Mandarin Chinese self-reported KS-27 and KS-10. The negative effects of deleting either item on the respective Cronbach’s α and McDonald’s ω were negligible for all the dimensions (KS-27) and GHS (KS-10). Besides, there were almost negligible floor and no significant ceiling effects across all the dimensions (KS-27) and GHS (KS-10). These results were similar to those in international studies [20, 37]. Compared to the original KIDSCREEN study, both Mandarin Chinese KIDSCREEN versions actually reduced the ceiling effect on some dimensions [20, 37]. It may be that this study targeted general adolescent samples, where higher ceiling effects would be expected [40, 127]. Thus, further testing of both Mandarin Chinese KIDSCREEN versions is required in clinical samples, where the ceiling effect would likely be significantly reduced.

The internal consistency of the Mandarin Chinese self-reported KS-27 and KS-10 was appropriate, similar to the original KIDSCREEN study [20, 37]. Most dimensions showed satisfactory inter-item correlations except for very few items of the Autonomy and Parent Relation (KS-27) and GHS (KS-10). Besides, all corrected item-total correlations fell within an acceptable range, reflecting satisfactory scale homogeneity. Cronbach’s α values exceeded 0.80 for almost all dimensions (KS-27) and GHS (KS-10), consistent with findings from several cross-cultural studies [38, 128, 129]. Other internal consistency indicators, like Guttman’s λ_2_ and λ_6_, and McDonald’s ω, offer a comprehensive and informed reliability evaluation [81, 130]. These indicators all met the standard for excellent internal consistency. Notably, test–retest correlations (ICCs) were above 0.75 and close to 0.90, respectively, indicating good and excellent reliability [87]. In this study, all dimensions (including GHS) had test–retest ICCs that were slightly exceeding the threshold of 0.70 [76], and also surpassed the original KIDSCREEN study’s range of 0.55–0.74 [20, 37]. However, the ICC is a dimensionless statistic that may overestimated with large between-subject variability and underestimated with small between-subject variability [131]. In contrast, *SEM* relies solely on within-subject variation [89]. This study found that the *SEM* values were very low, indicating excellent absolute reliability. Overall, both Mandarin Chinese KIDSCREEN versions demonstrated high internal consistency and reproducibility, indicating strong reliability.

This study initially supported the five- and one-dimensional structure of the Mandarin Chinese self-reported KS-27 and KS-10, with results consistent with other recent European, Asian, and African studies [30, 129, 132–138]. Both final models of this study achieved excellent overall fitting results after significant modification indices were considered. These additional covariant terms are meaningful in the concept and the real world. For the five-dimensional structure (KS-27), items 13 and 14 on the Autonomy and Parent Relation originally belonged to the Autonomy dimension of the original long KIDSCREEN version (KS-52). Both items are related to opportunities for social and leisure time, and are highly consistent in semantics. Similarly, items 18 and 19 on the Autonomy and Parental Relationship are related to the perceived family economics, and both items with highly similar semantics originally come from the Financial Resources of the original long KIDSCREEN version (KS-52). Several international studies have confirmed the high relevance of both items [38, 139–141]. In Norwegian and Brazilian samples, the items showed a high correlation, leading researchers to adopt a similar approach to this study [139, 140]. However, studies in Ireland, the UK, and the Hong Kong Special Administrative Region (SAR) of China took a different approach and identified a seven-dimensional structure that aligned with the data [38, 141]. In their studies, items 18 and 19 formed a separate dimension named “Financial Resources.” Regarding Items 6, 7, and 8 on Psychological Well-being are phrased positively (such as item 6, “Has your life been enjoyable?”), while the rest of the items 9, 10, and 11 are phrased negatively (such as item 9, “Have you felt sad?”). The former three items are initially derived from the Psychological Well-being of the original long KIDSCREEN version (KS-52), with all items in this dimension positive wording [20]. Conversely, the latter items originate from another dimension (Mood and Emotions) of the original long KIDSCREEN version (KS-52). Therefore, the three items 6, 7, and 8 are similar in meaning, so children and adolescents can select the same response without thorough consideration. In other words, possibly due to the item formulation’s consistency, potentially hindering their ability to discern the content adequately [140].

Items 13 and 14 of the KS-10 are prioritized for revision, followed by items 9 and 11, which have highly similar semantics originally from the Mood and Emotions of the original long KIDSCREEN version (KS-52), all negatively worded. There is strong evidence of a high correlation between these two negatively worded items [38, 139–141]. Previous studies have shown that respondents, especially younger children, may struggle to accurately report their perceptions using negatively worded items [142, 143]. This might be why these items were deemed to fall into distinct factors. The last revision is between items 2 and 5, originally from the Physical Well-being of the original long KIDSCREEN version (KS-52). Both items are related to the level of the children’s and adolescents’ physical activity, energy and fitness, and are highly consistent in semantics [20]. In short, this study modified the model based on real-world evidence rather than being kidnapped by data. The anomalies in these items have also been mentioned in previous studies [38, 139–141], but unfortunately, no clear explanation has been given.

This study attempts to provide an explanation grounded in existing data and theories, which could be attributed to two primary factors. The first factor pertains to the items with high correlation that exhibit a certain level of semantic similarity. The Mandarin Chinese could potentially amplify this similarity, leading students to choose the same response upon encountering semantically similar items, often without deliberate consideration [140]. The second factor is potentially more significant. Similar to the previous study in the Hong Kong SAR of China [38], this study also used the original long KIDSCREEN version (KS-52) for a large-scale empirical survey. Consequently, this study used this 52-item dataset to fit the 27-and 10-item questionnaires. What needs to be noted here is that the original long KIDSCREEN version (KS-52) has a fixed template, which displays the ten dimensions in understandable language in advance and has been restricted by bars. For example, open the third page (the first and second pages are the cover and instructions, respectively), and you’ll notice that the first dimension (Physical Well-being) is denoted as “Physical Activities and Health” to enhance comprehension for children and adolescent participants. Additionally, the dimension’s name is rendered in a larger and bold font size to enhance visibility. Following this, you’ll find a clearly defined recall period, five items, and their corresponding response options. The above details can be found in the KIDSCREEN handbook [20]. These formats and arrangements significantly enhance participants’ user experience (fostering a sense of ease and user-friendliness) [124]. This also embodies questionnaire standardization, consistent with the overarching emphasis on standardization and internationalization in the original KIDSCREEN study [20, 37]. Nevertheless, this fixed template might inadvertently solidify participants’ thought processes so that the participants can help fit the original long KIDSCREEN version (KS-52) in their subconscious. Therefore, this need for modification arises when this study used this 52-item dataset to fit both short KIDSCREEN versions of mislaid items. It should be noted that the above is a tentative explanation based on the actual situation of this study; there is not enough information to verify this explanation. Moreover, this study could not determine whether such differences were due to language issues, differences in cultural adaptations, or other possible factors. These will be examined in a future study.

The modified model and data fit very well; all indicators were excellent except for χ^2^ and χ^2^/*df*. A significant χ^2^ was observed, which might indicate a misfit of the five- and one-dimensional structure. However, this is more likely because this statistic value alone is not appropriate in large datasets [144], and this situation has also appeared in previous studies [20, 129, 145]. To further verify both modified models’ robustness, this study tested them in the retest samples (small samples). Ideal results were obtained, especially in the one-dimensional structure; the χ^2^ and χ^2^/*df* indexes were all up to the standard; in the five-dimensional structure, only the χ^2^/*df* index was up to the standard. This can explain, to a certain extent, why both modified models were robust. In other words, the initial χ^2^ and χ^2^/*df* indices did not match, which has a significant probability that the sample size is too large. The subsequent power analysis also strengthened this conclusion.

Additionally, this study supported the full configure and metric invariance of the five- and one-dimensional structure of gender and grade groups. However, at a more stringent level (scalar invariance), the grade group could not be considered fully invariant, indicating that complete strong equivalence was not met. Therefore, the Mandarin Chinese self-reported KS-27 and KS-10 may have systematic response bias in different grade groups; perhaps the measurement results may reflect real differences between grade groups [146]. To our best knowledge, this study is the first to test the measurement invariance of both short KIDSCREEN versions across gender and grade groups at a more stringent level, and the cause of this result is not yet clear. Future use of both short KIDSCREEN versions with adolescents should test the measurement invariance [136]. If necessary, a partial scalar invariance test can be carried out in the grade group [146–148], or a differential item functioning (DIF) test (more microscopic and meticulous) under the framework of item response theory (IRT) [149–151].

The convergent and discriminant validity analysis indicated that the Mandarin Chinese self-reported KS-27 and KS-10 showed a reasonable pattern of correlations. This study analyzed the validity by correlating the KS-27 and KS-10 with a similar instrument (PedsQL™ 4.0) to measure HRQoL. Correlations were generally highest for dimension pairs with expected high correlations; this suggests reasonable convergent validity. For example, the highest correlation was found between KS-27’s Psychological Well-being and PedsQL™ 4.0’s Emotional Functioning. Meanwhile, low to moderate correlations between theoretically different dimensions supported the construct validity in the form of discriminant validity. For example, the lowest correlation was found between the KS-27’s School Environment and PedsQL™ 4.0’s Physical Functioning. Previous research also used PedsQL™ 4.0 to verify the convergent and discriminant validity of both short KIDSCREEN versions and achieved excellent results [20, 126, 152].

There were significant differences in all dimensions (including GHS) of the Mandarin Chinese self-reported KS-27 and KS-10, according to different SES, especially in high vs. low. Although other previous studies have generally shown that the KIDSCREEN questionnaires can discriminate adolescents with different SES [20, 30, 37, 132, 137, 153], this study showed a greater magnitude of the effect sizes than the original KIDSCREEN study. The sample selection of this study, which was only for adolescents (lack of child samples), may be one reason because a previous study has shown that SES might be more critical for HRQoL in adolescents than in children [154]. Indeed, one would expect a comparison of both short KIDSCREEN versions between children and adolescents, but unfortunately, it was beyond the scope of this study. Furthermore, this study found that both short KIDSCREEN versions could discriminate between MHS of adolescents, consistent with the previous research in European samples [20, 37]. However, the effect sizes of this study on many dimensions were larger, especially between adolescents with poor vs. good MHS. The fantastic variation in the magnitude of effect sizes has been reported in previous studies [30, 132, 153, 154]. It might be because many KIDSCREEN dimensions focus more on “Mental and Social Health.” Besides, this study only surveyed adolescents aged 11–17 years who lack children’s groups, which may exacerbate the effect sizes, as the physical and mental in adolescents were more changeable and inequal [155]. Further research should confirm these results using both short KIDSCREEN versions in clinical settings.

HRQoL is an essential part of the PROMs, which often focus on subjective perceptions and opinions and almost always lack a golden standard [63]. An exception is when we want to develop a shorter questionnaire for a construct when a long version already exists [63]. This study used the Mandarin Chinese self-reported KS-52 (original long KIDSCREEN version) as the golden standard because it has existed and proven excellent psychometric properties [40]. As anticipated, the ICCs between two dimensions, which deal with similar structures, exhibited greater strength, underscoring the reasonably robust criterion validity. Overall, the criterion validity of the Mandarin Chinese self-reported KS-27 and KS-10 was more satisfactory, similar to the previous studies [30, 128, 132].

### Research limitations

These analyses were based on a pre-existing dataset of the Mandarin Chinese self-reported KS-52 validation study conducted in Weifang City, the Chinese Mainland, from October to November 2016. Some of the previous limitations have been described elsewhere [40]. However, this was a new analysis, so further limitations should exist.

The first limitation is about the sample’s age, although it was explained in a previous study [40]. However, further elaboration and clarification are necessary here. Samples aged 8–10 years were not included throughout the survey stage (pilot survey and large-scale empirical survey). Undeniably, this is indeed a significant limitation. Due to this limitation, it is impossible to know how children in this age group performed on these instruments. For example, many questions could not be answered during the pilot survey phase: “Can 8–10 children understand the items and responses?” “Do they need more time to complete than older adolescents?” and “What is the time gap?”. Addressing these uncertainties is crucial for the broader application of the Mandarin Chinese self-reported KIDSCREEN questionnaires in this age group of children. Therefore, researchers are encouraged to consider this in future studies.

The second limitation is the completion time for the Mandarin Chinese self-reported KS-27 and KS-10. Measuring the completion time accurately is nearly impossible in large-scale traditional paper-and-pencil surveys. Therefore, we only recorded the completion time of the Mandarin Chinese self-reported KS-52 in the pilot survey. Although it is possible to evaluate the duration of both short KIDSCREEN versions indirectly, it is not direct evidence. Also, referring to the first limitation, the lack of 8–10-year-old samples in the pilot survey made evaluating this age group’s completion time impossible. Therefore, other researchers are encouraged to study the completion time of both short KIDSCREEN versions. Moreover, it is not impossible to record the completion time of the large-scale survey. The computer-administered tests could accurately and efficiently record the completion time [156], undoubtedly a promising research direction.

The third limitation is that this study used the previous dataset of the original long KIDSCREEN version (KS-52) to evaluate the psychometric properties of both short KIDSCREEN versions. As is known, items in both short KIDSCREEN versions were embedded in the original long KIDSCREEN version [20, 37]. Therefore, it remains to be seen whether this study’s application was responsible for changes in the psychometric properties (e.g., modifications of both factor structures) compared to the separate application of both short KIDSCREEN versions. Importantly, the potential impact of this study’s application on the scores of both short KIDSCREEN versions remains unclear. Thus, using both short KIDSCREEN versions alone in future research may be an excellent way to answer these two uncertainties. Additionally, it is essential to note that using the same responses from participants to evaluate criterion validity for both short and long KIDSCREEN versions of the instrument may introduce biases in interpreting the results. Hence, caution is warranted when interpreting the criterion validity results of this study.

The fourth limitation is that this study was based on the classical test theory (CTT) and did not consider the IRT analysis under the modern measurement theory (MMT) framework. Due to the inherent limitations of the CTT framework, some problems could not be explained clearly. For example, in the measurement invariance (grade group) of both short KIDSCREEN versions, the results of MG-CFA were not ideal, and the best strategy is to do further DIF analysis based on the IRT framework [149–151]. However, this is beyond the scope of this study; our future research will consider adopting the IRT framework.

The fifth limitation concerns the calculation of *T*-scores for both short KIDSCREEN versions. This study utilized European norms to derive *T*-scores, rather than norms from the Chinese Mainland adolescent population. The reason for this was that the Chinese Mainland currently lacks such norms. Furthermore, this study was not suitable for normative research. First, this study only aimed to introduce both short KIDSCREEN versions. Second, to ensure representativeness and generalizability, the construction and presentation of norms have strict requirements for samples (e.g., sampling method, sample size). Generally speaking, in the Chinese Mainland, similar studies require large-scale provincial or even national representative samples [157–159]. Obviously, this study in Weifang City did not meet the conditions for normative research. Future research may benefit from large-scale representative samples to construct and present norms for the Mandarin Chinese version of these instruments, providing references and coordinates for subsequent KIDSCREEN studies in the Chinese Mainland and beyond.

The sixth limitation is that the uniqueness of this study made it difficult to adhere to COSMIN in data analysis and reporting fully; the following four points need to be noted:

- First, this study did not report the content validity of both short KIDSCREEN versions. Content validity is more commonly used and influential in the instrument development stage than in the cross-cultural translation process [160]. Due to the standardized development process of the KIDSCREEN questionnaires [20], their content validity is generally excellent and doesn’t require further evaluation by translators. However, the WFMU-KS-MC research group still conducted expert consultation and content validity evaluation, but only for the original long KIDSCREEN version (KS-52) [40]. We plan to present these results elsewhere in the future. We also encourage other researchers to evaluate the content validity of both short KIDSCREEN versions, which remains an area worthy of study.
- Second, since this study was not based on clinical samples, a series of psychometric properties unique to this sample, e.g., the minimal clinically important difference (MCID), were not evaluated [63].
- Third, this study could not report the predictive validity (a type of criterion validity) [63], because this indicator relies on longitudinal measurement data.
- Finally, this study could not directly verify cross-cultural validity because both methods (MG-CFA and DIF) require independent datasets for multiple cultures. This represents a significant research direction for the future.

## Conclusions

The self-reported KS-27 and KS-10 worked well in the Mandarin Chinese context and had excellent psychometric properties. Moreover, both short KIDSCREEN versions provided concise self-reported HRQoL measures for children and adolescents in the Chinese Mainland. Their ease of administration, scoring, and interpretation made them ideal for routine monitoring in school settings, with great potential for large-scale population surveys and clinical settings. Besides, this study filled the knowledge gap on the validation and usefulness of both short KIDSCREEN versions in national cultures worldwide to measure HRQoL. Further large studies are needed to evaluate both Mandarin Chinese KIDSCREEN versions more fully regarding their clinical and research utility and ability to detect changes in HRQoL following interventions.

## Supplementary Information

Supplementary Material 1.

## Data Availability

The KIDSCREEN questionnaires and translations are protected by copyright, with all rights reserved to the KIDSCREEN Group Europe. Thus, the precise positioning of the WFMU-KS-MC research group is only an international contact partner for the KIDSCREEN study in the Chinese Mainland. They have no right to provide the Mandarin Chinese version without authorization. Therefore, researchers, practitioners, and other potential users interested in using Mandarin Chinese self-reported KIDSCREEN questionnaires (official versions, scoring and interpretation guidelines, etc.) have only one choice. This choice is to contact one of the correspondents of this study— Prof. Dr. Ulrike Ravens-Sieberer (ravens-sieberer@uke.de or QOL@uke.de) or visit the website of KIDSCREEN Group Europe (https://www.kidscreen.org/). With regard to data, due to ethical restrictions, participant-level data cannot be made publicly available. However, we have tried our best to upload the second-hand data we can provide (please see supplementary materials). If the raw dataset is still needed, the datasets performed during this study are available upon reasonable request from another corresponding author—Dr. Yuhang Zhu (zhuyuhang369@163.com).

## Abbreviations

AAPOR: American Association for Public Opinion Research
ANOVA: Analysis of variance
CFA: Confirmatory factor analysis
CFI: Comparative fit index
CHQ: Child Health Questionnaire™
CI: Confidence interval
COSMIN: COnsensus-based Standards for the selection of health status Measurement INstruments
CR: Critical ratio
CTT: Classical test theory
CVI: Content validity index
DIF: Differential item functioning
FAS II: Family Affluence Scale II
GHS: Global HRQoL Score
HR-PRO: Health-related patient reporting outcomes
HRQoL: Health-related quality of life
ICC: Intraclass correlation coefficient
ICHOM: International Consortium for Health Outcomes Measurement
IQR: Interquartile range
IRT: Item response theory
KS-10: KIDSCREEN-10 index
KS-27: KIDSCREEN-27
KS-52: KIDSCREEN-52
M: Mean
Max: Maximum value
MCID: Minimal clinically important difference
Mdn: Median
MG-CFA: Multi-group confirmatory factor analysis
MHS: Mental health status
Min: Minimum value
MIs: Modification indices
MLE: Maximum likelihood estimator
MMT: Modern measurement theory
MV: Missing value
PedsQL™ 4.0: Pediatric Quality of Life Inventory™ Version 4.0 Generic Core Scales
P-P: Probability-probability
PROMs: Patient-reported outcome measures
Q-Q: Quantile-quantile
RMSEA: Root-mean-square error of approximation
SAR: Special Administrative Region
SD: Standard deviation
SDGs: Sustainable Development Goals
SDQ: Strengths and Difficulties Questionnaire
SEM: Standard error of measurement
SES: Socioeconomic status
SRMR: Standardized root-mean-square residual
TDS: Total Difficulties Score
TLI: Tucker-Lewis index
WFMU-KS-MC: Weifang Medical University-KIDSCREEN-Mandarin Chinese
YQOL-R: Youth Quality of Life-Research version
