# Supplementary material for: Measuring health-related quality of life in a Chinese Mainland adolescent population: psychometric properties of the Mandarin Chinese self-reported KIDSCREEN-27 and KIDSCREEN-10 index
Source: BMC Psychol. 2024 Oct 29;12:600. doi: 10.1186/s40359-024-01876-6 (PMC11523896; doi:10.1186/s40359-024-01876-6)
Supplement: Supplementary file 1 — Supplementary Material 1. [file 40359_2024_1876_MOESM1_ESM.docx]

***Online Supplementary Materials for:***

**Measuring health-related quality of life in a Chinese Mainland adolescent population: psychometric properties of the Mandarin Chinese self-reported KIDSCREEN-27 and KIDSCREEN-10 index**

Published in ***BMC Psychology***

**Authors**

Juan Li^1^, Yuhang Zhu^2*^, Gaopei Zhu^3^, Zhenliang Qiu^4^, Jinling Wang^5^, Anne Kaman^6^, Michael Erhart^6^, Adekunle Adedeji^7^, Yongye Liu^8^, Di Wu^9*^, Ulrike Ravens-Sieberer^6*^ and the WFMU-KS-MC research group

^1^Department of Health Management, School of Health Management, Binzhou Medical University, No. 346, Guanhai Road, Laishan, Yantai, Shandong 264003, China

^2^Teaching and Research Section of Health Statistics, School of Public Health, Shandong Second Medical University, No. 7166 Baotong West Street, Weicheng, Weifang, Shandong 261053, China

^3^Department of Health Statistics, School of Public Health, Cheeloo College of Medicine, Shandong University, No. 44 Wenhua West Road, Lixia, Jinan, Shandong 250012, China

^4^No. 1 High School of Anhui Sixian County, No. 172 Sishui Boulevard, Hongcheng Sub-district, Sixian, Suzhou, Anhui 234300, China

^5^Weifang Dongming School, No. 2933 Beigong East Street, Kuiwen, Weifang, Shandong 261061, China ^6^Department of Child and Adolescent Psychiatry, Psychotherapy, and Psychosomatics, Center for Psychosocial Medicine, University Medical Center Hamburg-Eppendorf, Martinistraße 52, W 29, Hamburg 20246, Germany

^7^Faculty of Life Sciences, Hamburg University of Applied Sciences, Ulmenliet 20, Hamburg 21033, Germany

^8^Urban Governance and Design Thrust, Society Hub, The Hong Kong University of Science and Technology (Guangzhou), No. 1 Duxue Road, Nansha, Guangzhou, Guangdong 511458, China

^9^The Second Teaching and Research Section of English Major, School of Foreign Languages, Shandong Second Medical University, No. 7166 Baotong West Street, Weicheng, Weifang, Shandong 261053, China

***Correspondence:**

Yuhang Zhu

[zhuyuhang369@163.com](mailto:zhuyuhang369@163.com)

Di Wu

[wfhag@163.com](mailto:wfhag@163.com)

Ulrike Ravens-Sieberer

[ravens-sieberer@uke.de](mailto:ravens-sieberer@uke.de)

**ORCID iDs**

Juan Li<https://orcid.org/0000-0001-8710-4854>

Yuhang Zhuhttps://orcid.org/0000-0002-9236-2812

Gaopei Zhu<https://orcid.org/0000-0002-2085-9336>

Anne Kaman<http://orcid.org/0000-0001-7498-6229>

Adekunle Adedeji<http://orcid.org/0000-0003-4828-1529>

Yongye Liu<https://orcid.org/0000-0001-5252-8577>

Di Wu<https://orcid.org/0000-0002-4876-7011>

Ulrike Ravens-Sieberer<http://orcid.org/0000-0002-2031-095X>

***Contents***

**Table A** Distribution characteristics of the row data in the baseline and retest surveys of the KIDSCREEN-27 and KIDSCREEN-10 index S5

**Table B.1** Item correlation matrix of the KIDSCREEN-27’s Physical Well-being in the baseline survey

S6

**Table B.2** Item correlation matrix of the KIDSCREEN-27’s Psychological Well-being in the baseline survey S7

**Table B.3** Item correlation matrix of the KIDSCREEN-27’s Autonomy and Parent Relation in the baseline survey S8

**Table B.4** Item correlation matrix of the KIDSCREEN-27’s Social Support and Peers in the baseline survey S9

**Table B.5** Item correlation matrix of the KIDSCREEN-27’s School Environment in the baseline survey S10

**Table B.6** Item correlation matrix of the KIDSCREEN-10 index’s Global HRQoL Score in the baseline survey S11

**Figure A.1** Final revised model of the five-dimensional structure for the KIDSCREEN-27 S12

**Figure A.2** Final revised model of the one-dimensional structure for the KIDSCREEN-10 index S13

**Table C.1** Parameter estimation for the CFA of the KIDSCREEN-27 in the baseline and retest surveys

S14

**Table C.2** Parameter estimation for the CFA of the KIDSCREEN-10 index in the baseline and retest surveys S15

**Table D.1** Specific goodness-of-fit indicators of CFA among different subgroups of the KIDSCREEN-27 in the baseline and retest surveys S16

**Table D.2** Specific goodness-of-fit indicators of CFA among different subgroups of the KIDSCREEN-10 index in the baseline and retest surveys S17

**Table E.1** Item covariance matrix of the KIDSCREEN-27 in the baseline survey S18

**Table E.2** Item covariance matrix of the KIDSCREEN-27 in male sample of the baseline survey S19

**Table E.3** Item covariance matrix of the KIDSCREEN-27 in female sample of the baseline survey S20

**Table E.4** Item covariance matrix of the KIDSCREEN-27 in primary school sample of the baseline survey S21

**Table E.5** Item covariance matrix of the KIDSCREEN-27 in junior secondary school sample of the baseline survey S22

**Table E.6** Item covariance matrix of the KIDSCREEN-27 in senior secondary school sample of the baseline survey S23

**Table F.1** Item covariance matrix of the KIDSCREEN-27 in the retest survey S24

**Table F.2** Item covariance matrix of the KIDSCREEN-27 in male sample of the retest survey S25

**Table F.3** Item covariance matrix of the KIDSCREEN-27 in female sample of the retest survey S26

**Table F.4** Item covariance matrix of the KIDSCREEN-27 in primary school sample of the retest survey S27

**Table F.5** Item covariance matrix of the KIDSCREEN-27 in junior secondary school sample of the retest survey S28

**Table F.6** Item covariance matrix of the KIDSCREEN-27 in senior secondary school sample of the retest survey S29

**Table G.1** Item covariance matrix of the KIDSCREEN-10 index in the baseline survey S30

**Table G.2** Item covariance matrices of the gender subgroup for the KIDSCREEN-10 index in the baseline survey S31

**Table G.3** Item covariance matrices of the grade subgroup for the KIDSCREEN-10 index in the baseline survey S32

**Table H.1** Item covariance matrix of the KIDSCREEN-10 index in the retest survey S33

**Table H.2** Item covariance matrices of the gender subgroup for the KIDSCREEN-10 index in the retest survey S34

**Table H.3** Item covariance matrices of the grade subgroup for the KIDSCREEN-10 index in the retest survey S35

| **Table A** Distribution characteristics of the row data in the baseline and retest surveys of the KIDSCREEN-27 and KIDSCREEN-10 index | | | | | | | | | | | | | | |
| --- | --- | --- | --- | --- | --- | --- | --- | --- | --- | --- | --- | --- | --- | --- |
| **Item and dimension labels** | ***N*^a^** | **Skewness (*SE*)^a^** | ***z*-value of skewness^a^** | **Kurtosis (*SE*)^a^** | ***z*-value of**  **kurtosis^a^** | ***D*-value^a^** | ***W*-value^a^** | ***N*^b^** | **Skewness (*SE*)^b^** | ***z*-value of skewness^b^** | **Kurtosis (*SE*)^b^** | ***z*-value of**  **kurtosis^b^** | ***D*-value^b^** | ***W*-value^b^** |
| **Physical Well-being (KS-27)** | 4301 | −0.304 (0.037) | −8.137 | 1.093 (0.075) | 14.638 | 0.145 | 0.944 | 837 | −1.377 (0.085) | −16.288 | 3.049 (0.169) | 18.057 | 0.213 | 0.883 |
| 1. In general, how would you say health is? | 4363 | −0.906 (0.037) | −24.446 | 2.619 (0.074) | 35.337 | 0.351 | 0.710 | 840 | −0.924 (0.084) | −10.953 | 3.203 (0.169) | 19.004 | 0.351 | 0.706 |
| 2. *Have you felt ﬁt and well?* | 4367 | −0.297 (0.037) | −8.015 | 0.705 (0.074) | 9.522 | 0.350 | 0.765 | 840 | −0.565 (0.084) | −6.695 | 1.303 (0.169) | 7.729 | 0.373 | 0.745 |
| 3. Have you been physically active? | 4367 | −0.178 (0.037) | −4.792 | −0.136 (0.074) | −1.830 | 0.296 | 0.826 | 840 | −0.583 (0.084) | −6.912 | 1.326 (0.169) | 7.869 | 0.374 | 0.750 |
| 4. Have you been able to run well? | 4370 | −0.412 (0.037) | −11.111 | 0.155 (0.074) | 2.091 | 0.296 | 0.789 | 840 | −0.197 (0.084) | −2.332 | 0.693 (0.169) | 4.112 | 0.356 | 0.739 |
| 5. *Have you felt full of energy?* | 4370 | −0.370 (0.037) | −9.993 | 0.587 (0.074) | 7.919 | 0.316 | 0.780 | 840 | −0.481 (0.084) | −5.703 | 1.428 (0.169) | 8.475 | 0.335 | 0.758 |
| **Psychological Well-being (KS-27)** | 4333 | −0.469 (0.037) | −12.603 | −0.379 (0.074) | −5.097 | 0.103 | 0.969 | 829 | −0.746 (0.085) | −8.785 | −0.017 (0.170) | −0.097 | 0.141 | 0.944 |
| 6. Has your life been enjoyable? | 4377 | −0.251 (0.037) | −6.789 | 0.050 (0.074) | 0.679 | 0.282 | 0.844 | 840 | −0.805 (0.084) | −9.539 | 1.896 (0.169) | 11.251 | 0.371 | 0.757 |
| 7. Have you been in a good mood? | 4376 | −0.316 (0.037) | −8.543 | −0.110 (0.074) | −1.493 | 0.270 | 0.847 | 840 | −0.554 (0.084) | −6.566 | 0.214 (0.169) | 1.268 | 0.298 | 0.841 |
| 8. Have you had fun? | 4376 | −0.416 (0.037) | −11.240 | −0.101 (0.074) | −1.370 | 0.264 | 0.842 | 840 | −0.592 (0.084) | −7.016 | 1.023 (0.169) | 6.071 | 0.291 | 0.814 |
| 9. *Have you felt sad?* | 4377 | −0.238 (0.037) | −6.433 | −0.824 (0.074) | −11.137 | 0.204 | 0.861 | 839 | −0.181 (0.084) | −2.145 | −0.608 (0.169) | −3.603 | 0.207 | 0.875 |
| 10. Felt so bad that didn’t want to do anything? | 4379 | −0.422 (0.037) | −11.396 | −0.910 (0.074) | −12.296 | 0.237 | 0.842 | 839 | −0.240 (0.084) | −2.848 | −0.803 (0.169) | −4.762 | 0.201 | 0.872 |
| 11. *Have you felt lonely?* | 4377 | −0.548 (0.037) | −14.812 | −0.504 (0.074) | −6.805 | 0.214 | 0.853 | 838 | −0.317 (0.084) | −3.751 | −0.264 (0.169) | −1.567 | 0.246 | 0.859 |
| 12. Have you been happy with way you are? | 4379 | −0.574 (0.037) | −15.509 | 0.108 (0.074) | 1.464 | 0.309 | 0.846 | 838 | −0.432 (0.084) | −5.110 | −0.153 (0.169) | −0.905 | 0.222 | 0.884 |
| **Autonomy and Parent Relation (KS-27)** | 4304 | −0.195 (0.037) | −5.214 | −0.313 (0.075) | −4.192 | 0.064 | 0.990 | 822 | −0.580 (0.085) | −6.796 | 0.148 (0.170) | 0.870 | 0.106 | 0.969 |
| 13. *Have you had enough time for yourself?* | 4377 | −0.080 (0.037) | −2.170 | −0.880 (0.074) | −11.891 | 0.222 | 0.880 | 838 | −0.243 (0.084) | −2.880 | −0.394 (0.169) | −2.336 | 0.219 | 0.894 |
| 14. *Able to do things—want to do in free time?* | 4379 | −0.299 (0.037) | −8.071 | −0.810 (0.074) | −10.946 | 0.188 | 0.875 | 838 | 0.001 (0.084) | 0.007 | −0.868 (0.169) | −5.144 | 0.214 | 0.892 |
| 15. Parent(s) had enough time for you? | 4378 | −0.190 (0.037) | −5.121 | −0.576 (0.074) | −7.785 | 0.233 | 0.870 | 838 | −0.543 (0.084) | −6.424 | 0.691 (0.169) | 4.094 | 0.318 | 0.825 |
| 16. *Have your parent(s) treated you fairly?* | 4378 | −0.729 (0.037) | −19.687 | 0.753 (0.074) | 10.179 | 0.269 | 0.813 | 839 | −0.646 (0.084) | −7.652 | 0.669 (0.169) | 3.966 | 0.271 | 0.796 |
| 17. Able to talk to parent(s) when wanted to? | 4378 | −0.324 (0.037) | −8.762 | −0.313 (0.074) | −4.231 | 0.244 | 0.869 | 838 | −0.546 (0.084) | −6.466 | 0.135 (0.169) | 0.798 | 0.305 | 0.842 |
| 18. Enough money to do things as friends? | 4363 | 0.341 (0.037) | 9.203 | −0.740 (0.074) | −9.984 | 0.251 | 0.877 | 838 | −0.214 (0.084) | −2.530 | −0.588 (0.169) | −3.486 | 0.196 | 0.906 |
| 19. Had enough money for your expenses? | 4359 | 0.008 (0.037) | 0.208 | −0.875 (0.074) | −11.793 | 0.226 | 0.885 | 839 | −0.343 (0.084) | −4.067 | 0.268 (0.169) | 1.587 | 0.257 | 0.858 |
| **Social Support and Peers (KS-27)** | 4353 | −0.469 (0.037) | −12.627 | 0.357 (0.074) | 4.810 | 0.176 | 0.945 | 830 | −1.311 (0.085) | −15.443 | 1.677 (0.170) | 9.893 | 0.240 | 0.863 |
| 20. Have you spent time with your friends? | 4378 | 0.022 (0.037) | 0.599 | −0.176 (0.074) | −2.378 | 0.253 | 0.857 | 838 | −0.390 (0.084) | −4.613 | 0.357 (0.169) | 2.115 | 0.280 | 0.842 |
| 21. *Have you had fun with your friends?* | 4376 | −0.420 (0.037) | −11.354 | 0.298 (0.074) | 4.027 | 0.295 | 0.806 | 839 | −0.495 (0.084) | −5.860 | 0.336 (0.169) | 1.993 | 0.286 | 0.800 |
| 22. You and your friends helped each other? | 4376 | −0.438 (0.037) | −11.832 | 0.335 (0.074) | 4.531 | 0.293 | 0.796 | 838 | −0.557 (0.084) | −6.595 | 0.831 (0.169) | 4.928 | 0.306 | 0.776 |
| 23. Have you been able to rely on your friends? | 4377 | −0.703 (0.037) | −18.998 | 0.672 (0.074) | 9.081 | 0.287 | 0.800 | 838 | −0.730 (0.084) | −8.637 | 1.514 (0.169) | 8.976 | 0.318 | 0.768 |
| **School Environment (KS-27)** | 4357 | −0.032 (0.037) | −0.874 | 0.383 (0.074) | 5.169 | 0.128 | 0.960 | 829 | −0.506 (0.085) | −5.958 | −0.282 (0.170) | −1.663 | 0.147 | 0.960 |
| 24. Have you been happy at school? | 4380 | −0.367 (0.037) | −9.924 | 0.575 (0.074) | 7.769 | 0.294 | 0.827 | 837 | −0.228 (0.085) | −2.700 | −0.078 (0.169) | −0.463 | 0.267 | 0.858 |
| 25. *Have you got on well at school?* | 4376 | 0.144 (0.037) | 3.901 | 0.289 (0.074) | 3.908 | 0.294 | 0.858 | 838 | 0.237 (0.084) | 2.802 | −0.040 (0.169) | −0.236 | 0.287 | 0.860 |
| 26. *Have you been able to pay attention?* | 4377 | −0.348 (0.037) | −9.412 | 0.058 (0.074) | 0.780 | 0.299 | 0.839 | 838 | −0.766 (0.084) | −9.073 | 0.512 (0.169) | 3.035 | 0.303 | 0.850 |
| 27. Got along well with your teachers? | 4378 | −0.423 (0.037) | −11.432 | 0.268 (0.074) | 3.627 | 0.303 | 0.839 | 838 | −0.650 (0.084) | −7.698 | 0.380 (0.169) | 2.255 | 0.317 | 0.832 |
| **Global HRQoL Score (KS-10)** | 4291 | −0.305 (0.037) | −8.165 | −0.315 (0.075) | −4.215 | 0.067 | 0.987 | 820 | −0.607 (0.085) | −7.108 | −0.104 (0.171) | −0.608 | 0.129 | 0.960 |
| *Note*. *KS-27* KIDSCREEN-27, *KS-10* KIDSCREEN-10 index, *SE* standard error, *D*-value Lilliefors corrected Kolmogorov-Smirnov test statistic, *W*-value Shapiro-Wilk test statistic  *z*-value of skewness = skewness value/*SE* of skewness, *z*-value of kurtosis = kurtosis value/*SE* of kurtosis  KS-27 included all 27 items; KS-10 included items 2, 5, 9, 11, 13, 14, 16, 21, 25, and 26 (indicated in *italics*). The listwise (casewise) deletion method is used to deal with missing values  ^a^ the baseline survey, ^b^ the retest survey  Two kinds of data distribution tests (Lilliefors corrected Kolmogorov-Smirnov and Shapiro-Wilk) are statistically significant at *p* < 0.001 level | | | | | | | | | | | | | | |

| **Table B.1** Item correlation matrix of the KIDSCREEN-27’s Physical Well-being in the baseline survey | | | | | |
| --- | --- | --- | --- | --- | --- |
|  | **Item1** | **Item2** | **Item3** | **Item4** | **Item5** |
| Item1 | **1.000**  (4363) |  |  |  |  |
| Item2 | 0.592^**^  (4346) | **1.000**  (4367) |  |  |  |
| Item3 | 0.498^**^  (4345) | 0.560^**^  (4349) | **1.000**  (4367) |  |  |
| Item4 | 0.535^**^  (4348) | 0.542^**^  (4353) | 0.599^**^  (4353) | **1.000**  (4370) |  |
| Item5 | 0.554^**^  (4348) | 0.542^**^  (4352) | 0.517^**^  (4352) | 0.501^**^  (4356) | **1.000**  (4370) |
| *Note*. The listwise (casewise) deletion method is used to deal with missing values. All Pearson correlation coefficients. Figures in brackets indicate paired cases  ^**^ *p* < 0.01 | | | | | |

| **Table B.2** Item correlation matrix of the KIDSCREEN-27’s Psychological Well-being in the baseline survey | | | | | | | |
| --- | --- | --- | --- | --- | --- | --- | --- |
|  | **Item6** | **Item7** | **Item8** | **Item9** | **Item10** | **Item11** | **Item12** |
| Item6 | **1.000**  (4377) |  |  |  |  |  |  |
| Item7 | 0.692^**^  (4369) | **1.000**  (4376) |  |  |  |  |  |
| Item8 | 0.670^**^  (4368) | 0.695^**^  (4367) | **1.000**  (4376) |  |  |  |  |
| Item9 | 0.467^**^  (4369) | 0.450^**^  (4368) | 0.426^**^  (4368) | **1.000**  (4377) |  |  |  |
| Item10 | 0.428^**^  (4371) | 0.430^**^  (4371) | 0.402^**^  (4370) | 0.799^**^  (4371) | **1.000**  (4379) |  |  |
| Item11 | 0.472^**^  (4369) | 0.448^**^  (4368) | 0.446^**^  (4368) | 0.738^**^  (4369) | 0.739^**^  (4371) | **1.000**  (4377) |  |
| Item12 | 0.406^**^  (4371) | 0.384^**^  (4370) | 0.362^**^  (4370) | 0.466^**^  (4371) | 0.470^**^  (4373) | 0.457^**^  (4371) | **1.000**  (4379) |
| *Note*. The listwise (casewise) deletion method is used to deal with missing values. All Pearson correlation coefficients. Figures in brackets indicate paired cases  ^**^ *p* < 0.01 | | | | | | | |

| **Table B.3** Item correlation matrix of the KIDSCREEN-27’s Autonomy and Parent Relation in the baseline survey | | | | | | | |
| --- | --- | --- | --- | --- | --- | --- | --- |
|  | **Item13** | **Item14** | **Item15** | **Item16** | **Item17** | **Item18** | **Item19** |
| Item13 | **1.000**  (4377) |  |  |  |  |  |  |
| Item14 | 0.873^**^  (4371) | **1.000**  (4379) |  |  |  |  |  |
| Item15 | 0.286^**^  (4370) | 0.293^**^  (4372) | **1.000**  (4378) |  |  |  |  |
| Item16 | 0.305^**^  (4370) | 0.310^**^  (4372) | 0.609^**^  (4371) | **1.000**  (4378) |  |  |  |
| Item17 | 0.317^**^  (4370) | 0.329^**^  (4372) | 0.669^**^  (4371) | 0.643^**^  (4371) | **1.000**  (4378) |  |  |
| Item18 | 0.177^**^  (4355) | 0.183^**^  (4357) | 0.191^**^  (4356) | 0.202^**^  (4356) | 0.237^**^  (4356) | **1.000**  (4363) |  |
| Item19 | 0.210^**^  (4351) | 0.217^**^  (4353) | 0.173^**^  (4352) | 0.249^**^  (4352) | 0.228^**^  (4352) | 0.771^**^  (4339) | **1.000**  (4359) |
| *Note*. The listwise (casewise) deletion method is used to deal with missing values. All Pearson correlation coefficients. Figures in brackets indicate paired cases  ^**^ *p* < 0.01 | | | | | | | |

| **Table B.4** Item correlation matrix of the KIDSCREEN-27’s Social Support and Peers in the baseline survey | | | | |
| --- | --- | --- | --- | --- |
|  | **Item20** | **Item21** | **Item22** | **Item23** |
| Item20 | **1.000**  (4378) |  |  |  |
| Item21 | 0.627^**^  (4369) | **1.000**  (4376) |  |  |
| Item22 | 0.608^**^  (4369) | 0.770^**^  (4367) | **1.000**  (4376) |  |
| Item23 | 0.576^**^  (4370) | 0.646^**^  (4368) | 0.663^**^  (4369) | **1.000**  (4377) |
| *Note*. The listwise (casewise) deletion method is used to deal with missing values. All Pearson correlation coefficients. Figures in brackets indicate paired cases  ^**^ *p* < 0.01 | | | | |

| **Table B.5** Item correlation matrix of the KIDSCREEN-27’s School Environment in the baseline survey | | | | |
| --- | --- | --- | --- | --- |
|  | **Item24** | **Item25** | **Item26** | **Item27** |
| Item24 | **1.000**  (4380) |  |  |  |
| Item25 | 0.592^**^  (4371) | **1.000**  (4376) |  |  |
| Item26 | 0.628^**^  (4372) | 0.687^**^  (4369) | **1.000**  (4377) |  |
| Item27 | 0.637^**^  (4373) | 0.631^**^  (4369) | 0.686^**^  (4370) | **1.000**  (4378) |
| *Note*. The listwise (casewise) deletion method is used to deal with missing values. All Pearson correlation coefficients. Figures in brackets indicate paired cases  ^**^ *p* < 0.01 | | | | |

| **Table B.6** Item correlation matrix of the KIDSCREEN-10 index’s Global HRQoL Score in the baseline survey | | | | | | | | | | |
| --- | --- | --- | --- | --- | --- | --- | --- | --- | --- | --- |
|  | **Item2** | **Item5** | **Item9** | **Item11** | **Item13** | **Item14** | **Item16** | **Item21** | **Item25** | **Item26** |
| Item2 | **1.000**  (4367) |  |  |  |  |  |  |  |  |  |
| Item5 | 0.542^**^  (4352) | **1.000**  (4370) |  |  |  |  |  |  |  |  |
| Item9 | 0.298^**^  (4359) | 0.290^**^  (4362) | **1.000**  (4377) |  |  |  |  |  |  |  |
| Item11 | 0.306^**^  (4359) | 0.304^**^  (4362) | 0.738^**^  (4369) | **1.000**  (4377) |  |  |  |  |  |  |
| Item13 | 0.244^**^  (4359) | 0.217^**^  (4362) | 0.346^**^  (4369) | 0.348^**^  (4369) | **1.000**  (4377) |  |  |  |  |  |
| Item14 | 0.226^**^  (4361) | 0.212^**^  (4364) | 0.333^**^  (4371) | 0.343^**^  (4371) | 0.873^**^  (4371) | **1.000**  (4379) |  |  |  |  |
| Item16 | 0.185^**^  (4360) | 0.209^**^  (4363) | 0.322^**^  (4370) | 0.364^**^  (4370) | 0.305^**^  (4370) | 0.310^**^  (4372) | **1.000**  (4378) |  |  |  |
| Item21 | 0.260^**^  (4358) | 0.285^**^  (4361) | 0.372^**^  (4368) | 0.396^**^  (4368) | 0.296^**^  (4368) | 0.287^**^  (4370) | 0.358^**^  (4369) | **1.000**  (4376) |  |  |
| Item25 | 0.317^**^  (4358) | 0.303^**^  (4362) | 0.426^**^  (4368) | 0.409^**^  (4368) | 0.359^**^  (4368) | 0.354^**^  (4370) | 0.309^**^  (4369) | 0.404^**^  (4367) | **1.000**  (4376) |  |
| Item26 | 0.297^**^  (4359) | 0.299^**^  (4362) | 0.442^**^  (4369) | 0.449^**^  (4369) | 0.331^**^  (4369) | 0.326^**^  (4371) | 0.364^**^  (4370) | 0.435^**^  (4368) | 0.687^**^  (4369) | **1.000**  (4377) |
| *Note*. The listwise (casewise) deletion method is used to deal with missing values. All Pearson correlation coefficients. Figures in brackets indicate paired cases  ^**^ *p* < 0.01 | | | | | | | | | | |

**Figure A.1** Final revised model of the five-dimensional structure for the KIDSCREEN-27

**Figure A.2** Final revised model of the one-dimensional structure for the KIDSCREEN-10 index

| **Table C.1** Parameter estimation for the CFA of the KIDSCREEN-27 in the baseline and retest surveys | | | | | | | | | | | | | | |
| --- | --- | --- | --- | --- | --- | --- | --- | --- | --- | --- | --- | --- | --- | --- |
| **DIM** | **Item** | **Baseline survey (*N* = 4114)** | | | | | |  | **Retest survey (*N* = 789)** | | | | | |
|  |  | **Unstandardized** | | |  | **Standardized** | |  | **Unstandardized** | | |  | **Standardized** | |
|  |  | ***Est.*** | ***SE*** | ***CR*** |  | ***Est.*** | **δ** |  | ***Est.*** | ***SE*** | ***CR*** |  | ***Est.*** | **δ** |
| **Factor loading** | | |  |  |  |  |  |  |  |  |  |  |  |  |
| PHB | 1 | 1.000 | — | — |  | 0.734 | 0.181 |  | 1.000 | — | — |  | 0.627 | 0.237 |
|  | 2 | 0.995 | 0.022 | 45.235^***^ |  | 0.762 | 0.151 |  | 0.870 | 0.076 | 11.413^***^ |  | 0.567 | 0.245 |
|  | 3 | 1.097 | 0.025 | 43.254^***^ |  | 0.727 | 0.227 |  | 0.862 | 0.076 | 11.292^***^ |  | 0.558 | 0.252 |
|  | 4 | 1.043 | 0.024 | 43.565^***^ |  | 0.732 | 0.199 |  | 0.779 | 0.071 | 10.924^***^ |  | 0.531 | 0.237 |
|  | 5 | 0.977 | 0.023 | 42.380^***^ |  | 0.711 | 0.197 |  | 0.764 | 0.073 | 10.412^***^ |  | 0.497 | 0.273 |
| PWB | 6 | 1.000 | — | — |  | 0.550 | 0.391 |  | 1.000 | — | — |  | 0.461 | 0.32 |
|  | 7 | 0.994 | 0.024 | 42.072^***^ |  | 0.533 | 0.422 |  | 1.210 | 0.121 | 10.027^***^ |  | 0.447 | 0.509 |
|  | 8 | 0.965 | 0.024 | 39.762^***^ |  | 0.513 | 0.442 |  | 1.037 | 0.111 | 9.304^***^ |  | 0.426 | 0.421 |
|  | 9 | 1.976 | 0.052 | 37.631^***^ |  | 0.877 | 0.199 |  | 2.006 | 0.188 | 10.677^***^ |  | 0.647 | 0.483 |
|  | 10 | 2.155 | 0.057 | 37.612^***^ |  | 0.876 | 0.239 |  | 2.058 | 0.193 | 10.683^***^ |  | 0.648 | 0.506 |
|  | 11 | 1.976 | 0.054 | 36.838^***^ |  | 0.838 | 0.281 |  | 1.661 | 0.161 | 10.303^***^ |  | 0.596 | 0.433 |
|  | 12 | 1.138 | 0.039 | 29.012^***^ |  | 0.565 | 0.468 |  | 1.397 | 0.162 | 8.633^***^ |  | 0.433 | 0.733 |
| APR | 13 | 1.000 | — | — |  | 0.429 | 0.832 |  | 1.000 | — | — |  | 0.404 | 0.729 |
|  | 14 | 1.020 | 0.020 | 50.364^***^ |  | 0.435 | 0.838 |  | 1.105 | 0.112 | 9.907^***^ |  | 0.405 | 0.884 |
|  | 15 | 1.500 | 0.058 | 25.876^***^ |  | 0.769 | 0.292 |  | 0.969 | 0.122 | 7.945^***^ |  | 0.495 | 0.412 |
|  | 16 | 1.340 | 0.052 | 25.931^***^ |  | 0.774 | 0.225 |  | 0.925 | 0.116 | 7.976^***^ |  | 0.499 | 0.366 |
|  | 17 | 1.614 | 0.061 | 26.322^***^ |  | 0.821 | 0.236 |  | 1.035 | 0.131 | 7.918^***^ |  | 0.491 | 0.479 |
|  | 18 | 0.773 | 0.048 | 15.975^***^ |  | 0.312 | 1.041 |  | 1.166 | 0.157 | 7.429^***^ |  | 0.433 | 0.838 |
|  | 19 | 0.754 | 0.046 | 16.489^***^ |  | 0.326 | 0.899 |  | 0.836 | 0.118 | 7.083^***^ |  | 0.398 | 0.528 |
| SSP | 20 | 1.000 | — | — |  | 0.718 | 0.290 |  | 1.000 | — | — |  | 0.585 | 0.367 |
|  | 21 | 1.064 | 0.020 | 52.421^***^ |  | 0.865 | 0.118 |  | 0.955 | 0.079 | 12.145^***^ |  | 0.614 | 0.288 |
|  | 22 | 1.047 | 0.020 | 52.666^***^ |  | 0.870 | 0.109 |  | 0.998 | 0.078 | 12.774^***^ |  | 0.674 | 0.228 |
|  | 23 | 0.994 | 0.021 | 46.771^***^ |  | 0.766 | 0.215 |  | 0.986 | 0.079 | 12.489^***^ |  | 0.644 | 0.261 |
| SCE | 24 | 1.000 | — | — |  | 0.770 | 0.209 |  | 1.000 | — | — |  | 0.725 | 0.286 |
|  | 25 | 1.200 | 0.023 | 52.160^***^ |  | 0.792 | 0.259 |  | 0.961 | 0.060 | 15.930^***^ |  | 0.676 | 0.347 |
|  | 26 | 1.138 | 0.020 | 55.805^***^ |  | 0.843 | 0.160 |  | 0.964 | 0.067 | 14.409^***^ |  | 0.600 | 0.524 |
|  | 27 | 1.108 | 0.021 | 53.613^***^ |  | 0.812 | 0.192 |  | 0.764 | 0.059 | 12.941^***^ |  | 0.532 | 0.468 |
| **Covariance (Correlation)** | | | | | | | | | | | | | | |
| PHB$\leftrightarrow$PWB | | 0.088 | 0.004 | 20.063^***^ |  | 0.467 | — |  | 0.058 | 0.008 | 7.231^***^ |  | 0.502 | — |
| PHB$\leftrightarrow$APR | | 0.074 | 0.005 | 15.564^***^ |  | 0.371 | — |  | 0.069 | 0.011 | 6.185^***^ |  | 0.465 | — |
| PHB$\leftrightarrow$SSP | | 0.111 | 0.005 | 20.600^***^ |  | 0.436 | — |  | 0.066 | 0.010 | 6.548^***^ |  | 0.387 | — |
| PHB$\leftrightarrow$SCE | | 0.116 | 0.005 | 21.640^***^ |  | 0.460 | — |  | 0.099 | 0.013 | 7.698^***^ |  | 0.448 | — |
| PWB$\leftrightarrow$APR | | 0.099 | 0.005 | 17.965^***^ |  | 0.553 | — |  | 0.073 | 0.011 | 6.744^***^ |  | 0.661 | — |
| PWB$\leftrightarrow$SSP | | 0.123 | 0.006 | 21.827^***^ |  | 0.537 | — |  | 0.070 | 0.009 | 7.506^***^ |  | 0.549 | — |
| PWB$\leftrightarrow$SCE | | 0.147 | 0.006 | 24.123^***^ |  | 0.647 | — |  | 0.118 | 0.013 | 8.935^***^ |  | 0.714 | — |
| APR$\leftrightarrow$SSP | | 0.128 | 0.007 | 18.787^***^ |  | 0.533 | — |  | 0.083 | 0.013 | 6.443^***^ |  | 0.504 | — |
| APR$\leftrightarrow$SCE | | 0.134 | 0.007 | 19.356^***^ |  | 0.560 | — |  | 0.149 | 0.019 | 7.770^***^ |  | 0.703 | — |
| SSP$\leftrightarrow$SCE | | 0.195 | 0.007 | 26.810^***^ |  | 0.637 | — |  | 0.140 | 0.016 | 8.934^***^ |  | 0.568 | — |
| err_13_$\leftrightarrow$err_14_ | | 0.703 | 0.018 | 39.593^***^ |  | 0.842 | — |  | 0.370 | 0.035 | 10.573^***^ |  | 0.461 | — |
| err_18_$\leftrightarrow$err_19_ | | 0.722 | 0.019 | 37.558^***^ |  | 0.747 | — |  | 0.309 | 0.029 | 10.561^***^ |  | 0.464 | — |
| err_7_$\leftrightarrow$err_8_ | | 0.248 | 0.008 | 30.644^***^ |  | 0.575 | — |  | 0.081 | 0.018 | 4.466^***^ |  | 0.175 | — |
| err_6_$\leftrightarrow$err_7_ | | 0.228 | 0.008 | 29.961^***^ |  | 0.562 | — |  | 0.089 | 0.016 | 5.543^***^ |  | 0.222 | — |
| err_6_$\leftrightarrow$err_8_ | | 0.224 | 0.008 | 29.064^***^ |  | 0.538 | — |  | 0.056 | 0.014 | 3.895^***^ |  | 0.153 | — |
| *Note*. *CFA* confirmatory factor analysis, *DIM* dimension, *PHB* Physical Well-being, *PWB* Psychological Well-being, *APR* Autonomy and Parent Relation, *SSP* Social Support and Peers, *SCE* School Environment  *Est.* estimate, *SE* standard error, *CR* critical ratio, equivalent to the common *t*-value, *δ* residual, *err* error  ^***^ *p* < 0.001 | | | | | | | | | | | | | | |

| **Table C.2** Parameter estimation for the CFA of the KIDSCREEN-10 index in the baseline and retest surveys | | | | | | | | | | | | | | |
| --- | --- | --- | --- | --- | --- | --- | --- | --- | --- | --- | --- | --- | --- | --- |
| **DIM** | **Item** | **Baseline survey (*N* = 4291)** | | | | | |  | **Retest survey (*N* = 820)** | | | | | |
|  |  | **Unstandardized** | | |  | **Standardized** | |  | **Unstandardized** | | |  | **Standardized** | |
|  |  | ***Est.*** | ***SE*** | ***CR*** |  | ***Est.*** | **δ** |  | ***Est.*** | ***SE*** | ***CR*** |  | ***Est.*** | **δ** |
| **Factor loading** | | |  |  |  |  |  |  |  |  |  |  |  |  |
| GHS | 2 | 1.000 | — | — |  | 0.417 | 0.300 |  | 1.000 | — | — |  | 0.314 | 0.332 |
|  | 5 | 1.050 | 0.040 | 26.019^***^ |  | 0.416 | 0.334 |  | 1.068 | 0.167 | 6.388^***^ |  | 0.334 | 0.330 |
|  | 9 | 2.162 | 0.093 | 23.241^***^ |  | 0.586 | 0.566 |  | 2.336 | 0.351 | 6.648^***^ |  | 0.490 | 0.629 |
|  | 11 | 2.313 | 0.099 | 23.406^***^ |  | 0.596 | 0.614 |  | 2.185 | 0.325 | 6.728^***^ |  | 0.509 | 0.495 |
|  | 13 | 1.950 | 0.092 | 21.196^***^ |  | 0.484 | 0.786 |  | 1.844 | 0.303 | 6.086^***^ |  | 0.377 | 0.746 |
|  | 14 | 1.918 | 0.092 | 20.908^***^ |  | 0.474 | 0.805 |  | 1.944 | 0.323 | 6.010^***^ |  | 0.362 | 0.911 |
|  | 16 | 1.443 | 0.069 | 20.939^***^ |  | 0.481 | 0.438 |  | 1.292 | 0.221 | 5.859^***^ |  | 0.354 | 0.425 |
|  | 21 | 1.554 | 0.068 | 22.862^***^ |  | 0.570 | 0.317 |  | 1.422 | 0.229 | 6.215^***^ |  | 0.398 | 0.390 |
|  | 25 | 2.585 | 0.103 | 25.187^***^ |  | 0.780 | 0.273 |  | 2.553 | 0.362 | 7.052^***^ |  | 0.607 | 0.407 |
|  | 26 | 2.379 | 0.094 | 25.203^***^ |  | 0.802 | 0.199 |  | 2.903 | 0.413 | 7.034^***^ |  | 0.606 | 0.529 |
| **Covariance (Correlation)** | | | | | | | | | | | | | | |
| err_13_$\leftrightarrow$err_14_ | | 0.664 | 0.017 | 39.208^***^ |  | 0.835 | — |  | 0.392 | 0.035 | 11.313^***^ |  | 0.476 | — |
| err_9_$\leftrightarrow$err_11_ | | 0.351 | 0.012 | 28.922^***^ |  | 0.596 | — |  | 0.105 | 0.024 | 4.325^***^ |  | 0.188 | — |
| err_2_$\leftrightarrow$err_5_ | | 0.141 | 0.006 | 25.233^***^ |  | 0.444 | — |  | 0.062 | 0.012 | 4.961^***^ |  | 0.187 | — |
| *Note*. *CFA* confirmatory factor analysis, *DIM* dimension, *GHS* Global HRQoL Score  *Est.* estimate, *SE* standard error, *CR* critical ratio, equivalent to the common *t*-value, *δ* residual, *err* error  ^***^ *p* < 0.001 | | | | | | | | | | | | | | |

| **Table D.1** Specific goodness-of-fit indicators of CFA among different subgroups of the KIDSCREEN-27 in the baseline and retest surveys | | | | | | | |
| --- | --- | --- | --- | --- | --- | --- | --- |
| **Model** | **Model goodness-of-fit indicators** | | | | | | |
|  | ***N*** | **χ^2^ (*df*)** | **χ^2^/*df*** | **CFI** | **TLI** | **SRMR** | **RMSEA [90% CI]** |
| **Baseline survey** |  |  |  |  |  |  |  |
| Full sample | 4114 | 3948.857^***^ (309) | 12.779 | 0.946 | 0.938 | 0.089 | 0.054 [0.052, 0.055] |
| Gender groups |  |  |  |  |  |  |  |
| Male sample | 1921 | 2024.389^***^ (309) | 6.551 | 0.947 | 0.939 | 0.089 | 0.054 [0.052, 0.056] |
| Female sample | 2193 | 2279.065^***^ (309) | 7.376 | 0.943 | 0.936 | 0.091 | 0.054 [0.052, 0.056] |
| Grade groups |  |  |  |  |  |  |  |
| Primary school sample | 912 | 1131.445^***^ (309) | 3.662 | 0.936 | 0.927 | 0.066 | 0.054 [0.051, 0.057] |
| Junior secondary school sample | 2022 | 1920.401^***^ (309) | 6.215 | 0.953 | 0.947 | 0.076 | 0.051 [0.049, 0.053] |
| Senior secondary school sample | 1180 | 1521.996^***^ (309) | 4.926 | 0.931 | 0.921 | 0.107 | 0.058 [0.055, 0.061] |
| **Retest survey** |  |  |  |  |  |  |  |
| Full sample | 789 | 483.231^***^ (309) | 1.564 | 0.962 | 0.956 | 0.038 | 0.027 [0.022, 0.031] |
| Gender groups |  |  |  |  |  |  |  |
| Male sample | 381 | 406.585^***^ (309) | 1.316 | 0.958 | 0.953 | 0.046 | 0.029 [0.021, 0.036] |
| Female sample | 408 | 392.066^***^ (309) | 1.269 | 0.962 | 0.957 | 0.046 | 0.026 [0.017, 0.033] |
| Grade groups |  |  |  |  |  |  |  |
| Primary school sample | 162 | 329.332^a^ (309) | 1.066 | 0.959 | 0.954 | 0.061 | 0.020 [0.000, 0.037] |
| Junior secondary school sample | 355 | 398.201^***^ (309) | 1.289 | 0.961 | 0.956 | 0.053 | 0.029 [0.020, 0.036] |
| Senior secondary school sample | 272 | 396.870^***^ (309) | 1.284 | 0.936 | 0.927 | 0.057 | 0.032 [0.022, 0.041] |
| Cutoff value | N/A | N/A | < 3 | > 0.90 | > 0.90 | ≤ 0.08 | ≤ 0.08 |
| *Note*. *CFA* confirmatory factor analysis, *CFI* comparative fit index, *TLI* Tucker-Lewis index, *SRMR* standardized root-mean-square residual, *RMSEA* root-mean-square error of approximation, *CI* confidence interval, *N/A* not applicable. The Listwise (casewise) deletion method is used to deal with missing values  ^***^ *p* < 0.001. ^a^ *p* = 0.204 | | | | | | | |

| **Table D.2** Specific goodness-of-fit indicators of CFA among different subgroups of the KIDSCREEN-10 index in the baseline and retest surveys | | | | | | | |
| --- | --- | --- | --- | --- | --- | --- | --- |
| **Model** | **Model goodness-of-fit indicators** | | | | | | |
|  | ***N*** | **χ^2^ (*df*)** | **χ^2^/*df*** | **CFI** | **TLI** | **SRMR** | **RMSEA [90% CI]** |
| **Baseline survey** |  |  |  |  |  |  |  |
| Full sample | 4291 | 610.347^***^ (32) | 19.073 | 0.970 | 0.958 | 0.040 | 0.065 [0.061, 0.070] |
| Gender groups |  |  |  |  |  |  |  |
| Male sample | 2011 | 310.359^***^ (32) | 9.699 | 0.971 | 0.959 | 0.042 | 0.066 [0.059, 0.073] |
| Female sample | 2280 | 318.450^***^ (32) | 9.952 | 0.971 | 0.959 | 0.038 | 0.063 [0.057, 0.069] |
| Grade groups |  |  |  |  |  |  |  |
| Primary school sample | 947 | 188.744^***^ (32) | 5.898 | 0.960 | 0.944 | 0.046 | 0.072 [0.062, 0.082] |
| Junior secondary school sample | 2110 | 375.083^***^ (32) | 11.721 | 0.964 | 0.950 | 0.047 | 0.071 [0.065, 0.078] |
| Senior secondary school sample | 1234 | 191.084^***^ (32) | 5.971 | 0.965 | 0.951 | 0.040 | 0.064 [0.055, 0.072] |
| **Retest survey** |  |  |  |  |  |  |  |
| Full sample | 820 | 36.212^a^ (32) | 1.132 | 0.996 | 0.995 | 0.021 | 0.013 [0.000, 0.030] |
| Gender groups |  |  |  |  |  |  |  |
| Male sample | 396 | 49.794^b^ (32) | 1.556 | 0.969 | 0.956 | 0.036 | 0.038 [0.014, 0.057] |
| Female sample | 424 | 28.865^c^ (32) | 0.902 | 1.000 | 1.008 | 0.028 | 0.000 [0.000, 0.031] |
| Grade groups |  |  |  |  |  |  |  |
| Primary school sample | 169 | 36.336^d^ (32) | 1.136 | 0.945 | 0.923 | 0.054 | 0.028 [0.000, 0.066] |
| Junior secondary school sample | 368 | 32.071^e^ (32) | 1.002 | 1.000 | 1.000 | 0.032 | 0.003 [0.000, 0.039] |
| Senior secondary school sample | 283 | 33.194^f^ (32) | 1.037 | 0.996 | 0.995 | 0.036 | 0.012 [0.000, 0.046] |
| Cutoff value | N/A | N/A | < 3 | > 0.90 | > 0.90 | ≤ 0.08 | ≤ 0.08 |
| *Note*. *CFA* confirmatory factor analysis, *CFI* comparative fit index, *TLI* Tucker-Lewis index, *SRMR* standardized root-mean-square residual, *RMSEA* root-mean-square error of approximation, *CI* confidence interval, *N/A* not applicable. The Listwise (casewise) deletion method is used to deal with missing values  ^***^ *p* < 0.001. ^a^ *p* = 0.278. ^b^ *p* = 0.023. ^c^ *p* = 0.626. ^d^ *p* = 0.274. ^e^ *p* = 0.463. ^f^ *p* = 0.409 | | | | | | | |

| **Table E.1** Item covariance matrix of the KIDSCREEN-27 in the baseline survey (*N* = 4114) | | | | | | | | | | | | | | | | | | | | | | | | | | | |
| --- | --- | --- | --- | --- | --- | --- | --- | --- | --- | --- | --- | --- | --- | --- | --- | --- | --- | --- | --- | --- | --- | --- | --- | --- | --- | --- | --- |
|  | **Item1** | **Item2** | **Item3** | **Item4** | **Item5** | **Item6** | **Item7** | **Item8** | **Item9** | **Item10** | **Item11** | **Item12** | **Item13** | **Item14** | **Item15** | **Item16** | **Item17** | **Item18** | **Item19** | **Item20** | **Item21** | **Item22** | **Item23** | **Item24** | **Item25** | **Item26** | **Item27** |
| Item1 | **0.392** |  |  |  |  |  |  |  |  |  |  |  |  |  |  |  |  |  |  |  |  |  |  |  |  |  |  |
| Item2 | 0.219 | **0.359** |  |  |  |  |  |  |  |  |  |  |  |  |  |  |  |  |  |  |  |  |  |  |  |  |  |
| Item3 | 0.213 | 0.231 | **0.481** |  |  |  |  |  |  |  |  |  |  |  |  |  |  |  |  |  |  |  |  |  |  |  |  |
| Item4 | 0.216 | 0.210 | 0.270 | **0.428** |  |  |  |  |  |  |  |  |  |  |  |  |  |  |  |  |  |  |  |  |  |  |  |
| Item5 | 0.216 | 0.203 | 0.223 | 0.204 | **0.398** |  |  |  |  |  |  |  |  |  |  |  |  |  |  |  |  |  |  |  |  |  |  |
| Item6 | 0.141 | 0.155 | 0.149 | 0.147 | 0.158 | **0.560** |  |  |  |  |  |  |  |  |  |  |  |  |  |  |  |  |  |  |  |  |  |
| Item7 | 0.134 | 0.138 | 0.135 | 0.134 | 0.161 | 0.396 | **0.589** |  |  |  |  |  |  |  |  |  |  |  |  |  |  |  |  |  |  |  |  |
| Item8 | 0.132 | 0.141 | 0.144 | 0.143 | 0.153 | 0.387 | 0.411 | **0.600** |  |  |  |  |  |  |  |  |  |  |  |  |  |  |  |  |  |  |  |
| Item9 | 0.168 | 0.165 | 0.154 | 0.155 | 0.164 | 0.318 | 0.312 | 0.300 | **0.860** |  |  |  |  |  |  |  |  |  |  |  |  |  |  |  |  |  |  |
| Item10 | 0.181 | 0.183 | 0.178 | 0.173 | 0.191 | 0.317 | 0.327 | 0.308 | 0.750 | **1.026** |  |  |  |  |  |  |  |  |  |  |  |  |  |  |  |  |  |
| Item11 | 0.180 | 0.175 | 0.177 | 0.174 | 0.179 | 0.337 | 0.327 | 0.330 | 0.665 | 0.724 | **0.943** |  |  |  |  |  |  |  |  |  |  |  |  |  |  |  |  |
| Item12 | 0.148 | 0.140 | 0.130 | 0.133 | 0.152 | 0.248 | 0.241 | 0.227 | 0.357 | 0.392 | 0.365 | **0.688** |  |  |  |  |  |  |  |  |  |  |  |  |  |  |  |
| Item13 | 0.127 | 0.148 | 0.151 | 0.154 | 0.137 | 0.283 | 0.250 | 0.261 | 0.324 | 0.358 | 0.343 | 0.296 | **1.020** |  |  |  |  |  |  |  |  |  |  |  |  |  |  |
| Item14 | 0.115 | 0.138 | 0.142 | 0.141 | 0.134 | 0.271 | 0.243 | 0.253 | 0.312 | 0.351 | 0.339 | 0.294 | 0.895 | **1.034** |  |  |  |  |  |  |  |  |  |  |  |  |  |
| Item15 | 0.110 | 0.090 | 0.105 | 0.102 | 0.123 | 0.244 | 0.239 | 0.237 | 0.234 | 0.259 | 0.266 | 0.206 | 0.245 | 0.251 | **0.714** |  |  |  |  |  |  |  |  |  |  |  |  |
| Item16 | 0.093 | 0.082 | 0.085 | 0.100 | 0.096 | 0.222 | 0.216 | 0.223 | 0.221 | 0.256 | 0.261 | 0.213 | 0.230 | 0.235 | 0.385 | **0.562** |  |  |  |  |  |  |  |  |  |  |  |
| Item17 | 0.120 | 0.100 | 0.110 | 0.106 | 0.137 | 0.252 | 0.247 | 0.249 | 0.266 | 0.299 | 0.299 | 0.235 | 0.271 | 0.282 | 0.479 | 0.407 | **0.725** |  |  |  |  |  |  |  |  |  |  |
| Item18 | 0.114 | 0.148 | 0.172 | 0.153 | 0.154 | 0.192 | 0.201 | 0.187 | 0.277 | 0.315 | 0.259 | 0.208 | 0.186 | 0.192 | 0.169 | 0.159 | 0.212 | **1.154** |  |  |  |  |  |  |  |  |  |
| Item19 | 0.101 | 0.139 | 0.140 | 0.147 | 0.128 | 0.177 | 0.191 | 0.184 | 0.263 | 0.317 | 0.261 | 0.219 | 0.207 | 0.215 | 0.143 | 0.185 | 0.191 | 0.832 | **1.006** |  |  |  |  |  |  |  |  |
| Item20 | 0.122 | 0.120 | 0.134 | 0.130 | 0.141 | 0.185 | 0.200 | 0.208 | 0.224 | 0.247 | 0.239 | 0.206 | 0.246 | 0.245 | 0.169 | 0.166 | 0.201 | 0.264 | 0.244 | **0.599** |  |  |  |  |  |  |  |
| Item21 | 0.108 | 0.106 | 0.112 | 0.121 | 0.122 | 0.189 | 0.203 | 0.219 | 0.233 | 0.247 | 0.257 | 0.192 | 0.202 | 0.195 | 0.177 | 0.182 | 0.197 | 0.210 | 0.198 | 0.331 | **0.468** |  |  |  |  |  |  |
| Item22 | 0.111 | 0.107 | 0.115 | 0.118 | 0.124 | 0.182 | 0.202 | 0.204 | 0.226 | 0.243 | 0.252 | 0.188 | 0.196 | 0.190 | 0.175 | 0.185 | 0.203 | 0.218 | 0.209 | 0.314 | 0.353 | **0.448** |  |  |  |  |  |
| Item23 | 0.123 | 0.123 | 0.119 | 0.134 | 0.130 | 0.191 | 0.200 | 0.213 | 0.255 | 0.277 | 0.288 | 0.222 | 0.213 | 0.218 | 0.161 | 0.196 | 0.206 | 0.244 | 0.244 | 0.318 | 0.316 | 0.318 | **0.521** |  |  |  |  |
| Item24 | 0.111 | 0.125 | 0.119 | 0.121 | 0.128 | 0.252 | 0.235 | 0.235 | 0.278 | 0.315 | 0.306 | 0.244 | 0.283 | 0.275 | 0.193 | 0.197 | 0.211 | 0.198 | 0.213 | 0.208 | 0.219 | 0.209 | 0.229 | **0.512** |  |  |  |
| Item25 | 0.136 | 0.156 | 0.152 | 0.156 | 0.156 | 0.223 | 0.217 | 0.219 | 0.329 | 0.377 | 0.329 | 0.309 | 0.303 | 0.300 | 0.182 | 0.191 | 0.218 | 0.309 | 0.328 | 0.252 | 0.228 | 0.224 | 0.264 | 0.355 | **0.696** |  |  |
| Item26 | 0.119 | 0.131 | 0.126 | 0.136 | 0.140 | 0.212 | 0.216 | 0.201 | 0.301 | 0.358 | 0.321 | 0.256 | 0.253 | 0.248 | 0.192 | 0.201 | 0.221 | 0.263 | 0.270 | 0.220 | 0.220 | 0.218 | 0.248 | 0.336 | 0.428 | **0.553** |  |
| Item27 | 0.120 | 0.129 | 0.124 | 0.138 | 0.136 | 0.226 | 0.222 | 0.223 | 0.282 | 0.326 | 0.296 | 0.249 | 0.260 | 0.257 | 0.196 | 0.207 | 0.233 | 0.253 | 0.255 | 0.215 | 0.222 | 0.224 | 0.246 | 0.343 | 0.397 | 0.383 | **0.564** |
| *Note*. The diagonal line is the variances, marked in **bold** | | | | | | | | | | | | | | | | | | | | | | | | | | | |

| **Table E.2** Item covariance matrix of the KIDSCREEN-27 in male sample of the baseline survey (*n* = 1921) | | | | | | | | | | | | | | | | | | | | | | | | | | | |
| --- | --- | --- | --- | --- | --- | --- | --- | --- | --- | --- | --- | --- | --- | --- | --- | --- | --- | --- | --- | --- | --- | --- | --- | --- | --- | --- | --- |
|  | **Item1** | **Item2** | **Item3** | **Item4** | **Item5** | **Item6** | **Item7** | **Item8** | **Item9** | **Item10** | **Item11** | **Item12** | **Item13** | **Item14** | **Item15** | **Item16** | **Item17** | **Item18** | **Item19** | **Item20** | **Item21** | **Item22** | **Item23** | **Item24** | **Item25** | **Item26** | **Item27** |
| Item1 | **0.388** |  |  |  |  |  |  |  |  |  |  |  |  |  |  |  |  |  |  |  |  |  |  |  |  |  |  |
| Item2 | 0.227 | **0.360** |  |  |  |  |  |  |  |  |  |  |  |  |  |  |  |  |  |  |  |  |  |  |  |  |  |
| Item3 | 0.220 | 0.233 | **0.474** |  |  |  |  |  |  |  |  |  |  |  |  |  |  |  |  |  |  |  |  |  |  |  |  |
| Item4 | 0.218 | 0.208 | 0.272 | **0.440** |  |  |  |  |  |  |  |  |  |  |  |  |  |  |  |  |  |  |  |  |  |  |  |
| Item5 | 0.221 | 0.211 | 0.228 | 0.202 | **0.408** |  |  |  |  |  |  |  |  |  |  |  |  |  |  |  |  |  |  |  |  |  |  |
| Item6 | 0.151 | 0.154 | 0.155 | 0.145 | 0.170 | **0.574** |  |  |  |  |  |  |  |  |  |  |  |  |  |  |  |  |  |  |  |  |  |
| Item7 | 0.149 | 0.138 | 0.143 | 0.139 | 0.176 | 0.418 | **0.627** |  |  |  |  |  |  |  |  |  |  |  |  |  |  |  |  |  |  |  |  |
| Item8 | 0.142 | 0.144 | 0.151 | 0.150 | 0.173 | 0.401 | 0.427 | **0.619** |  |  |  |  |  |  |  |  |  |  |  |  |  |  |  |  |  |  |  |
| Item9 | 0.171 | 0.152 | 0.151 | 0.154 | 0.168 | 0.322 | 0.346 | 0.329 | **0.865** |  |  |  |  |  |  |  |  |  |  |  |  |  |  |  |  |  |  |
| Item10 | 0.176 | 0.165 | 0.170 | 0.166 | 0.191 | 0.308 | 0.353 | 0.335 | 0.739 | **1.008** |  |  |  |  |  |  |  |  |  |  |  |  |  |  |  |  |  |
| Item11 | 0.184 | 0.166 | 0.177 | 0.169 | 0.175 | 0.356 | 0.372 | 0.363 | 0.667 | 0.728 | **0.935** |  |  |  |  |  |  |  |  |  |  |  |  |  |  |  |  |
| Item12 | 0.152 | 0.128 | 0.123 | 0.127 | 0.158 | 0.248 | 0.275 | 0.239 | 0.345 | 0.373 | 0.366 | **0.678** |  |  |  |  |  |  |  |  |  |  |  |  |  |  |  |
| Item13 | 0.132 | 0.133 | 0.147 | 0.160 | 0.137 | 0.286 | 0.275 | 0.272 | 0.332 | 0.365 | 0.349 | 0.286 | **1.044** |  |  |  |  |  |  |  |  |  |  |  |  |  |  |
| Item14 | 0.125 | 0.118 | 0.143 | 0.140 | 0.134 | 0.268 | 0.256 | 0.257 | 0.310 | 0.341 | 0.338 | 0.270 | 0.916 | **1.036** |  |  |  |  |  |  |  |  |  |  |  |  |  |
| Item15 | 0.098 | 0.078 | 0.095 | 0.084 | 0.114 | 0.234 | 0.241 | 0.236 | 0.240 | 0.261 | 0.268 | 0.217 | 0.256 | 0.251 | **0.709** |  |  |  |  |  |  |  |  |  |  |  |  |
| Item16 | 0.083 | 0.071 | 0.072 | 0.084 | 0.089 | 0.214 | 0.225 | 0.224 | 0.227 | 0.249 | 0.269 | 0.232 | 0.240 | 0.242 | 0.386 | **0.568** |  |  |  |  |  |  |  |  |  |  |  |
| Item17 | 0.118 | 0.102 | 0.108 | 0.099 | 0.141 | 0.253 | 0.262 | 0.253 | 0.273 | 0.307 | 0.321 | 0.260 | 0.277 | 0.277 | 0.482 | 0.405 | **0.725** |  |  |  |  |  |  |  |  |  |  |
| Item18 | 0.114 | 0.154 | 0.172 | 0.159 | 0.178 | 0.204 | 0.237 | 0.209 | 0.280 | 0.329 | 0.280 | 0.195 | 0.190 | 0.197 | 0.157 | 0.140 | 0.222 | **1.181** |  |  |  |  |  |  |  |  |  |
| Item19 | 0.107 | 0.141 | 0.152 | 0.163 | 0.156 | 0.195 | 0.215 | 0.201 | 0.274 | 0.338 | 0.291 | 0.216 | 0.214 | 0.227 | 0.143 | 0.182 | 0.200 | 0.877 | **1.066** |  |  |  |  |  |  |  |  |
| Item20 | 0.135 | 0.137 | 0.145 | 0.137 | 0.160 | 0.197 | 0.213 | 0.220 | 0.247 | 0.273 | 0.258 | 0.216 | 0.256 | 0.251 | 0.145 | 0.164 | 0.198 | 0.278 | 0.248 | **0.600** |  |  |  |  |  |  |  |
| Item21 | 0.121 | 0.116 | 0.120 | 0.126 | 0.135 | 0.208 | 0.224 | 0.225 | 0.264 | 0.280 | 0.282 | 0.208 | 0.217 | 0.202 | 0.170 | 0.184 | 0.208 | 0.208 | 0.198 | 0.341 | **0.488** |  |  |  |  |  |  |
| Item22 | 0.120 | 0.115 | 0.124 | 0.128 | 0.139 | 0.205 | 0.231 | 0.223 | 0.267 | 0.286 | 0.281 | 0.219 | 0.212 | 0.197 | 0.173 | 0.189 | 0.220 | 0.229 | 0.234 | 0.331 | 0.371 | **0.480** |  |  |  |  |  |
| Item23 | 0.129 | 0.132 | 0.120 | 0.138 | 0.150 | 0.202 | 0.227 | 0.229 | 0.294 | 0.314 | 0.315 | 0.235 | 0.221 | 0.221 | 0.154 | 0.197 | 0.221 | 0.264 | 0.281 | 0.336 | 0.347 | 0.352 | **0.563** |  |  |  |  |
| Item24 | 0.110 | 0.120 | 0.116 | 0.114 | 0.132 | 0.246 | 0.244 | 0.234 | 0.284 | 0.318 | 0.316 | 0.243 | 0.291 | 0.272 | 0.188 | 0.201 | 0.218 | 0.196 | 0.231 | 0.221 | 0.235 | 0.235 | 0.255 | **0.536** |  |  |  |
| Item25 | 0.138 | 0.162 | 0.151 | 0.161 | 0.170 | 0.241 | 0.264 | 0.254 | 0.350 | 0.394 | 0.352 | 0.321 | 0.309 | 0.302 | 0.192 | 0.208 | 0.231 | 0.342 | 0.373 | 0.278 | 0.260 | 0.262 | 0.308 | 0.388 | **0.734** |  |  |
| Item26 | 0.112 | 0.127 | 0.126 | 0.129 | 0.143 | 0.217 | 0.238 | 0.215 | 0.308 | 0.366 | 0.330 | 0.253 | 0.256 | 0.251 | 0.193 | 0.207 | 0.234 | 0.271 | 0.299 | 0.242 | 0.247 | 0.249 | 0.283 | 0.364 | 0.464 | **0.585** |  |
| Item27 | 0.108 | 0.126 | 0.126 | 0.137 | 0.144 | 0.237 | 0.245 | 0.237 | 0.286 | 0.340 | 0.307 | 0.260 | 0.268 | 0.263 | 0.192 | 0.207 | 0.241 | 0.259 | 0.288 | 0.222 | 0.234 | 0.245 | 0.275 | 0.377 | 0.434 | 0.418 | **0.609** |
| *Note*. The diagonal line is the variances, marked in **bold** | | | | | | | | | | | | | | | | | | | | | | | | | | | |

| **Table E.3** Item covariance matrix of the KIDSCREEN-27 in female sample of the baseline survey (*n* = 2193) | | | | | | | | | | | | | | | | | | | | | | | | | | | |
| --- | --- | --- | --- | --- | --- | --- | --- | --- | --- | --- | --- | --- | --- | --- | --- | --- | --- | --- | --- | --- | --- | --- | --- | --- | --- | --- | --- |
|  | **Item1** | **Item2** | **Item3** | **Item4** | **Item5** | **Item6** | **Item7** | **Item8** | **Item9** | **Item10** | **Item11** | **Item12** | **Item13** | **Item14** | **Item15** | **Item16** | **Item17** | **Item18** | **Item19** | **Item20** | **Item21** | **Item22** | **Item23** | **Item24** | **Item25** | **Item26** | **Item27** |
| Item1 | **0.393** |  |  |  |  |  |  |  |  |  |  |  |  |  |  |  |  |  |  |  |  |  |  |  |  |  |  |
| Item2 | 0.207 | **0.352** |  |  |  |  |  |  |  |  |  |  |  |  |  |  |  |  |  |  |  |  |  |  |  |  |  |
| Item3 | 0.199 | 0.215 | **0.463** |  |  |  |  |  |  |  |  |  |  |  |  |  |  |  |  |  |  |  |  |  |  |  |  |
| Item4 | 0.208 | 0.203 | 0.250 | **0.405** |  |  |  |  |  |  |  |  |  |  |  |  |  |  |  |  |  |  |  |  |  |  |  |
| Item5 | 0.206 | 0.187 | 0.204 | 0.195 | **0.381** |  |  |  |  |  |  |  |  |  |  |  |  |  |  |  |  |  |  |  |  |  |  |
| Item6 | 0.131 | 0.155 | 0.143 | 0.146 | 0.147 | **0.548** |  |  |  |  |  |  |  |  |  |  |  |  |  |  |  |  |  |  |  |  |  |
| Item7 | 0.123 | 0.139 | 0.132 | 0.133 | 0.150 | 0.378 | **0.556** |  |  |  |  |  |  |  |  |  |  |  |  |  |  |  |  |  |  |  |  |
| Item8 | 0.123 | 0.140 | 0.139 | 0.138 | 0.137 | 0.376 | 0.396 | **0.583** |  |  |  |  |  |  |  |  |  |  |  |  |  |  |  |  |  |  |  |
| Item9 | 0.163 | 0.172 | 0.148 | 0.150 | 0.155 | 0.313 | 0.283 | 0.276 | **0.854** |  |  |  |  |  |  |  |  |  |  |  |  |  |  |  |  |  |  |
| Item10 | 0.182 | 0.192 | 0.174 | 0.172 | 0.186 | 0.324 | 0.307 | 0.286 | 0.757 | **1.037** |  |  |  |  |  |  |  |  |  |  |  |  |  |  |  |  |  |
| Item11 | 0.173 | 0.177 | 0.166 | 0.171 | 0.177 | 0.319 | 0.291 | 0.302 | 0.659 | 0.716 | **0.945** |  |  |  |  |  |  |  |  |  |  |  |  |  |  |  |  |
| Item12 | 0.139 | 0.144 | 0.123 | 0.129 | 0.139 | 0.247 | 0.214 | 0.218 | 0.363 | 0.402 | 0.358 | **0.690** |  |  |  |  |  |  |  |  |  |  |  |  |  |  |  |
| Item13 | 0.115 | 0.148 | 0.133 | 0.133 | 0.123 | 0.278 | 0.231 | 0.253 | 0.309 | 0.343 | 0.327 | 0.292 | **0.977** |  |  |  |  |  |  |  |  |  |  |  |  |  |  |
| Item14 | 0.100 | 0.145 | 0.123 | 0.129 | 0.123 | 0.271 | 0.234 | 0.250 | 0.308 | 0.352 | 0.331 | 0.306 | 0.859 | **1.018** |  |  |  |  |  |  |  |  |  |  |  |  |  |
| Item15 | 0.121 | 0.101 | 0.115 | 0.119 | 0.131 | 0.253 | 0.237 | 0.238 | 0.228 | 0.258 | 0.264 | 0.196 | 0.237 | 0.252 | **0.720** |  |  |  |  |  |  |  |  |  |  |  |  |
| Item16 | 0.102 | 0.092 | 0.098 | 0.116 | 0.102 | 0.229 | 0.207 | 0.222 | 0.216 | 0.262 | 0.255 | 0.197 | 0.223 | 0.230 | 0.385 | **0.558** |  |  |  |  |  |  |  |  |  |  |  |
| Item17 | 0.125 | 0.102 | 0.118 | 0.116 | 0.136 | 0.251 | 0.232 | 0.244 | 0.262 | 0.295 | 0.282 | 0.217 | 0.272 | 0.292 | 0.478 | 0.409 | **0.724** |  |  |  |  |  |  |  |  |  |  |
| Item18 | 0.114 | 0.144 | 0.172 | 0.148 | 0.133 | 0.181 | 0.169 | 0.168 | 0.274 | 0.303 | 0.241 | 0.221 | 0.183 | 0.187 | 0.179 | 0.176 | 0.204 | **1.130** |  |  |  |  |  |  |  |  |  |
| Item19 | 0.097 | 0.139 | 0.133 | 0.134 | 0.106 | 0.162 | 0.170 | 0.170 | 0.254 | 0.299 | 0.236 | 0.223 | 0.203 | 0.206 | 0.142 | 0.187 | 0.182 | 0.792 | **0.953** |  |  |  |  |  |  |  |  |
| Item20 | 0.112 | 0.106 | 0.127 | 0.125 | 0.125 | 0.176 | 0.190 | 0.198 | 0.204 | 0.225 | 0.223 | 0.198 | 0.239 | 0.241 | 0.190 | 0.169 | 0.203 | 0.252 | 0.240 | **0.599** |  |  |  |  |  |  |  |
| Item21 | 0.099 | 0.101 | 0.112 | 0.121 | 0.114 | 0.173 | 0.183 | 0.212 | 0.208 | 0.220 | 0.239 | 0.180 | 0.195 | 0.194 | 0.184 | 0.179 | 0.185 | 0.211 | 0.198 | 0.322 | **0.449** |  |  |  |  |  |  |
| Item22 | 0.106 | 0.105 | 0.116 | 0.115 | 0.115 | 0.163 | 0.176 | 0.187 | 0.193 | 0.209 | 0.230 | 0.166 | 0.190 | 0.189 | 0.177 | 0.181 | 0.185 | 0.209 | 0.187 | 0.299 | 0.335 | **0.417** |  |  |  |  |  |
| Item23 | 0.118 | 0.117 | 0.123 | 0.135 | 0.115 | 0.182 | 0.175 | 0.199 | 0.222 | 0.248 | 0.267 | 0.214 | 0.211 | 0.220 | 0.168 | 0.196 | 0.191 | 0.227 | 0.211 | 0.303 | 0.287 | 0.288 | **0.483** |  |  |  |  |
| Item24 | 0.113 | 0.131 | 0.126 | 0.129 | 0.128 | 0.258 | 0.226 | 0.236 | 0.275 | 0.315 | 0.299 | 0.246 | 0.279 | 0.282 | 0.196 | 0.193 | 0.203 | 0.201 | 0.198 | 0.196 | 0.203 | 0.185 | 0.205 | **0.491** |  |  |  |
| Item25 | 0.134 | 0.151 | 0.153 | 0.152 | 0.144 | 0.208 | 0.176 | 0.189 | 0.311 | 0.363 | 0.310 | 0.299 | 0.298 | 0.299 | 0.173 | 0.177 | 0.205 | 0.280 | 0.288 | 0.229 | 0.199 | 0.191 | 0.226 | 0.325 | **0.663** |  |  |
| Item26 | 0.126 | 0.137 | 0.130 | 0.145 | 0.140 | 0.208 | 0.196 | 0.188 | 0.296 | 0.353 | 0.315 | 0.260 | 0.254 | 0.250 | 0.191 | 0.196 | 0.209 | 0.256 | 0.245 | 0.200 | 0.195 | 0.190 | 0.216 | 0.310 | 0.397 | **0.524** |  |
| Item27 | 0.133 | 0.137 | 0.131 | 0.146 | 0.135 | 0.218 | 0.201 | 0.209 | 0.282 | 0.318 | 0.291 | 0.244 | 0.262 | 0.259 | 0.199 | 0.208 | 0.224 | 0.249 | 0.225 | 0.209 | 0.210 | 0.202 | 0.218 | 0.312 | 0.364 | 0.351 | **0.522** |
| *Note*. The diagonal line is the variances, marked in **bold** | | | | | | | | | | | | | | | | | | | | | | | | | | | |

| **Table E.4** Item covariance matrix of the KIDSCREEN-27 in primary school sample of the baseline survey (*n* = 912) | | | | | | | | | | | | | | | | | | | | | | | | | | | |
| --- | --- | --- | --- | --- | --- | --- | --- | --- | --- | --- | --- | --- | --- | --- | --- | --- | --- | --- | --- | --- | --- | --- | --- | --- | --- | --- | --- |
|  | **Item1** | **Item2** | **Item3** | **Item4** | **Item5** | **Item6** | **Item7** | **Item8** | **Item9** | **Item10** | **Item11** | **Item12** | **Item13** | **Item14** | **Item15** | **Item16** | **Item17** | **Item18** | **Item19** | **Item20** | **Item21** | **Item22** | **Item23** | **Item24** | **Item25** | **Item26** | **Item27** |
| Item1 | **0.202** |  |  |  |  |  |  |  |  |  |  |  |  |  |  |  |  |  |  |  |  |  |  |  |  |  |  |
| Item2 | 0.083 | **0.250** |  |  |  |  |  |  |  |  |  |  |  |  |  |  |  |  |  |  |  |  |  |  |  |  |  |
| Item3 | 0.083 | 0.090 | **0.446** |  |  |  |  |  |  |  |  |  |  |  |  |  |  |  |  |  |  |  |  |  |  |  |  |
| Item4 | 0.078 | 0.062 | 0.147 | **0.335** |  |  |  |  |  |  |  |  |  |  |  |  |  |  |  |  |  |  |  |  |  |  |  |
| Item5 | 0.081 | 0.083 | 0.112 | 0.055 | **0.255** |  |  |  |  |  |  |  |  |  |  |  |  |  |  |  |  |  |  |  |  |  |  |
| Item6 | 0.056 | 0.093 | 0.103 | 0.077 | 0.077 | **0.464** |  |  |  |  |  |  |  |  |  |  |  |  |  |  |  |  |  |  |  |  |  |
| Item7 | 0.053 | 0.086 | 0.077 | 0.070 | 0.072 | 0.326 | **0.516** |  |  |  |  |  |  |  |  |  |  |  |  |  |  |  |  |  |  |  |  |
| Item8 | 0.047 | 0.079 | 0.097 | 0.089 | 0.071 | 0.309 | 0.304 | **0.518** |  |  |  |  |  |  |  |  |  |  |  |  |  |  |  |  |  |  |  |
| Item9 | 0.086 | 0.087 | 0.084 | 0.082 | 0.073 | 0.293 | 0.280 | 0.262 | **0.711** |  |  |  |  |  |  |  |  |  |  |  |  |  |  |  |  |  |  |
| Item10 | 0.073 | 0.090 | 0.119 | 0.087 | 0.086 | 0.281 | 0.259 | 0.257 | 0.560 | **0.746** |  |  |  |  |  |  |  |  |  |  |  |  |  |  |  |  |  |
| Item11 | 0.076 | 0.078 | 0.112 | 0.079 | 0.074 | 0.271 | 0.278 | 0.253 | 0.502 | 0.548 | **0.724** |  |  |  |  |  |  |  |  |  |  |  |  |  |  |  |  |
| Item12 | 0.066 | 0.080 | 0.064 | 0.066 | 0.077 | 0.177 | 0.198 | 0.156 | 0.223 | 0.228 | 0.225 | **0.466** |  |  |  |  |  |  |  |  |  |  |  |  |  |  |  |
| Item13 | 0.062 | 0.092 | 0.119 | 0.105 | 0.085 | 0.241 | 0.239 | 0.205 | 0.269 | 0.294 | 0.284 | 0.206 | **0.784** |  |  |  |  |  |  |  |  |  |  |  |  |  |  |
| Item14 | 0.058 | 0.090 | 0.125 | 0.099 | 0.091 | 0.228 | 0.237 | 0.203 | 0.266 | 0.296 | 0.302 | 0.198 | 0.614 | **0.726** |  |  |  |  |  |  |  |  |  |  |  |  |  |
| Item15 | 0.065 | 0.054 | 0.061 | 0.053 | 0.070 | 0.168 | 0.189 | 0.182 | 0.192 | 0.222 | 0.213 | 0.154 | 0.204 | 0.186 | **0.547** |  |  |  |  |  |  |  |  |  |  |  |  |
| Item16 | 0.034 | 0.043 | 0.061 | 0.076 | 0.032 | 0.177 | 0.176 | 0.190 | 0.187 | 0.206 | 0.195 | 0.155 | 0.219 | 0.208 | 0.228 | **0.433** |  |  |  |  |  |  |  |  |  |  |  |
| Item17 | 0.054 | 0.068 | 0.075 | 0.063 | 0.069 | 0.172 | 0.170 | 0.180 | 0.218 | 0.232 | 0.215 | 0.168 | 0.222 | 0.219 | 0.300 | 0.221 | **0.524** |  |  |  |  |  |  |  |  |  |  |
| Item18 | 0.050 | 0.133 | 0.168 | 0.136 | 0.094 | 0.305 | 0.308 | 0.274 | 0.364 | 0.379 | 0.323 | 0.230 | 0.275 | 0.308 | 0.185 | 0.201 | 0.242 | **1.352** |  |  |  |  |  |  |  |  |  |
| Item19 | 0.038 | 0.088 | 0.146 | 0.148 | 0.078 | 0.245 | 0.245 | 0.237 | 0.291 | 0.294 | 0.263 | 0.187 | 0.230 | 0.262 | 0.165 | 0.188 | 0.198 | 0.849 | **0.866** |  |  |  |  |  |  |  |  |
| Item20 | 0.066 | 0.089 | 0.081 | 0.082 | 0.081 | 0.187 | 0.191 | 0.173 | 0.208 | 0.215 | 0.192 | 0.162 | 0.234 | 0.227 | 0.128 | 0.125 | 0.146 | 0.326 | 0.262 | **0.482** |  |  |  |  |  |  |  |
| Item21 | 0.066 | 0.078 | 0.076 | 0.086 | 0.076 | 0.191 | 0.184 | 0.195 | 0.239 | 0.246 | 0.217 | 0.161 | 0.233 | 0.226 | 0.146 | 0.149 | 0.148 | 0.303 | 0.256 | 0.267 | **0.398** |  |  |  |  |  |  |
| Item22 | 0.052 | 0.075 | 0.076 | 0.082 | 0.062 | 0.189 | 0.191 | 0.191 | 0.240 | 0.237 | 0.220 | 0.167 | 0.232 | 0.230 | 0.145 | 0.140 | 0.158 | 0.325 | 0.276 | 0.258 | 0.298 | **0.400** |  |  |  |  |  |
| Item23 | 0.028 | 0.059 | 0.061 | 0.080 | 0.040 | 0.177 | 0.175 | 0.176 | 0.221 | 0.223 | 0.216 | 0.162 | 0.204 | 0.215 | 0.112 | 0.168 | 0.145 | 0.338 | 0.274 | 0.227 | 0.241 | 0.248 | **0.399** |  |  |  |  |
| Item24 | 0.051 | 0.083 | 0.068 | 0.072 | 0.068 | 0.198 | 0.186 | 0.165 | 0.205 | 0.199 | 0.194 | 0.161 | 0.223 | 0.221 | 0.156 | 0.158 | 0.151 | 0.259 | 0.201 | 0.186 | 0.210 | 0.208 | 0.185 | **0.466** |  |  |  |
| Item25 | 0.053 | 0.095 | 0.110 | 0.098 | 0.067 | 0.227 | 0.209 | 0.212 | 0.272 | 0.278 | 0.242 | 0.187 | 0.244 | 0.257 | 0.152 | 0.190 | 0.177 | 0.406 | 0.336 | 0.216 | 0.222 | 0.225 | 0.212 | 0.277 | **0.591** |  |  |
| Item26 | 0.048 | 0.069 | 0.072 | 0.089 | 0.058 | 0.204 | 0.187 | 0.183 | 0.249 | 0.256 | 0.236 | 0.163 | 0.197 | 0.206 | 0.152 | 0.170 | 0.160 | 0.299 | 0.243 | 0.197 | 0.227 | 0.218 | 0.209 | 0.285 | 0.338 | **0.379** |  |
| Item27 | 0.053 | 0.081 | 0.084 | 0.083 | 0.056 | 0.186 | 0.182 | 0.173 | 0.240 | 0.229 | 0.213 | 0.145 | 0.193 | 0.200 | 0.151 | 0.175 | 0.152 | 0.332 | 0.263 | 0.185 | 0.210 | 0.216 | 0.203 | 0.279 | 0.321 | 0.274 | **0.452** |
| *Note*. The diagonal line is the variances, marked in **bold** | | | | | | | | | | | | | | | | | | | | | | | | | | | |

| **Table E.5** Item covariance matrix of the KIDSCREEN-27 in junior secondary school sample of the baseline survey (*n* = 2022) | | | | | | | | | | | | | | | | | | | | | | | | | | | |
| --- | --- | --- | --- | --- | --- | --- | --- | --- | --- | --- | --- | --- | --- | --- | --- | --- | --- | --- | --- | --- | --- | --- | --- | --- | --- | --- | --- |
|  | **Item1** | **Item2** | **Item3** | **Item4** | **Item5** | **Item6** | **Item7** | **Item8** | **Item9** | **Item10** | **Item11** | **Item12** | **Item13** | **Item14** | **Item15** | **Item16** | **Item17** | **Item18** | **Item19** | **Item20** | **Item21** | **Item22** | **Item23** | **Item24** | **Item25** | **Item26** | **Item27** |
| Item1 | **0.505** |  |  |  |  |  |  |  |  |  |  |  |  |  |  |  |  |  |  |  |  |  |  |  |  |  |  |
| Item2 | 0.299 | **0.395** |  |  |  |  |  |  |  |  |  |  |  |  |  |  |  |  |  |  |  |  |  |  |  |  |  |
| Item3 | 0.294 | 0.294 | **0.508** |  |  |  |  |  |  |  |  |  |  |  |  |  |  |  |  |  |  |  |  |  |  |  |  |
| Item4 | 0.295 | 0.282 | 0.341 | **0.497** |  |  |  |  |  |  |  |  |  |  |  |  |  |  |  |  |  |  |  |  |  |  |  |
| Item5 | 0.299 | 0.271 | 0.307 | 0.297 | **0.496** |  |  |  |  |  |  |  |  |  |  |  |  |  |  |  |  |  |  |  |  |  |  |
| Item6 | 0.188 | 0.174 | 0.172 | 0.166 | 0.212 | **0.595** |  |  |  |  |  |  |  |  |  |  |  |  |  |  |  |  |  |  |  |  |  |
| Item7 | 0.182 | 0.164 | 0.167 | 0.159 | 0.219 | 0.424 | **0.627** |  |  |  |  |  |  |  |  |  |  |  |  |  |  |  |  |  |  |  |  |
| Item8 | 0.174 | 0.162 | 0.168 | 0.160 | 0.197 | 0.420 | 0.457 | **0.635** |  |  |  |  |  |  |  |  |  |  |  |  |  |  |  |  |  |  |  |
| Item9 | 0.201 | 0.174 | 0.166 | 0.162 | 0.200 | 0.330 | 0.323 | 0.320 | **0.876** |  |  |  |  |  |  |  |  |  |  |  |  |  |  |  |  |  |  |
| Item10 | 0.220 | 0.189 | 0.182 | 0.183 | 0.235 | 0.321 | 0.350 | 0.324 | 0.739 | **1.009** |  |  |  |  |  |  |  |  |  |  |  |  |  |  |  |  |  |
| Item11 | 0.220 | 0.186 | 0.190 | 0.189 | 0.226 | 0.368 | 0.363 | 0.381 | 0.673 | 0.708 | **0.972** |  |  |  |  |  |  |  |  |  |  |  |  |  |  |  |  |
| Item12 | 0.161 | 0.133 | 0.122 | 0.127 | 0.166 | 0.261 | 0.249 | 0.240 | 0.347 | 0.362 | 0.365 | **0.695** |  |  |  |  |  |  |  |  |  |  |  |  |  |  |  |
| Item13 | 0.162 | 0.153 | 0.162 | 0.158 | 0.162 | 0.291 | 0.243 | 0.274 | 0.303 | 0.328 | 0.341 | 0.271 | **1.036** |  |  |  |  |  |  |  |  |  |  |  |  |  |  |
| Item14 | 0.142 | 0.135 | 0.147 | 0.143 | 0.151 | 0.273 | 0.226 | 0.259 | 0.287 | 0.322 | 0.333 | 0.265 | 0.917 | **1.047** |  |  |  |  |  |  |  |  |  |  |  |  |  |
| Item15 | 0.147 | 0.121 | 0.150 | 0.134 | 0.168 | 0.300 | 0.288 | 0.289 | 0.282 | 0.309 | 0.333 | 0.239 | 0.286 | 0.285 | **0.819** |  |  |  |  |  |  |  |  |  |  |  |  |
| Item16 | 0.126 | 0.099 | 0.106 | 0.112 | 0.133 | 0.266 | 0.254 | 0.259 | 0.257 | 0.300 | 0.320 | 0.240 | 0.268 | 0.269 | 0.470 | **0.670** |  |  |  |  |  |  |  |  |  |  |  |
| Item17 | 0.175 | 0.143 | 0.161 | 0.139 | 0.196 | 0.333 | 0.320 | 0.320 | 0.338 | 0.373 | 0.395 | 0.269 | 0.343 | 0.345 | 0.605 | 0.530 | **0.882** |  |  |  |  |  |  |  |  |  |  |
| Item18 | 0.118 | 0.104 | 0.137 | 0.130 | 0.144 | 0.116 | 0.150 | 0.123 | 0.169 | 0.185 | 0.142 | 0.109 | 0.084 | 0.077 | 0.169 | 0.144 | 0.194 | **1.000** |  |  |  |  |  |  |  |  |  |
| Item19 | 0.101 | 0.093 | 0.085 | 0.106 | 0.108 | 0.111 | 0.143 | 0.117 | 0.149 | 0.197 | 0.148 | 0.112 | 0.091 | 0.091 | 0.114 | 0.187 | 0.156 | 0.727 | **0.966** |  |  |  |  |  |  |  |  |
| Item20 | 0.145 | 0.127 | 0.155 | 0.141 | 0.166 | 0.188 | 0.203 | 0.218 | 0.205 | 0.232 | 0.221 | 0.170 | 0.235 | 0.226 | 0.189 | 0.181 | 0.212 | 0.163 | 0.157 | **0.624** |  |  |  |  |  |  |  |
| Item21 | 0.126 | 0.108 | 0.125 | 0.129 | 0.146 | 0.192 | 0.210 | 0.227 | 0.218 | 0.233 | 0.263 | 0.166 | 0.172 | 0.162 | 0.193 | 0.192 | 0.222 | 0.146 | 0.136 | 0.344 | **0.495** |  |  |  |  |  |  |
| Item22 | 0.134 | 0.114 | 0.138 | 0.129 | 0.153 | 0.187 | 0.216 | 0.217 | 0.225 | 0.252 | 0.270 | 0.178 | 0.177 | 0.164 | 0.193 | 0.209 | 0.229 | 0.147 | 0.152 | 0.331 | 0.383 | **0.490** |  |  |  |  |  |
| Item23 | 0.165 | 0.142 | 0.144 | 0.153 | 0.169 | 0.209 | 0.222 | 0.238 | 0.254 | 0.283 | 0.300 | 0.200 | 0.193 | 0.187 | 0.199 | 0.231 | 0.246 | 0.160 | 0.182 | 0.329 | 0.342 | 0.357 | **0.574** |  |  |  |  |
| Item24 | 0.135 | 0.118 | 0.123 | 0.125 | 0.155 | 0.258 | 0.242 | 0.247 | 0.266 | 0.302 | 0.314 | 0.221 | 0.242 | 0.229 | 0.221 | 0.227 | 0.271 | 0.142 | 0.155 | 0.207 | 0.219 | 0.222 | 0.241 | **0.488** |  |  |  |
| Item25 | 0.165 | 0.147 | 0.153 | 0.149 | 0.176 | 0.226 | 0.235 | 0.219 | 0.292 | 0.337 | 0.300 | 0.267 | 0.230 | 0.218 | 0.222 | 0.220 | 0.261 | 0.194 | 0.209 | 0.227 | 0.211 | 0.218 | 0.247 | 0.349 | **0.662** |  |  |
| Item26 | 0.147 | 0.138 | 0.141 | 0.142 | 0.169 | 0.216 | 0.238 | 0.203 | 0.282 | 0.343 | 0.315 | 0.208 | 0.203 | 0.196 | 0.237 | 0.236 | 0.277 | 0.204 | 0.204 | 0.209 | 0.203 | 0.220 | 0.247 | 0.336 | 0.411 | **0.575** |  |
| Item27 | 0.145 | 0.131 | 0.136 | 0.152 | 0.167 | 0.255 | 0.254 | 0.245 | 0.273 | 0.330 | 0.315 | 0.228 | 0.234 | 0.233 | 0.253 | 0.255 | 0.304 | 0.193 | 0.198 | 0.211 | 0.224 | 0.237 | 0.256 | 0.364 | 0.391 | 0.401 | **0.593** |
| *Note*. The diagonal line is the variances, marked in **bold** | | | | | | | | | | | | | | | | | | | | | | | | | | | |

| **Table E.6** Item covariance matrix of the KIDSCREEN-27 in senior secondary school sample of the baseline survey (*n* = 1180) | | | | | | | | | | | | | | | | | | | | | | | | | | | |
| --- | --- | --- | --- | --- | --- | --- | --- | --- | --- | --- | --- | --- | --- | --- | --- | --- | --- | --- | --- | --- | --- | --- | --- | --- | --- | --- | --- |
|  | **Item1** | **Item2** | **Item3** | **Item4** | **Item5** | **Item6** | **Item7** | **Item8** | **Item9** | **Item10** | **Item11** | **Item12** | **Item13** | **Item14** | **Item15** | **Item16** | **Item17** | **Item18** | **Item19** | **Item20** | **Item21** | **Item22** | **Item23** | **Item24** | **Item25** | **Item26** | **Item27** |
| Item1 | **0.338** |  |  |  |  |  |  |  |  |  |  |  |  |  |  |  |  |  |  |  |  |  |  |  |  |  |  |
| Item2 | 0.175 | **0.347** |  |  |  |  |  |  |  |  |  |  |  |  |  |  |  |  |  |  |  |  |  |  |  |  |  |
| Item3 | 0.169 | 0.205 | **0.440** |  |  |  |  |  |  |  |  |  |  |  |  |  |  |  |  |  |  |  |  |  |  |  |  |
| Item4 | 0.175 | 0.168 | 0.220 | **0.352** |  |  |  |  |  |  |  |  |  |  |  |  |  |  |  |  |  |  |  |  |  |  |  |
| Item5 | 0.168 | 0.162 | 0.159 | 0.144 | **0.326** |  |  |  |  |  |  |  |  |  |  |  |  |  |  |  |  |  |  |  |  |  |  |
| Item6 | 0.121 | 0.146 | 0.122 | 0.144 | 0.128 | **0.546** |  |  |  |  |  |  |  |  |  |  |  |  |  |  |  |  |  |  |  |  |  |
| Item7 | 0.110 | 0.114 | 0.109 | 0.126 | 0.125 | 0.387 | **0.571** |  |  |  |  |  |  |  |  |  |  |  |  |  |  |  |  |  |  |  |  |
| Item8 | 0.117 | 0.127 | 0.116 | 0.132 | 0.134 | 0.370 | 0.400 | **0.585** |  |  |  |  |  |  |  |  |  |  |  |  |  |  |  |  |  |  |  |
| Item9 | 0.149 | 0.142 | 0.141 | 0.139 | 0.140 | 0.272 | 0.285 | 0.250 | **0.825** |  |  |  |  |  |  |  |  |  |  |  |  |  |  |  |  |  |  |
| Item10 | 0.165 | 0.155 | 0.157 | 0.143 | 0.156 | 0.281 | 0.299 | 0.263 | 0.755 | **1.061** |  |  |  |  |  |  |  |  |  |  |  |  |  |  |  |  |  |
| Item11 | 0.168 | 0.163 | 0.156 | 0.161 | 0.153 | 0.285 | 0.271 | 0.252 | 0.652 | 0.725 | **0.935** |  |  |  |  |  |  |  |  |  |  |  |  |  |  |  |  |
| Item12 | 0.152 | 0.119 | 0.146 | 0.121 | 0.138 | 0.239 | 0.229 | 0.211 | 0.326 | 0.374 | 0.325 | **0.656** |  |  |  |  |  |  |  |  |  |  |  |  |  |  |  |
| Item13 | 0.085 | 0.079 | 0.081 | 0.094 | 0.098 | 0.221 | 0.215 | 0.206 | 0.216 | 0.220 | 0.200 | 0.196 | **0.880** |  |  |  |  |  |  |  |  |  |  |  |  |  |  |
| Item14 | 0.076 | 0.078 | 0.071 | 0.079 | 0.096 | 0.224 | 0.223 | 0.208 | 0.206 | 0.203 | 0.188 | 0.202 | 0.786 | **0.967** |  |  |  |  |  |  |  |  |  |  |  |  |  |
| Item15 | 0.080 | 0.063 | 0.062 | 0.083 | 0.082 | 0.206 | 0.193 | 0.190 | 0.176 | 0.194 | 0.186 | 0.180 | 0.201 | 0.237 | **0.666** |  |  |  |  |  |  |  |  |  |  |  |  |
| Item16 | 0.072 | 0.061 | 0.056 | 0.079 | 0.066 | 0.172 | 0.173 | 0.174 | 0.144 | 0.164 | 0.172 | 0.156 | 0.119 | 0.140 | 0.359 | **0.462** |  |  |  |  |  |  |  |  |  |  |  |
| Item17 | 0.067 | 0.045 | 0.055 | 0.073 | 0.066 | 0.189 | 0.184 | 0.183 | 0.159 | 0.196 | 0.185 | 0.186 | 0.179 | 0.208 | 0.400 | 0.324 | **0.576** |  |  |  |  |  |  |  |  |  |  |
| Item18 | 0.129 | 0.191 | 0.213 | 0.163 | 0.178 | 0.224 | 0.191 | 0.206 | 0.303 | 0.370 | 0.326 | 0.235 | 0.179 | 0.177 | 0.149 | 0.114 | 0.173 | **1.169** |  |  |  |  |  |  |  |  |  |
| Item19 | 0.111 | 0.175 | 0.180 | 0.136 | 0.151 | 0.196 | 0.197 | 0.208 | 0.276 | 0.330 | 0.296 | 0.221 | 0.162 | 0.160 | 0.164 | 0.119 | 0.195 | 0.860 | **0.961** |  |  |  |  |  |  |  |  |
| Item20 | 0.105 | 0.098 | 0.130 | 0.115 | 0.109 | 0.180 | 0.195 | 0.205 | 0.198 | 0.206 | 0.243 | 0.196 | 0.198 | 0.201 | 0.160 | 0.140 | 0.173 | 0.301 | 0.263 | **0.564** |  |  |  |  |  |  |  |
| Item21 | 0.091 | 0.092 | 0.102 | 0.102 | 0.089 | 0.174 | 0.193 | 0.205 | 0.188 | 0.185 | 0.218 | 0.169 | 0.147 | 0.139 | 0.169 | 0.163 | 0.160 | 0.180 | 0.163 | 0.298 | **0.430** |  |  |  |  |  |  |
| Item22 | 0.100 | 0.096 | 0.098 | 0.103 | 0.096 | 0.166 | 0.181 | 0.181 | 0.166 | 0.166 | 0.199 | 0.148 | 0.142 | 0.136 | 0.164 | 0.156 | 0.159 | 0.198 | 0.174 | 0.271 | 0.303 | **0.376** |  |  |  |  |  |
| Item23 | 0.100 | 0.097 | 0.102 | 0.103 | 0.097 | 0.160 | 0.168 | 0.178 | 0.199 | 0.200 | 0.242 | 0.189 | 0.148 | 0.160 | 0.128 | 0.124 | 0.142 | 0.228 | 0.199 | 0.290 | 0.268 | 0.251 | **0.444** |  |  |  |  |
| Item24 | 0.093 | 0.101 | 0.101 | 0.092 | 0.108 | 0.230 | 0.224 | 0.220 | 0.238 | 0.275 | 0.255 | 0.213 | 0.210 | 0.212 | 0.168 | 0.140 | 0.153 | 0.178 | 0.181 | 0.180 | 0.173 | 0.154 | 0.178 | **0.467** |  |  |  |
| Item25 | 0.104 | 0.113 | 0.115 | 0.116 | 0.128 | 0.159 | 0.147 | 0.158 | 0.237 | 0.265 | 0.252 | 0.223 | 0.189 | 0.185 | 0.124 | 0.073 | 0.123 | 0.267 | 0.254 | 0.187 | 0.144 | 0.139 | 0.184 | 0.246 | **0.504** |  |  |
| Item26 | 0.093 | 0.087 | 0.088 | 0.091 | 0.110 | 0.161 | 0.162 | 0.158 | 0.224 | 0.270 | 0.248 | 0.227 | 0.163 | 0.152 | 0.139 | 0.114 | 0.143 | 0.222 | 0.211 | 0.165 | 0.161 | 0.151 | 0.173 | 0.236 | 0.287 | **0.472** |  |
| Item27 | 0.099 | 0.099 | 0.094 | 0.098 | 0.106 | 0.176 | 0.172 | 0.184 | 0.208 | 0.235 | 0.208 | 0.210 | 0.186 | 0.168 | 0.125 | 0.107 | 0.138 | 0.192 | 0.179 | 0.158 | 0.155 | 0.147 | 0.165 | 0.250 | 0.262 | 0.290 | **0.476** |
| *Note*. The diagonal line is the variances, marked in **bold** | | | | | | | | | | | | | | | | | | | | | | | | | | | |

| **Table F.1** Item covariance matrix of the KIDSCREEN-27 in the retest survey (*N* = 789) | | | | | | | | | | | | | | | | | | | | | | | | | | | |
| --- | --- | --- | --- | --- | --- | --- | --- | --- | --- | --- | --- | --- | --- | --- | --- | --- | --- | --- | --- | --- | --- | --- | --- | --- | --- | --- | --- |
|  | **Item1** | **Item2** | **Item3** | **Item4** | **Item5** | **Item6** | **Item7** | **Item8** | **Item9** | **Item10** | **Item11** | **Item12** | **Item13** | **Item14** | **Item15** | **Item16** | **Item17** | **Item18** | **Item19** | **Item20** | **Item21** | **Item22** | **Item23** | **Item24** | **Item25** | **Item26** | **Item27** |
| Item1 | **0.391** |  |  |  |  |  |  |  |  |  |  |  |  |  |  |  |  |  |  |  |  |  |  |  |  |  |  |
| Item2 | 0.120 | **0.362** |  |  |  |  |  |  |  |  |  |  |  |  |  |  |  |  |  |  |  |  |  |  |  |  |  |
| Item3 | 0.139 | 0.123 | **0.367** |  |  |  |  |  |  |  |  |  |  |  |  |  |  |  |  |  |  |  |  |  |  |  |  |
| Item4 | 0.115 | 0.122 | 0.103 | **0.330** |  |  |  |  |  |  |  |  |  |  |  |  |  |  |  |  |  |  |  |  |  |  |  |
| Item5 | 0.121 | 0.092 | 0.102 | 0.083 | **0.363** |  |  |  |  |  |  |  |  |  |  |  |  |  |  |  |  |  |  |  |  |  |  |
| Item6 | 0.063 | 0.044 | 0.025 | 0.029 | 0.051 | **0.407** |  |  |  |  |  |  |  |  |  |  |  |  |  |  |  |  |  |  |  |  |  |
| Item7 | 0.079 | 0.063 | 0.042 | 0.059 | 0.083 | 0.194 | **0.636** |  |  |  |  |  |  |  |  |  |  |  |  |  |  |  |  |  |  |  |  |
| Item8 | 0.096 | 0.060 | 0.064 | 0.044 | 0.083 | 0.146 | 0.190 | **0.515** |  |  |  |  |  |  |  |  |  |  |  |  |  |  |  |  |  |  |  |
| Item9 | 0.116 | 0.083 | 0.061 | 0.079 | 0.093 | 0.154 | 0.195 | 0.149 | **0.832** |  |  |  |  |  |  |  |  |  |  |  |  |  |  |  |  |  |  |
| Item10 | 0.133 | 0.113 | 0.092 | 0.087 | 0.097 | 0.147 | 0.239 | 0.169 | 0.441 | **0.874** |  |  |  |  |  |  |  |  |  |  |  |  |  |  |  |  |  |
| Item11 | 0.096 | 0.067 | 0.080 | 0.061 | 0.088 | 0.149 | 0.155 | 0.137 | 0.290 | 0.299 | **0.672** |  |  |  |  |  |  |  |  |  |  |  |  |  |  |  |  |
| Item12 | 0.112 | 0.064 | 0.078 | 0.086 | 0.101 | 0.134 | 0.129 | 0.136 | 0.228 | 0.192 | 0.206 | **0.903** |  |  |  |  |  |  |  |  |  |  |  |  |  |  |  |
| Item13 | 0.075 | 0.071 | 0.023 | 0.025 | 0.059 | 0.134 | 0.093 | 0.140 | 0.100 | 0.144 | 0.147 | 0.206 | **0.873** |  |  |  |  |  |  |  |  |  |  |  |  |  |  |
| Item14 | 0.055 | 0.092 | 0.037 | 0.037 | 0.040 | 0.146 | 0.106 | 0.137 | 0.167 | 0.170 | 0.193 | 0.205 | 0.527 | **1.058** |  |  |  |  |  |  |  |  |  |  |  |  |  |
| Item15 | 0.070 | 0.052 | 0.030 | 0.042 | 0.063 | 0.121 | 0.104 | 0.085 | 0.103 | 0.092 | 0.108 | 0.107 | 0.127 | 0.155 | **0.546** |  |  |  |  |  |  |  |  |  |  |  |  |
| Item16 | 0.064 | 0.031 | 0.015 | 0.052 | 0.057 | 0.095 | 0.095 | 0.072 | 0.115 | 0.111 | 0.117 | 0.094 | 0.098 | 0.100 | 0.179 | **0.489** |  |  |  |  |  |  |  |  |  |  |  |
| Item17 | 0.092 | 0.048 | 0.071 | 0.081 | 0.063 | 0.121 | 0.110 | 0.081 | 0.083 | 0.120 | 0.094 | 0.117 | 0.115 | 0.116 | 0.181 | 0.188 | **0.632** |  |  |  |  |  |  |  |  |  |  |
| Item18 | 0.072 | 0.131 | 0.092 | 0.078 | 0.068 | 0.120 | 0.140 | 0.121 | 0.201 | 0.195 | 0.160 | 0.123 | 0.153 | 0.179 | 0.106 | 0.107 | 0.170 | **1.032** |  |  |  |  |  |  |  |  |  |
| Item19 | 0.064 | 0.096 | 0.057 | 0.053 | 0.058 | 0.083 | 0.081 | 0.077 | 0.127 | 0.134 | 0.144 | 0.095 | 0.126 | 0.139 | 0.073 | 0.089 | 0.110 | 0.448 | **0.628** |  |  |  |  |  |  |  |  |
| Item20 | 0.082 | 0.063 | 0.036 | 0.046 | 0.063 | 0.067 | 0.095 | 0.066 | 0.143 | 0.129 | 0.111 | 0.100 | 0.129 | 0.123 | 0.060 | 0.077 | 0.075 | 0.169 | 0.119 | **0.558** |  |  |  |  |  |  |  |
| Item21 | 0.066 | 0.050 | 0.044 | 0.040 | 0.037 | 0.084 | 0.102 | 0.084 | 0.140 | 0.139 | 0.130 | 0.089 | 0.084 | 0.103 | 0.072 | 0.075 | 0.057 | 0.110 | 0.069 | 0.188 | **0.463** |  |  |  |  |  |  |
| Item22 | 0.078 | 0.068 | 0.037 | 0.048 | 0.056 | 0.079 | 0.079 | 0.090 | 0.130 | 0.128 | 0.103 | 0.093 | 0.071 | 0.078 | 0.073 | 0.098 | 0.056 | 0.125 | 0.084 | 0.188 | 0.180 | **0.418** |  |  |  |  |  |
| Item23 | 0.077 | 0.055 | 0.047 | 0.063 | 0.048 | 0.080 | 0.119 | 0.093 | 0.119 | 0.132 | 0.129 | 0.110 | 0.096 | 0.121 | 0.059 | 0.060 | 0.071 | 0.116 | 0.072 | 0.173 | 0.178 | 0.199 | **0.447** |  |  |  |  |
| Item24 | 0.099 | 0.075 | 0.057 | 0.056 | 0.084 | 0.130 | 0.151 | 0.152 | 0.223 | 0.232 | 0.198 | 0.178 | 0.207 | 0.241 | 0.119 | 0.104 | 0.119 | 0.190 | 0.137 | 0.162 | 0.117 | 0.108 | 0.120 | **0.603** |  |  |  |
| Item25 | 0.104 | 0.088 | 0.073 | 0.067 | 0.087 | 0.106 | 0.120 | 0.124 | 0.227 | 0.187 | 0.180 | 0.193 | 0.188 | 0.159 | 0.123 | 0.108 | 0.121 | 0.239 | 0.155 | 0.160 | 0.113 | 0.126 | 0.116 | 0.337 | **0.641** |  |  |
| Item26 | 0.107 | 0.096 | 0.109 | 0.057 | 0.107 | 0.118 | 0.108 | 0.125 | 0.226 | 0.226 | 0.219 | 0.222 | 0.182 | 0.182 | 0.131 | 0.111 | 0.125 | 0.237 | 0.164 | 0.154 | 0.156 | 0.138 | 0.159 | 0.285 | 0.285 | **0.820** |  |
| Item27 | 0.093 | 0.081 | 0.057 | 0.056 | 0.055 | 0.130 | 0.125 | 0.129 | 0.168 | 0.181 | 0.150 | 0.154 | 0.156 | 0.173 | 0.108 | 0.116 | 0.115 | 0.154 | 0.114 | 0.157 | 0.113 | 0.112 | 0.123 | 0.240 | 0.195 | 0.252 | **0.654** |
| *Note*. The diagonal line is the variances, marked in **bold** | | | | | | | | | | | | | | | | | | | | | | | | | | | |

| **Table F.2** Item covariance matrix of the KIDSCREEN-27 in male sample of the retest survey (*n* = 381) | | | | | | | | | | | | | | | | | | | | | | | | | | | |
| --- | --- | --- | --- | --- | --- | --- | --- | --- | --- | --- | --- | --- | --- | --- | --- | --- | --- | --- | --- | --- | --- | --- | --- | --- | --- | --- | --- |
|  | **Item1** | **Item2** | **Item3** | **Item4** | **Item5** | **Item6** | **Item7** | **Item8** | **Item9** | **Item10** | **Item11** | **Item12** | **Item13** | **Item14** | **Item15** | **Item16** | **Item17** | **Item18** | **Item19** | **Item20** | **Item21** | **Item22** | **Item23** | **Item24** | **Item25** | **Item26** | **Item27** |
| Item1 | **0.383** |  |  |  |  |  |  |  |  |  |  |  |  |  |  |  |  |  |  |  |  |  |  |  |  |  |  |
| Item2 | 0.111 | **0.339** |  |  |  |  |  |  |  |  |  |  |  |  |  |  |  |  |  |  |  |  |  |  |  |  |  |
| Item3 | 0.153 | 0.123 | **0.353** |  |  |  |  |  |  |  |  |  |  |  |  |  |  |  |  |  |  |  |  |  |  |  |  |
| Item4 | 0.113 | 0.119 | 0.090 | **0.338** |  |  |  |  |  |  |  |  |  |  |  |  |  |  |  |  |  |  |  |  |  |  |  |
| Item5 | 0.132 | 0.086 | 0.114 | 0.070 | **0.352** |  |  |  |  |  |  |  |  |  |  |  |  |  |  |  |  |  |  |  |  |  |  |
| Item6 | 0.050 | 0.054 | 0.029 | 0.038 | 0.060 | **0.410** |  |  |  |  |  |  |  |  |  |  |  |  |  |  |  |  |  |  |  |  |  |
| Item7 | 0.049 | 0.059 | 0.020 | 0.084 | 0.066 | 0.234 | **0.659** |  |  |  |  |  |  |  |  |  |  |  |  |  |  |  |  |  |  |  |  |
| Item8 | 0.117 | 0.046 | 0.071 | 0.026 | 0.086 | 0.188 | 0.230 | **0.601** |  |  |  |  |  |  |  |  |  |  |  |  |  |  |  |  |  |  |  |
| Item9 | 0.084 | 0.040 | 0.043 | 0.075 | 0.075 | 0.171 | 0.254 | 0.178 | **0.787** |  |  |  |  |  |  |  |  |  |  |  |  |  |  |  |  |  |  |
| Item10 | 0.094 | 0.102 | 0.058 | 0.107 | 0.073 | 0.141 | 0.264 | 0.185 | 0.373 | **0.842** |  |  |  |  |  |  |  |  |  |  |  |  |  |  |  |  |  |
| Item11 | 0.097 | 0.063 | 0.064 | 0.039 | 0.068 | 0.164 | 0.208 | 0.181 | 0.279 | 0.283 | **0.623** |  |  |  |  |  |  |  |  |  |  |  |  |  |  |  |  |
| Item12 | 0.122 | 0.028 | 0.069 | 0.076 | 0.095 | 0.163 | 0.153 | 0.165 | 0.242 | 0.149 | 0.200 | **0.821** |  |  |  |  |  |  |  |  |  |  |  |  |  |  |  |
| Item13 | 0.086 | 0.075 | 0.035 | 0.063 | 0.072 | 0.173 | 0.133 | 0.194 | 0.136 | 0.187 | 0.238 | 0.240 | **0.934** |  |  |  |  |  |  |  |  |  |  |  |  |  |  |
| Item14 | 0.041 | 0.060 | 0.022 | 0.043 | 0.048 | 0.167 | 0.157 | 0.179 | 0.242 | 0.249 | 0.267 | 0.243 | 0.547 | **1.040** |  |  |  |  |  |  |  |  |  |  |  |  |  |
| Item15 | 0.052 | 0.040 | 0.025 | 0.031 | 0.055 | 0.136 | 0.113 | 0.111 | 0.060 | 0.066 | 0.083 | 0.097 | 0.142 | 0.167 | **0.589** |  |  |  |  |  |  |  |  |  |  |  |  |
| Item16 | 0.056 | 0.014 | 0.002 | 0.051 | 0.072 | 0.088 | 0.123 | 0.093 | 0.092 | 0.081 | 0.088 | 0.064 | 0.155 | 0.148 | 0.202 | **0.484** |  |  |  |  |  |  |  |  |  |  |  |
| Item17 | 0.090 | 0.040 | 0.091 | 0.077 | 0.057 | 0.127 | 0.140 | 0.097 | 0.056 | 0.104 | 0.058 | 0.117 | 0.160 | 0.108 | 0.196 | 0.196 | **0.668** |  |  |  |  |  |  |  |  |  |  |
| Item18 | 0.052 | 0.106 | 0.084 | 0.060 | 0.086 | 0.145 | 0.188 | 0.134 | 0.233 | 0.257 | 0.207 | 0.138 | 0.247 | 0.225 | 0.110 | 0.093 | 0.132 | **1.064** |  |  |  |  |  |  |  |  |  |
| Item19 | 0.064 | 0.072 | 0.070 | 0.023 | 0.084 | 0.100 | 0.126 | 0.112 | 0.144 | 0.156 | 0.165 | 0.123 | 0.176 | 0.170 | 0.085 | 0.086 | 0.086 | 0.475 | **0.640** |  |  |  |  |  |  |  |  |
| Item20 | 0.071 | 0.052 | 0.022 | 0.050 | 0.042 | 0.060 | 0.092 | 0.073 | 0.160 | 0.100 | 0.116 | 0.081 | 0.118 | 0.114 | 0.057 | 0.100 | 0.061 | 0.178 | 0.122 | **0.623** |  |  |  |  |  |  |  |
| Item21 | 0.052 | 0.055 | 0.036 | 0.040 | 0.048 | 0.090 | 0.094 | 0.100 | 0.144 | 0.135 | 0.144 | 0.070 | 0.082 | 0.139 | 0.083 | 0.089 | 0.035 | 0.124 | 0.101 | 0.246 | **0.493** |  |  |  |  |  |  |
| Item22 | 0.086 | 0.098 | 0.039 | 0.055 | 0.060 | 0.100 | 0.109 | 0.121 | 0.144 | 0.136 | 0.138 | 0.065 | 0.094 | 0.091 | 0.075 | 0.101 | 0.040 | 0.158 | 0.130 | 0.228 | 0.203 | **0.453** |  |  |  |  |  |
| Item23 | 0.067 | 0.052 | 0.050 | 0.083 | 0.037 | 0.089 | 0.132 | 0.078 | 0.117 | 0.105 | 0.129 | 0.093 | 0.127 | 0.117 | 0.058 | 0.053 | 0.036 | 0.105 | 0.068 | 0.213 | 0.203 | 0.229 | **0.500** |  |  |  |  |
| Item24 | 0.084 | 0.061 | 0.058 | 0.045 | 0.060 | 0.131 | 0.180 | 0.189 | 0.232 | 0.184 | 0.173 | 0.188 | 0.244 | 0.260 | 0.114 | 0.100 | 0.117 | 0.179 | 0.128 | 0.154 | 0.131 | 0.121 | 0.131 | **0.601** |  |  |  |
| Item25 | 0.102 | 0.081 | 0.073 | 0.044 | 0.066 | 0.122 | 0.146 | 0.141 | 0.226 | 0.168 | 0.182 | 0.191 | 0.249 | 0.194 | 0.119 | 0.130 | 0.112 | 0.243 | 0.160 | 0.227 | 0.131 | 0.169 | 0.131 | 0.369 | **0.736** |  |  |
| Item26 | 0.097 | 0.082 | 0.095 | 0.021 | 0.107 | 0.142 | 0.127 | 0.158 | 0.198 | 0.189 | 0.176 | 0.212 | 0.191 | 0.174 | 0.138 | 0.116 | 0.135 | 0.218 | 0.178 | 0.167 | 0.181 | 0.155 | 0.157 | 0.282 | 0.316 | **0.859** |  |
| Item27 | 0.060 | 0.073 | 0.063 | 0.072 | 0.071 | 0.128 | 0.139 | 0.160 | 0.163 | 0.141 | 0.140 | 0.184 | 0.184 | 0.172 | 0.122 | 0.120 | 0.153 | 0.187 | 0.139 | 0.157 | 0.103 | 0.124 | 0.100 | 0.268 | 0.275 | 0.263 | **0.673** |
| *Note*. The diagonal line is the variances, marked in **bold** | | | | | | | | | | | | | | | | | | | | | | | | | | | |

| **Table F.3** Item covariance matrix of the KIDSCREEN-27 in female sample of the retest survey (*n* = 408) | | | | | | | | | | | | | | | | | | | | | | | | | | | |
| --- | --- | --- | --- | --- | --- | --- | --- | --- | --- | --- | --- | --- | --- | --- | --- | --- | --- | --- | --- | --- | --- | --- | --- | --- | --- | --- | --- |
|  | **Item1** | **Item2** | **Item3** | **Item4** | **Item5** | **Item6** | **Item7** | **Item8** | **Item9** | **Item10** | **Item11** | **Item12** | **Item13** | **Item14** | **Item15** | **Item16** | **Item17** | **Item18** | **Item19** | **Item20** | **Item21** | **Item22** | **Item23** | **Item24** | **Item25** | **Item26** | **Item27** |
| Item1 | **0.399** |  |  |  |  |  |  |  |  |  |  |  |  |  |  |  |  |  |  |  |  |  |  |  |  |  |  |
| Item2 | 0.129 | **0.375** |  |  |  |  |  |  |  |  |  |  |  |  |  |  |  |  |  |  |  |  |  |  |  |  |  |
| Item3 | 0.125 | 0.119 | **0.378** |  |  |  |  |  |  |  |  |  |  |  |  |  |  |  |  |  |  |  |  |  |  |  |  |
| Item4 | 0.116 | 0.120 | 0.112 | **0.320** |  |  |  |  |  |  |  |  |  |  |  |  |  |  |  |  |  |  |  |  |  |  |  |
| Item5 | 0.111 | 0.095 | 0.091 | 0.093 | **0.373** |  |  |  |  |  |  |  |  |  |  |  |  |  |  |  |  |  |  |  |  |  |  |
| Item6 | 0.075 | 0.037 | 0.024 | 0.022 | 0.044 | **0.405** |  |  |  |  |  |  |  |  |  |  |  |  |  |  |  |  |  |  |  |  |  |
| Item7 | 0.108 | 0.071 | 0.065 | 0.039 | 0.100 | 0.157 | **0.614** |  |  |  |  |  |  |  |  |  |  |  |  |  |  |  |  |  |  |  |  |
| Item8 | 0.076 | 0.071 | 0.056 | 0.059 | 0.079 | 0.108 | 0.155 | **0.435** |  |  |  |  |  |  |  |  |  |  |  |  |  |  |  |  |  |  |  |
| Item9 | 0.146 | 0.124 | 0.077 | 0.083 | 0.110 | 0.137 | 0.140 | 0.122 | **0.876** |  |  |  |  |  |  |  |  |  |  |  |  |  |  |  |  |  |  |
| Item10 | 0.171 | 0.124 | 0.123 | 0.068 | 0.120 | 0.153 | 0.217 | 0.154 | 0.505 | **0.905** |  |  |  |  |  |  |  |  |  |  |  |  |  |  |  |  |  |
| Item11 | 0.094 | 0.070 | 0.094 | 0.080 | 0.107 | 0.135 | 0.108 | 0.095 | 0.301 | 0.314 | **0.720** |  |  |  |  |  |  |  |  |  |  |  |  |  |  |  |  |
| Item12 | 0.104 | 0.088 | 0.080 | 0.088 | 0.103 | 0.109 | 0.113 | 0.105 | 0.216 | 0.234 | 0.211 | **0.971** |  |  |  |  |  |  |  |  |  |  |  |  |  |  |  |
| Item13 | 0.064 | 0.065 | 0.011 | −0.013 | 0.045 | 0.099 | 0.058 | 0.089 | 0.067 | 0.105 | 0.061 | 0.172 | **0.816** |  |  |  |  |  |  |  |  |  |  |  |  |  |  |
| Item14 | 0.068 | 0.114 | 0.046 | 0.027 | 0.029 | 0.130 | 0.062 | 0.094 | 0.097 | 0.097 | 0.123 | 0.161 | 0.507 | **1.072** |  |  |  |  |  |  |  |  |  |  |  |  |  |
| Item15 | 0.088 | 0.060 | 0.034 | 0.049 | 0.069 | 0.108 | 0.099 | 0.061 | 0.145 | 0.117 | 0.130 | 0.114 | 0.113 | 0.142 | **0.506** |  |  |  |  |  |  |  |  |  |  |  |  |
| Item16 | 0.070 | 0.045 | 0.026 | 0.052 | 0.043 | 0.103 | 0.071 | 0.052 | 0.136 | 0.138 | 0.144 | 0.119 | 0.043 | 0.053 | 0.156 | **0.493** |  |  |  |  |  |  |  |  |  |  |  |
| Item17 | 0.093 | 0.056 | 0.053 | 0.084 | 0.068 | 0.116 | 0.083 | 0.066 | 0.109 | 0.134 | 0.127 | 0.117 | 0.073 | 0.124 | 0.168 | 0.182 | **0.599** |  |  |  |  |  |  |  |  |  |  |
| Item18 | 0.091 | 0.156 | 0.100 | 0.096 | 0.051 | 0.096 | 0.095 | 0.108 | 0.172 | 0.138 | 0.116 | 0.109 | 0.065 | 0.138 | 0.103 | 0.121 | 0.205 | **1.006** |  |  |  |  |  |  |  |  |  |
| Item19 | 0.063 | 0.119 | 0.045 | 0.081 | 0.034 | 0.068 | 0.040 | 0.045 | 0.112 | 0.114 | 0.125 | 0.068 | 0.079 | 0.110 | 0.061 | 0.093 | 0.132 | 0.424 | **0.619** |  |  |  |  |  |  |  |  |
| Item20 | 0.092 | 0.074 | 0.050 | 0.042 | 0.082 | 0.073 | 0.099 | 0.060 | 0.127 | 0.156 | 0.106 | 0.117 | 0.140 | 0.131 | 0.063 | 0.057 | 0.088 | 0.161 | 0.118 | **0.499** |  |  |  |  |  |  |  |
| Item21 | 0.079 | 0.052 | 0.055 | 0.044 | 0.028 | 0.078 | 0.107 | 0.072 | 0.136 | 0.142 | 0.119 | 0.112 | 0.087 | 0.075 | 0.064 | 0.063 | 0.078 | 0.096 | 0.040 | 0.135 | **0.432** |  |  |  |  |  |  |
| Item22 | 0.070 | 0.044 | 0.037 | 0.044 | 0.055 | 0.057 | 0.050 | 0.062 | 0.118 | 0.120 | 0.071 | 0.124 | 0.051 | 0.070 | 0.072 | 0.096 | 0.071 | 0.093 | 0.042 | 0.151 | 0.156 | **0.385** |  |  |  |  |  |
| Item23 | 0.086 | 0.061 | 0.046 | 0.046 | 0.061 | 0.071 | 0.105 | 0.108 | 0.120 | 0.158 | 0.131 | 0.131 | 0.068 | 0.128 | 0.061 | 0.067 | 0.104 | 0.126 | 0.076 | 0.137 | 0.153 | 0.169 | **0.396** |  |  |  |  |
| Item24 | 0.113 | 0.091 | 0.058 | 0.068 | 0.108 | 0.128 | 0.123 | 0.120 | 0.215 | 0.276 | 0.222 | 0.173 | 0.174 | 0.227 | 0.124 | 0.108 | 0.122 | 0.200 | 0.146 | 0.170 | 0.103 | 0.096 | 0.109 | **0.606** |  |  |  |
| Item25 | 0.107 | 0.098 | 0.074 | 0.090 | 0.108 | 0.090 | 0.094 | 0.109 | 0.228 | 0.205 | 0.178 | 0.200 | 0.132 | 0.129 | 0.129 | 0.089 | 0.131 | 0.236 | 0.152 | 0.098 | 0.094 | 0.084 | 0.101 | 0.307 | **0.552** |  |  |
| Item26 | 0.116 | 0.113 | 0.124 | 0.093 | 0.108 | 0.096 | 0.089 | 0.095 | 0.252 | 0.261 | 0.261 | 0.236 | 0.175 | 0.194 | 0.126 | 0.109 | 0.116 | 0.254 | 0.153 | 0.142 | 0.130 | 0.121 | 0.161 | 0.286 | 0.256 | **0.784** |  |
| Item27 | 0.124 | 0.096 | 0.055 | 0.046 | 0.042 | 0.130 | 0.109 | 0.103 | 0.173 | 0.218 | 0.161 | 0.134 | 0.133 | 0.179 | 0.097 | 0.115 | 0.080 | 0.123 | 0.090 | 0.156 | 0.118 | 0.099 | 0.142 | 0.212 | 0.119 | 0.241 | **0.633** |
| *Note*. The diagonal line is the variances, marked in **bold** | | | | | | | | | | | | | | | | | | | | | | | | | | | |

| **Table F.4** Item covariance matrix of the KIDSCREEN-27 in primary school sample of the retest survey (*n* = 162) | | | | | | | | | | | | | | | | | | | | | | | | | | | |
| --- | --- | --- | --- | --- | --- | --- | --- | --- | --- | --- | --- | --- | --- | --- | --- | --- | --- | --- | --- | --- | --- | --- | --- | --- | --- | --- | --- |
|  | **Item1** | **Item2** | **Item3** | **Item4** | **Item5** | **Item6** | **Item7** | **Item8** | **Item9** | **Item10** | **Item11** | **Item12** | **Item13** | **Item14** | **Item15** | **Item16** | **Item17** | **Item18** | **Item19** | **Item20** | **Item21** | **Item22** | **Item23** | **Item24** | **Item25** | **Item26** | **Item27** |
| Item1 | **0.238** |  |  |  |  |  |  |  |  |  |  |  |  |  |  |  |  |  |  |  |  |  |  |  |  |  |  |
| Item2 | 0.036 | **0.238** |  |  |  |  |  |  |  |  |  |  |  |  |  |  |  |  |  |  |  |  |  |  |  |  |  |
| Item3 | 0.063 | 0.061 | **0.371** |  |  |  |  |  |  |  |  |  |  |  |  |  |  |  |  |  |  |  |  |  |  |  |  |
| Item4 | 0.057 | 0.040 | 0.041 | **0.258** |  |  |  |  |  |  |  |  |  |  |  |  |  |  |  |  |  |  |  |  |  |  |  |
| Item5 | 0.007 | 0.029 | 0.039 | 0.050 | **0.304** |  |  |  |  |  |  |  |  |  |  |  |  |  |  |  |  |  |  |  |  |  |  |
| Item6 | 0.026 | 0.046 | 0.035 | 0.024 | 0.008 | **0.275** |  |  |  |  |  |  |  |  |  |  |  |  |  |  |  |  |  |  |  |  |  |
| Item7 | −0.010 | 0.049 | 0.036 | 0.018 | 0.059 | 0.078 | **0.476** |  |  |  |  |  |  |  |  |  |  |  |  |  |  |  |  |  |  |  |  |
| Item8 | 0.045 | 0.021 | 0.025 | 0.053 | 0.007 | 0.080 | 0.144 | **0.417** |  |  |  |  |  |  |  |  |  |  |  |  |  |  |  |  |  |  |  |
| Item9 | −0.032 | 0.036 | 0.037 | 0.058 | 0.017 | 0.104 | 0.038 | 0.029 | **0.504** |  |  |  |  |  |  |  |  |  |  |  |  |  |  |  |  |  |  |
| Item10 | 0.012 | 0.120 | 0.045 | 0.066 | 0.012 | 0.101 | 0.144 | 0.075 | 0.157 | **0.577** |  |  |  |  |  |  |  |  |  |  |  |  |  |  |  |  |  |
| Item11 | 0.027 | 0.037 | 0.008 | 0.049 | 0.039 | 0.090 | 0.095 | 0.097 | 0.129 | 0.166 | **0.507** |  |  |  |  |  |  |  |  |  |  |  |  |  |  |  |  |
| Item12 | 0.044 | −0.030 | 0.047 | 0.058 | 0.056 | 0.056 | 0.085 | 0.103 | 0.055 | 0.059 | 0.176 | **0.527** |  |  |  |  |  |  |  |  |  |  |  |  |  |  |  |
| Item13 | 0.055 | 0.047 | 0.005 | −0.010 | 0.020 | 0.056 | 0.040 | 0.131 | 0.049 | 0.098 | 0.092 | 0.131 | **0.616** |  |  |  |  |  |  |  |  |  |  |  |  |  |  |
| Item14 | −0.009 | 0.062 | 0.011 | 0.026 | −0.077 | 0.096 | 0.122 | 0.114 | 0.082 | 0.169 | 0.096 | 0.054 | 0.175 | **0.743** |  |  |  |  |  |  |  |  |  |  |  |  |  |
| Item15 | 0.001 | 0.042 | −0.020 | −0.015 | 0.039 | 0.109 | 0.036 | 0.075 | 0.093 | 0.032 | 0.083 | 0.016 | 0.148 | 0.091 | **0.446** |  |  |  |  |  |  |  |  |  |  |  |  |
| Item16 | 0.018 | −0.002 | 0.009 | 0.056 | 0.022 | 0.046 | 0.061 | 0.012 | 0.094 | 0.075 | 0.098 | 0.077 | 0.080 | 0.038 | 0.033 | **0.340** |  |  |  |  |  |  |  |  |  |  |  |
| Item17 | 0.052 | 0.039 | 0.055 | 0.078 | 0.052 | 0.092 | 0.093 | 0.070 | 0.108 | 0.094 | 0.119 | 0.088 | 0.064 | 0.080 | 0.055 | 0.079 | **0.534** |  |  |  |  |  |  |  |  |  |  |
| Item18 | −0.006 | 0.070 | 0.056 | 0.056 | 0.115 | 0.184 | 0.091 | 0.054 | 0.181 | 0.146 | 0.167 | 0.024 | 0.067 | 0.081 | 0.162 | 0.088 | 0.149 | **0.972** |  |  |  |  |  |  |  |  |  |
| Item19 | −0.015 | 0.079 | 0.016 | 0.024 | 0.048 | 0.102 | 0.052 | 0.071 | 0.122 | 0.069 | 0.117 | 0.020 | 0.116 | 0.063 | 0.060 | 0.097 | 0.140 | 0.335 | **0.538** |  |  |  |  |  |  |  |  |
| Item20 | 0.057 | 0.032 | 0.053 | 0.060 | 0.058 | 0.089 | 0.101 | 0.093 | 0.107 | 0.101 | 0.112 | 0.115 | 0.079 | 0.075 | 0.040 | 0.042 | 0.121 | 0.177 | 0.117 | **0.411** |  |  |  |  |  |  |  |
| Item21 | 0.002 | 0.007 | 0.027 | 0.024 | 0.031 | 0.103 | 0.066 | 0.034 | 0.069 | 0.105 | 0.069 | 0.029 | 0.053 | 0.142 | 0.027 | 0.050 | 0.034 | 0.065 | 0.035 | 0.071 | **0.305** |  |  |  |  |  |  |
| Item22 | 0.033 | 0.050 | 0.071 | 0.031 | 0.050 | 0.097 | 0.066 | 0.065 | 0.063 | 0.117 | 0.088 | 0.091 | 0.091 | 0.086 | 0.083 | 0.062 | 0.053 | 0.128 | 0.085 | 0.114 | 0.063 | **0.292** |  |  |  |  |  |
| Item23 | 0.030 | 0.042 | 0.040 | 0.046 | 0.035 | 0.091 | 0.104 | 0.069 | 0.030 | 0.104 | 0.093 | 0.084 | 0.114 | 0.118 | 0.046 | 0.026 | 0.078 | 0.121 | 0.063 | 0.074 | 0.066 | 0.091 | **0.286** |  |  |  |  |
| Item24 | 0.010 | 0.038 | 0.014 | 0.001 | 0.022 | 0.046 | 0.083 | 0.055 | 0.074 | 0.048 | 0.060 | 0.034 | 0.084 | 0.058 | 0.082 | 0.033 | 0.119 | 0.065 | 0.072 | 0.036 | 0.021 | 0.039 | 0.058 | **0.365** |  |  |  |
| Item25 | 0.025 | 0.043 | 0.040 | 0.002 | 0.065 | 0.064 | 0.093 | 0.118 | 0.097 | 0.050 | 0.058 | 0.056 | 0.114 | 0.029 | 0.115 | 0.033 | 0.107 | 0.176 | 0.088 | 0.105 | 0.007 | 0.045 | 0.081 | 0.124 | **0.507** |  |  |
| Item26 | −0.004 | 0.002 | 0.022 | 0.010 | 0.002 | 0.037 | 0.016 | 0.005 | 0.074 | 0.097 | 0.058 | 0.012 | 0.091 | 0.079 | 0.059 | 0.043 | 0.020 | 0.095 | 0.093 | 0.045 | 0.049 | 0.074 | 0.073 | 0.028 | 0.047 | **0.298** |  |
| Item27 | 0.047 | 0.042 | 0.041 | 0.022 | 0.009 | 0.082 | 0.085 | 0.044 | 0.071 | 0.053 | 0.076 | 0.084 | 0.044 | 0.030 | 0.041 | 0.020 | 0.001 | 0.083 | 0.086 | 0.113 | 0.019 | 0.087 | 0.042 | 0.051 | 0.057 | 0.041 | **0.393** |
| *Note*. The diagonal line is the variances, marked in **bold** | | | | | | | | | | | | | | | | | | | | | | | | | | | |

| **Table F.5** Item covariance matrix of the KIDSCREEN-27 in junior secondary school sample of the retest survey (*n* = 355) | | | | | | | | | | | | | | | | | | | | | | | | | | | |
| --- | --- | --- | --- | --- | --- | --- | --- | --- | --- | --- | --- | --- | --- | --- | --- | --- | --- | --- | --- | --- | --- | --- | --- | --- | --- | --- | --- |
|  | **Item1** | **Item2** | **Item3** | **Item4** | **Item5** | **Item6** | **Item7** | **Item8** | **Item9** | **Item10** | **Item11** | **Item12** | **Item13** | **Item14** | **Item15** | **Item16** | **Item17** | **Item18** | **Item19** | **Item20** | **Item21** | **Item22** | **Item23** | **Item24** | **Item25** | **Item26** | **Item27** |
| Item1 | **0.505** |  |  |  |  |  |  |  |  |  |  |  |  |  |  |  |  |  |  |  |  |  |  |  |  |  |  |
| Item2 | 0.170 | **0.450** |  |  |  |  |  |  |  |  |  |  |  |  |  |  |  |  |  |  |  |  |  |  |  |  |  |
| Item3 | 0.176 | 0.158 | **0.361** |  |  |  |  |  |  |  |  |  |  |  |  |  |  |  |  |  |  |  |  |  |  |  |  |
| Item4 | 0.155 | 0.178 | 0.130 | **0.399** |  |  |  |  |  |  |  |  |  |  |  |  |  |  |  |  |  |  |  |  |  |  |  |
| Item5 | 0.197 | 0.125 | 0.125 | 0.100 | **0.426** |  |  |  |  |  |  |  |  |  |  |  |  |  |  |  |  |  |  |  |  |  |  |
| Item6 | 0.045 | 0.028 | −0.004 | 0.027 | 0.049 | **0.415** |  |  |  |  |  |  |  |  |  |  |  |  |  |  |  |  |  |  |  |  |  |
| Item7 | 0.138 | 0.081 | 0.044 | 0.086 | 0.107 | 0.205 | **0.685** |  |  |  |  |  |  |  |  |  |  |  |  |  |  |  |  |  |  |  |  |
| Item8 | 0.109 | 0.047 | 0.050 | 0.036 | 0.095 | 0.168 | 0.193 | **0.488** |  |  |  |  |  |  |  |  |  |  |  |  |  |  |  |  |  |  |  |
| Item9 | 0.144 | 0.089 | 0.031 | 0.085 | 0.120 | 0.147 | 0.203 | 0.146 | **0.889** |  |  |  |  |  |  |  |  |  |  |  |  |  |  |  |  |  |  |
| Item10 | 0.159 | 0.133 | 0.079 | 0.118 | 0.107 | 0.122 | 0.261 | 0.161 | 0.509 | **0.969** |  |  |  |  |  |  |  |  |  |  |  |  |  |  |  |  |  |
| Item11 | 0.109 | 0.059 | 0.056 | 0.039 | 0.086 | 0.146 | 0.144 | 0.126 | 0.267 | 0.302 | **0.658** |  |  |  |  |  |  |  |  |  |  |  |  |  |  |  |  |
| Item12 | 0.098 | 0.070 | 0.018 | 0.099 | 0.108 | 0.160 | 0.172 | 0.088 | 0.250 | 0.190 | 0.193 | **0.966** |  |  |  |  |  |  |  |  |  |  |  |  |  |  |  |
| Item13 | 0.081 | 0.066 | −0.009 | 0.041 | 0.011 | 0.158 | 0.144 | 0.142 | 0.103 | 0.147 | 0.159 | 0.198 | **0.870** |  |  |  |  |  |  |  |  |  |  |  |  |  |  |
| Item14 | 0.051 | 0.066 | −0.009 | 0.043 | 0.049 | 0.149 | 0.097 | 0.116 | 0.131 | 0.137 | 0.205 | 0.179 | 0.547 | **1.087** |  |  |  |  |  |  |  |  |  |  |  |  |  |
| Item15 | 0.118 | 0.054 | 0.062 | 0.077 | 0.082 | 0.141 | 0.154 | 0.114 | 0.110 | 0.106 | 0.137 | 0.128 | 0.127 | 0.185 | **0.672** |  |  |  |  |  |  |  |  |  |  |  |  |
| Item16 | 0.077 | 0.017 | −0.005 | 0.050 | 0.064 | 0.134 | 0.138 | 0.119 | 0.154 | 0.154 | 0.169 | 0.102 | 0.136 | 0.143 | 0.250 | **0.578** |  |  |  |  |  |  |  |  |  |  |  |
| Item17 | 0.116 | 0.075 | 0.065 | 0.096 | 0.055 | 0.162 | 0.186 | 0.109 | 0.112 | 0.167 | 0.126 | 0.157 | 0.188 | 0.213 | 0.266 | 0.247 | **0.708** |  |  |  |  |  |  |  |  |  |  |
| Item18 | 0.108 | 0.141 | 0.081 | 0.096 | 0.057 | 0.084 | 0.175 | 0.139 | 0.205 | 0.265 | 0.141 | 0.104 | 0.142 | 0.120 | 0.123 | 0.105 | 0.208 | **1.116** |  |  |  |  |  |  |  |  |  |
| Item19 | 0.099 | 0.093 | 0.032 | 0.061 | 0.059 | 0.055 | 0.108 | 0.066 | 0.114 | 0.178 | 0.127 | 0.089 | 0.059 | 0.074 | 0.064 | 0.094 | 0.088 | 0.493 | **0.683** |  |  |  |  |  |  |  |  |
| Item20 | 0.111 | 0.062 | 0.032 | 0.047 | 0.052 | 0.081 | 0.083 | 0.072 | 0.151 | 0.127 | 0.140 | 0.114 | 0.161 | 0.124 | 0.068 | 0.084 | 0.067 | 0.202 | 0.133 | **0.668** |  |  |  |  |  |  |  |
| Item21 | 0.083 | 0.065 | 0.012 | 0.036 | 0.033 | 0.067 | 0.116 | 0.071 | 0.179 | 0.157 | 0.141 | 0.105 | 0.130 | 0.109 | 0.078 | 0.078 | 0.058 | 0.110 | 0.077 | 0.268 | **0.533** |  |  |  |  |  |  |
| Item22 | 0.098 | 0.062 | 0.000 | 0.038 | 0.047 | 0.082 | 0.059 | 0.075 | 0.164 | 0.158 | 0.110 | 0.114 | 0.107 | 0.099 | 0.069 | 0.105 | 0.045 | 0.156 | 0.111 | 0.262 | 0.237 | **0.509** |  |  |  |  |  |
| Item23 | 0.108 | 0.065 | 0.037 | 0.075 | 0.044 | 0.109 | 0.118 | 0.077 | 0.134 | 0.123 | 0.130 | 0.122 | 0.102 | 0.117 | 0.101 | 0.103 | 0.112 | 0.141 | 0.068 | 0.229 | 0.211 | 0.252 | **0.538** |  |  |  |  |
| Item24 | 0.138 | 0.065 | 0.047 | 0.072 | 0.076 | 0.146 | 0.198 | 0.136 | 0.231 | 0.269 | 0.216 | 0.167 | 0.216 | 0.189 | 0.134 | 0.166 | 0.186 | 0.239 | 0.169 | 0.222 | 0.163 | 0.165 | 0.138 | **0.639** |  |  |  |
| Item25 | 0.168 | 0.109 | 0.078 | 0.101 | 0.091 | 0.129 | 0.185 | 0.133 | 0.309 | 0.274 | 0.256 | 0.215 | 0.177 | 0.137 | 0.149 | 0.179 | 0.164 | 0.266 | 0.199 | 0.222 | 0.171 | 0.206 | 0.168 | 0.446 | **0.833** |  |  |
| Item26 | 0.135 | 0.075 | 0.088 | 0.031 | 0.097 | 0.111 | 0.161 | 0.106 | 0.288 | 0.241 | 0.242 | 0.231 | 0.127 | 0.110 | 0.151 | 0.129 | 0.172 | 0.273 | 0.183 | 0.181 | 0.216 | 0.178 | 0.208 | 0.328 | 0.381 | **0.916** |  |
| Item27 | 0.110 | 0.089 | 0.043 | 0.076 | 0.047 | 0.127 | 0.177 | 0.119 | 0.153 | 0.208 | 0.148 | 0.139 | 0.244 | 0.192 | 0.133 | 0.164 | 0.210 | 0.217 | 0.138 | 0.190 | 0.136 | 0.122 | 0.170 | 0.313 | 0.256 | 0.321 | **0.702** |
| *Note*. The diagonal line is the variances, marked in **bold** | | | | | | | | | | | | | | | | | | | | | | | | | | | |

| **Table F.6** Item covariance matrix of the KIDSCREEN-27 in senior secondary school sample of the retest survey (*n* = 272) | | | | | | | | | | | | | | | | | | | | | | | | | | | |
| --- | --- | --- | --- | --- | --- | --- | --- | --- | --- | --- | --- | --- | --- | --- | --- | --- | --- | --- | --- | --- | --- | --- | --- | --- | --- | --- | --- |
|  | **Item1** | **Item2** | **Item3** | **Item4** | **Item5** | **Item6** | **Item7** | **Item8** | **Item9** | **Item10** | **Item11** | **Item12** | **Item13** | **Item14** | **Item15** | **Item16** | **Item17** | **Item18** | **Item19** | **Item20** | **Item21** | **Item22** | **Item23** | **Item24** | **Item25** | **Item26** | **Item27** |
| Item1 | **0.326** |  |  |  |  |  |  |  |  |  |  |  |  |  |  |  |  |  |  |  |  |  |  |  |  |  |  |
| Item2 | 0.087 | **0.284** |  |  |  |  |  |  |  |  |  |  |  |  |  |  |  |  |  |  |  |  |  |  |  |  |  |
| Item3 | 0.127 | 0.096 | **0.363** |  |  |  |  |  |  |  |  |  |  |  |  |  |  |  |  |  |  |  |  |  |  |  |  |
| Item4 | 0.086 | 0.078 | 0.094 | **0.274** |  |  |  |  |  |  |  |  |  |  |  |  |  |  |  |  |  |  |  |  |  |  |  |
| Item5 | 0.078 | 0.061 | 0.097 | 0.068 | **0.299** |  |  |  |  |  |  |  |  |  |  |  |  |  |  |  |  |  |  |  |  |  |  |
| Item6 | 0.098 | 0.043 | 0.046 | 0.023 | 0.064 | **0.465** |  |  |  |  |  |  |  |  |  |  |  |  |  |  |  |  |  |  |  |  |  |
| Item7 | 0.049 | 0.035 | 0.038 | 0.042 | 0.058 | 0.246 | **0.668** |  |  |  |  |  |  |  |  |  |  |  |  |  |  |  |  |  |  |  |  |
| Item8 | 0.099 | 0.076 | 0.089 | 0.037 | 0.091 | 0.137 | 0.212 | **0.575** |  |  |  |  |  |  |  |  |  |  |  |  |  |  |  |  |  |  |  |
| Item9 | 0.134 | 0.032 | 0.076 | 0.046 | 0.051 | 0.150 | 0.259 | 0.169 | **0.820** |  |  |  |  |  |  |  |  |  |  |  |  |  |  |  |  |  |  |
| Item10 | 0.153 | 0.044 | 0.117 | 0.037 | 0.108 | 0.185 | 0.258 | 0.212 | 0.450 | **0.891** |  |  |  |  |  |  |  |  |  |  |  |  |  |  |  |  |  |
| Item11 | 0.108 | 0.067 | 0.136 | 0.082 | 0.096 | 0.165 | 0.202 | 0.133 | 0.354 | 0.347 | **0.746** |  |  |  |  |  |  |  |  |  |  |  |  |  |  |  |  |
| Item12 | 0.144 | 0.045 | 0.137 | 0.051 | 0.068 | 0.104 | 0.085 | 0.155 | 0.175 | 0.209 | 0.173 | **0.922** |  |  |  |  |  |  |  |  |  |  |  |  |  |  |  |
| Item13 | 0.053 | 0.031 | 0.039 | −0.007 | 0.096 | 0.107 | 0.047 | 0.074 | 0.001 | 0.107 | 0.082 | 0.133 | **0.892** |  |  |  |  |  |  |  |  |  |  |  |  |  |  |
| Item14 | 0.065 | 0.059 | 0.060 | −0.006 | 0.031 | 0.114 | 0.093 | 0.081 | 0.092 | 0.132 | 0.125 | 0.149 | 0.521 | **0.950** |  |  |  |  |  |  |  |  |  |  |  |  |  |
| Item15 | 0.035 | 0.026 | 0.006 | 0.013 | 0.035 | 0.088 | 0.071 | 0.045 | 0.051 | 0.081 | 0.074 | 0.095 | 0.083 | 0.112 | **0.420** |  |  |  |  |  |  |  |  |  |  |  |  |
| Item16 | 0.053 | 0.034 | 0.034 | 0.033 | 0.047 | 0.062 | 0.045 | 0.047 | 0.018 | 0.039 | 0.059 | 0.053 | 0.037 | 0.052 | 0.136 | **0.404** |  |  |  |  |  |  |  |  |  |  |  |
| Item17 | 0.066 | −0.007 | 0.083 | 0.046 | 0.066 | 0.081 | 0.007 | 0.065 | −0.007 | 0.044 | 0.054 | 0.067 | 0.057 | 0.020 | 0.113 | 0.115 | **0.522** |  |  |  |  |  |  |  |  |  |  |
| Item18 | 0.040 | 0.089 | 0.095 | 0.031 | 0.008 | 0.094 | 0.105 | 0.098 | 0.087 | 0.068 | 0.136 | 0.098 | 0.120 | 0.183 | −0.001 | 0.054 | 0.076 | **0.850** |  |  |  |  |  |  |  |  |  |
| Item19 | 0.043 | 0.065 | 0.091 | 0.035 | 0.031 | 0.083 | 0.051 | 0.064 | 0.063 | 0.071 | 0.145 | 0.067 | 0.143 | 0.166 | 0.060 | 0.040 | 0.093 | 0.382 | **0.562** |  |  |  |  |  |  |  |  |
| Item20 | 0.039 | 0.048 | 0.020 | 0.016 | 0.058 | 0.022 | 0.093 | 0.044 | 0.096 | 0.112 | 0.072 | 0.033 | 0.096 | 0.122 | 0.025 | 0.028 | −0.006 | 0.055 | 0.064 | **0.445** |  |  |  |  |  |  |  |
| Item21 | 0.071 | 0.036 | 0.089 | 0.044 | 0.031 | 0.089 | 0.098 | 0.127 | 0.095 | 0.114 | 0.148 | 0.075 | 0.022 | 0.048 | 0.070 | 0.056 | 0.039 | 0.097 | 0.056 | 0.124 | **0.452** |  |  |  |  |  |  |
| Item22 | 0.070 | 0.070 | 0.060 | 0.063 | 0.065 | 0.059 | 0.108 | 0.126 | 0.103 | 0.080 | 0.105 | 0.051 | 0.006 | 0.038 | 0.056 | 0.082 | 0.041 | 0.054 | 0.033 | 0.108 | 0.161 | **0.365** |  |  |  |  |  |
| Item23 | 0.051 | 0.023 | 0.054 | 0.044 | 0.045 | 0.026 | 0.121 | 0.119 | 0.108 | 0.136 | 0.140 | 0.075 | 0.049 | 0.088 | −0.009 | −0.007 | −0.014 | 0.036 | 0.054 | 0.131 | 0.186 | 0.181 | **0.407** |  |  |  |  |
| Item24 | 0.077 | 0.046 | 0.057 | 0.035 | 0.082 | 0.116 | 0.121 | 0.160 | 0.176 | 0.232 | 0.174 | 0.146 | 0.124 | 0.221 | 0.089 | 0.045 | 0.041 | 0.102 | 0.058 | 0.140 | 0.097 | 0.070 | 0.106 | **0.554** |  |  |  |
| Item25 | 0.046 | 0.036 | 0.056 | 0.035 | 0.057 | 0.069 | 0.041 | 0.065 | 0.098 | 0.106 | 0.095 | 0.147 | 0.141 | 0.120 | 0.067 | 0.035 | 0.068 | 0.161 | 0.079 | 0.087 | 0.082 | 0.061 | 0.044 | 0.216 | **0.394** |  |  |
| Item26 | 0.105 | 0.110 | 0.150 | 0.084 | 0.131 | 0.134 | 0.079 | 0.155 | 0.100 | 0.217 | 0.212 | 0.203 | 0.171 | 0.152 | 0.107 | 0.090 | 0.110 | 0.162 | 0.101 | 0.143 | 0.112 | 0.108 | 0.111 | 0.243 | 0.198 | **0.870** |  |
| Item27 | 0.077 | 0.049 | 0.061 | 0.026 | 0.060 | 0.139 | 0.069 | 0.164 | 0.162 | 0.176 | 0.164 | 0.141 | 0.040 | 0.140 | 0.081 | 0.067 | 0.023 | 0.036 | 0.046 | 0.095 | 0.113 | 0.096 | 0.081 | 0.189 | 0.143 | 0.212 | **0.697** |
| *Note*. The diagonal line is the variances, marked in **bold** | | | | | | | | | | | | | | | | | | | | | | | | | | | |

| **Table G.1** Item covariance matrix of the KIDSCREEN-10 index in the baseline survey (*N* = 4291) | | | | | | | | | | |
| --- | --- | --- | --- | --- | --- | --- | --- | --- | --- | --- |
|  | **Item2** | **Item5** | **Item9** | **Item11** | **Item13** | **Item14** | **Item16** | **Item21** | **Item25** | **Item26** |
| Item2 | **0.363** |  |  |  |  |  |  |  |  |  |
| Item5 | 0.207 | **0.404** |  |  |  |  |  |  |  |  |
| Item9 | 0.167 | 0.169 | **0.862** |  |  |  |  |  |  |  |
| Item11 | 0.179 | 0.186 | 0.668 | **0.953** |  |  |  |  |  |  |
| Item13 | 0.150 | 0.140 | 0.325 | 0.345 | **1.027** |  |  |  |  |  |
| Item14 | 0.139 | 0.137 | 0.314 | 0.342 | 0.901 | **1.038** |  |  |  |  |
| Item16 | 0.085 | 0.101 | 0.224 | 0.267 | 0.235 | 0.237 | **0.570** |  |  |  |
| Item21 | 0.106 | 0.125 | 0.235 | 0.264 | 0.205 | 0.199 | 0.184 | **0.470** |  |  |
| Item25 | 0.159 | 0.160 | 0.330 | 0.335 | 0.306 | 0.303 | 0.194 | 0.230 | **0.695** |  |
| Item26 | 0.135 | 0.143 | 0.305 | 0.327 | 0.252 | 0.248 | 0.204 | 0.221 | 0.429 | **0.557** |
| *Note*. The diagonal line is the variances, marked in **bold** | | | | | | | | | | |

| **Table G.2** Item covariance matrices of the gender subgroup for the KIDSCREEN-10 index in the baseline survey | | | | | | | | | | |
| --- | --- | --- | --- | --- | --- | --- | --- | --- | --- | --- |
|  | **Item2** | **Item5** | **Item9** | **Item11** | **Item13** | **Item14** | **Item16** | **Item21** | **Item25** | **Item26** |
| Male sample (*n* = 2011) | | | | | | | | | | |
| Item2 | **0.364** |  |  |  |  |  |  |  |  |  |
| Item5 | 0.215 | **0.417** |  |  |  |  |  |  |  |  |
| Item9 | 0.154 | 0.172 | **0.863** |  |  |  |  |  |  |  |
| Item11 | 0.171 | 0.182 | 0.667 | **0.945** |  |  |  |  |  |  |
| Item13 | 0.135 | 0.142 | 0.329 | 0.351 | **1.056** |  |  |  |  |  |
| Item14 | 0.121 | 0.140 | 0.308 | 0.342 | 0.927 | **1.045** |  |  |  |  |
| Item16 | 0.074 | 0.092 | 0.227 | 0.276 | 0.251 | 0.249 | **0.575** |  |  |  |
| Item21 | 0.117 | 0.140 | 0.263 | 0.288 | 0.221 | 0.207 | 0.188 | **0.492** |  |  |
| Item25 | 0.166 | 0.175 | 0.349 | 0.361 | 0.313 | 0.307 | 0.215 | 0.265 | **0.734** |  |
| Item26 | 0.130 | 0.146 | 0.310 | 0.337 | 0.252 | 0.248 | 0.213 | 0.252 | 0.463 | **0.586** |
| Female sample (*n* = 2280) | | | | | | | | | | |
| Item2 | **0.356** |  |  |  |  |  |  |  |  |  |
| Item5 | 0.192 | **0.384** |  |  |  |  |  |  |  |  |
| Item9 | 0.173 | 0.162 | **0.858** |  |  |  |  |  |  |  |
| Item11 | 0.180 | 0.184 | 0.665 | **0.955** |  |  |  |  |  |  |
| Item13 | 0.150 | 0.125 | 0.314 | 0.329 | **0.981** |  |  |  |  |  |
| Item14 | 0.145 | 0.124 | 0.313 | 0.333 | 0.862 | **1.019** |  |  |  |  |
| Item16 | 0.095 | 0.109 | 0.222 | 0.259 | 0.222 | 0.228 | **0.566** |  |  |  |
| Item21 | 0.101 | 0.115 | 0.212 | 0.246 | 0.198 | 0.197 | 0.180 | **0.448** |  |  |
| Item25 | 0.154 | 0.147 | 0.314 | 0.312 | 0.300 | 0.300 | 0.176 | 0.199 | **0.661** |  |
| Item26 | 0.142 | 0.143 | 0.301 | 0.320 | 0.255 | 0.250 | 0.196 | 0.194 | 0.400 | **0.532** |
| *Note*. The diagonal line is the variances, marked in **bold** | | | | | | | | | | |

| **Table G.3** Item covariance matrices of the grade subgroup for the KIDSCREEN-10 index in the baseline survey | | | | | | | | | | |
| --- | --- | --- | --- | --- | --- | --- | --- | --- | --- | --- |
|  | **Item2** | **Item5** | **Item9** | **Item11** | **Item13** | **Item14** | **Item16** | **Item21** | **Item25** | **Item26** |
| Primary school sample (*n* = 947) | | | | | | | | | | |
| Item2 | **0.259** |  |  |  |  |  |  |  |  |  |
| Item5 | 0.087 | **0.261** |  |  |  |  |  |  |  |  |
| Item9 | 0.093 | 0.080 | **0.717** |  |  |  |  |  |  |  |
| Item11 | 0.081 | 0.080 | 0.506 | **0.728** |  |  |  |  |  |  |
| Item13 | 0.096 | 0.093 | 0.274 | 0.284 | **0.788** |  |  |  |  |  |
| Item14 | 0.097 | 0.102 | 0.274 | 0.305 | 0.621 | **0.737** |  |  |  |  |
| Item16 | 0.046 | 0.034 | 0.194 | 0.197 | 0.225 | 0.213 | **0.437** |  |  |  |
| Item21 | 0.076 | 0.077 | 0.242 | 0.217 | 0.236 | 0.229 | 0.152 | **0.400** |  |  |
| Item25 | 0.099 | 0.074 | 0.279 | 0.244 | 0.253 | 0.267 | 0.195 | 0.225 | **0.598** |  |
| Item26 | 0.070 | 0.058 | 0.253 | 0.236 | 0.200 | 0.210 | 0.176 | 0.228 | 0.340 | **0.384** |
| Junior secondary school sample (*n* = 2110) | | | | | | | | | | |
| Item2 | **0.401** |  |  |  |  |  |  |  |  |  |
| Item5 | 0.279 | **0.503** |  |  |  |  |  |  |  |  |
| Item9 | 0.175 | 0.205 | **0.876** |  |  |  |  |  |  |  |
| Item11 | 0.192 | 0.233 | 0.675 | **0.985** |  |  |  |  |  |  |
| Item13 | 0.153 | 0.161 | 0.302 | 0.341 | **1.048** |  |  |  |  |  |
| Item14 | 0.134 | 0.151 | 0.284 | 0.332 | 0.927 | **1.054** |  |  |  |  |
| Item16 | 0.105 | 0.141 | 0.261 | 0.328 | 0.275 | 0.272 | **0.683** |  |  |  |
| Item21 | 0.111 | 0.150 | 0.220 | 0.272 | 0.178 | 0.168 | 0.194 | **0.499** |  |  |
| Item25 | 0.153 | 0.182 | 0.293 | 0.307 | 0.234 | 0.222 | 0.223 | 0.217 | **0.665** |  |
| Item26 | 0.143 | 0.174 | 0.285 | 0.323 | 0.200 | 0.193 | 0.239 | 0.208 | 0.415 | **0.581** |
| Senior secondary school sample (*n* = 1234) | | | | | | | | | | |
| Item2 | **0.341** |  |  |  |  |  |  |  |  |  |
| Item5 | 0.161 | **0.329** |  |  |  |  |  |  |  |  |
| Item9 | 0.140 | 0.144 | **0.822** |  |  |  |  |  |  |  |
| Item11 | 0.161 | 0.157 | 0.650 | **0.937** |  |  |  |  |  |  |
| Item13 | 0.080 | 0.100 | 0.217 | 0.201 | **0.881** |  |  |  |  |  |
| Item14 | 0.077 | 0.097 | 0.208 | 0.192 | 0.786 | **0.962** |  |  |  |  |
| Item16 | 0.058 | 0.068 | 0.141 | 0.173 | 0.117 | 0.138 | **0.462** |  |  |  |
| Item21 | 0.088 | 0.091 | 0.189 | 0.221 | 0.146 | 0.140 | 0.162 | **0.427** |  |  |
| Item25 | 0.111 | 0.128 | 0.234 | 0.251 | 0.190 | 0.185 | 0.071 | 0.141 | **0.499** |  |
| Item26 | 0.090 | 0.113 | 0.228 | 0.250 | 0.163 | 0.152 | 0.112 | 0.158 | 0.283 | **0.470** |
| *Note*. The diagonal line is the variances, marked in **bold** | | | | | | | | | | |

| **Table H.1** Item covariance matrix of the KIDSCREEN-10 index in the retest survey (*N* = 820) | | | | | | | | | | |
| --- | --- | --- | --- | --- | --- | --- | --- | --- | --- | --- |
|  | **Item2** | **Item5** | **Item9** | **Item11** | **Item13** | **Item14** | **Item16** | **Item21** | **Item25** | **Item26** |
| Item2 | **0.368** |  |  |  |  |  |  |  |  |  |
| Item5 | 0.101 | **0.372** |  |  |  |  |  |  |  |  |
| Item9 | 0.088 | 0.096 | **0.828** |  |  |  |  |  |  |  |
| Item11 | 0.079 | 0.096 | 0.291 | **0.670** |  |  |  |  |  |  |
| Item13 | 0.077 | 0.070 | 0.107 | 0.149 | **0.871** |  |  |  |  |  |
| Item14 | 0.097 | 0.047 | 0.166 | 0.193 | 0.523 | **1.050** |  |  |  |  |
| Item16 | 0.038 | 0.062 | 0.113 | 0.117 | 0.102 | 0.104 | **0.486** |  |  |  |
| Item21 | 0.054 | 0.042 | 0.142 | 0.127 | 0.087 | 0.107 | 0.074 | **0.464** |  |  |
| Item25 | 0.092 | 0.093 | 0.228 | 0.182 | 0.194 | 0.166 | 0.113 | 0.113 | **0.645** |  |
| Item26 | 0.100 | 0.120 | 0.230 | 0.222 | 0.188 | 0.187 | 0.118 | 0.154 | 0.295 | **0.836** |
| *Note*. The diagonal line is the variances, marked in **bold** | | | | | | | | | | |

| **Table H.2** Item covariance matrices of the gender subgroup for the KIDSCREEN-10 index in the retest survey | | | | | | | | | | |
| --- | --- | --- | --- | --- | --- | --- | --- | --- | --- | --- |
|  | **Item2** | **Item5** | **Item9** | **Item11** | **Item13** | **Item14** | **Item16** | **Item21** | **Item25** | **Item26** |
| Male sample (*n* = 396) | | | | | | | | | | |
| Item2 | **0.349** |  |  |  |  |  |  |  |  |  |
| Item5 | 0.093 | **0.357** |  |  |  |  |  |  |  |  |
| Item9 | 0.046 | 0.089 | **0.782** |  |  |  |  |  |  |  |
| Item11 | 0.076 | 0.077 | 0.282 | **0.624** |  |  |  |  |  |  |
| Item13 | 0.084 | 0.083 | 0.144 | 0.240 | **0.927** |  |  |  |  |  |
| Item14 | 0.069 | 0.054 | 0.236 | 0.265 | 0.537 | **1.039** |  |  |  |  |
| Item16 | 0.015 | 0.068 | 0.090 | 0.085 | 0.154 | 0.150 | **0.473** |  |  |  |
| Item21 | 0.058 | 0.058 | 0.149 | 0.140 | 0.090 | 0.140 | 0.084 | **0.488** |  |  |
| Item25 | 0.083 | 0.075 | 0.226 | 0.185 | 0.258 | 0.205 | 0.128 | 0.133 | **0.740** |  |
| Item26 | 0.080 | 0.119 | 0.208 | 0.175 | 0.203 | 0.182 | 0.115 | 0.188 | 0.329 | **0.886** |
| Female sample (*n* = 424) | | | | | | | | | | |
| Item2 | **0.378** |  |  |  |  |  |  |  |  |  |
| Item5 | 0.104 | **0.385** |  |  |  |  |  |  |  |  |
| Item9 | 0.127 | 0.104 | **0.873** |  |  |  |  |  |  |  |
| Item11 | 0.081 | 0.114 | 0.300 | **0.713** |  |  |  |  |  |  |
| Item13 | 0.067 | 0.057 | 0.073 | 0.064 | **0.818** |  |  |  |  |  |
| Item14 | 0.114 | 0.036 | 0.102 | 0.124 | 0.508 | **1.054** |  |  |  |  |
| Item16 | 0.055 | 0.054 | 0.135 | 0.146 | 0.052 | 0.059 | **0.497** |  |  |  |
| Item21 | 0.055 | 0.028 | 0.136 | 0.116 | 0.087 | 0.081 | 0.067 | **0.440** |  |  |
| Item25 | 0.104 | 0.111 | 0.231 | 0.180 | 0.136 | 0.132 | 0.101 | 0.093 | **0.557** |  |
| Item26 | 0.122 | 0.123 | 0.251 | 0.268 | 0.177 | 0.196 | 0.123 | 0.121 | 0.263 | **0.791** |
| *Note*. The diagonal line is the variances, marked in **bold** | | | | | | | | | | |

| **Table H.3** Item covariance matrices of the grade subgroup for the KIDSCREEN-10 index in the retest survey | | | | | | | | | | |
| --- | --- | --- | --- | --- | --- | --- | --- | --- | --- | --- |
|  | **Item2** | **Item5** | **Item9** | **Item11** | **Item13** | **Item14** | **Item16** | **Item21** | **Item25** | **Item26** |
| Primary school sample (*n* = 169) | | | | | | | | | | |
| Item2 | **0.233** |  |  |  |  |  |  |  |  |  |
| Item5 | 0.027 | **0.293** |  |  |  |  |  |  |  |  |
| Item9 | 0.034 | 0.018 | **0.508** |  |  |  |  |  |  |  |
| Item11 | 0.040 | 0.036 | 0.122 | **0.496** |  |  |  |  |  |  |
| Item13 | 0.041 | 0.018 | 0.065 | 0.087 | **0.611** |  |  |  |  |  |
| Item14 | 0.058 | −0.076 | 0.077 | 0.097 | 0.166 | **0.737** |  |  |  |  |
| Item16 | −0.004 | 0.021 | 0.090 | 0.095 | 0.082 | 0.038 | **0.336** |  |  |  |
| Item21 | 0.011 | 0.031 | 0.067 | 0.070 | 0.047 | 0.133 | 0.045 | **0.298** |  |  |
| Item25 | 0.036 | 0.060 | 0.097 | 0.054 | 0.120 | 0.039 | 0.040 | 0.001 | **0.506** |  |
| Item26 | 0.001 | 0.001 | 0.065 | 0.055 | 0.083 | 0.074 | 0.044 | 0.046 | 0.046 | **0.291** |
| Junior secondary school sample (*n* = 368) | | | | | | | | | | |
| Item2 | **0.462** |  |  |  |  |  |  |  |  |  |
| Item5 | 0.147 | **0.455** |  |  |  |  |  |  |  |  |
| Item9 | 0.099 | 0.136 | **0.881** |  |  |  |  |  |  |  |
| Item11 | 0.078 | 0.110 | 0.270 | **0.662** |  |  |  |  |  |  |
| Item13 | 0.087 | 0.037 | 0.116 | 0.168 | **0.877** |  |  |  |  |  |
| Item14 | 0.079 | 0.064 | 0.136 | 0.202 | 0.546 | **1.084** |  |  |  |  |
| Item16 | 0.031 | 0.073 | 0.152 | 0.170 | 0.143 | 0.147 | **0.576** |  |  |  |
| Item21 | 0.072 | 0.048 | 0.191 | 0.143 | 0.148 | 0.124 | 0.075 | **0.541** |  |  |
| Item25 | 0.121 | 0.108 | 0.307 | 0.260 | 0.194 | 0.146 | 0.183 | 0.174 | **0.834** |  |
| Item26 | 0.104 | 0.130 | 0.295 | 0.262 | 0.150 | 0.124 | 0.141 | 0.219 | 0.393 | **0.937** |
| Senior secondary school sample (*n* = 283) | | | | | | | | | | |
| Item2 | **0.290** |  |  |  |  |  |  |  |  |  |
| Item5 | 0.059 | **0.294** |  |  |  |  |  |  |  |  |
| Item9 | 0.032 | 0.042 | **0.820** |  |  |  |  |  |  |  |
| Item11 | 0.073 | 0.089 | 0.351 | **0.736** |  |  |  |  |  |  |
| Item13 | 0.026 | 0.100 | −0.006 | 0.076 | **0.879** |  |  |  |  |  |
| Item14 | 0.058 | 0.033 | 0.085 | 0.120 | 0.511 | **0.926** |  |  |  |  |
| Item16 | 0.035 | 0.049 | 0.017 | 0.055 | 0.036 | 0.053 | **0.403** |  |  |  |
| Item21 | 0.034 | 0.026 | 0.090 | 0.134 | 0.014 | 0.045 | 0.062 | **0.452** |  |  |
| Item25 | 0.035 | 0.054 | 0.102 | 0.092 | 0.128 | 0.115 | 0.038 | 0.082 | **0.400** |  |
| Item26 | 0.083 | 0.129 | 0.109 | 0.192 | 0.164 | 0.147 | 0.088 | 0.106 | 0.208 | **0.896** |
| *Note*. The diagonal line is the variances, marked in **bold** | | | | | | | | | | |
